# Nanoscale Direct-to-Biology Optimization and Structural Insights into Selective *S. aureus* TrmD Inhibitors

**DOI:** 10.1021/acs.jmedchem.5c02323

**Published:** 2025-12-10

**Authors:** Ariane F. Hübner, Annabelle C. Weldert, Tessa Marciniak, Florian Hof, Vivien S. Beck, Samuel Carien, Sophie N. Mulartschyk, Eva Wolf, Wilma Ziebuhr, Fabian Barthels

**Affiliations:** † Institute of Pharmaceutical and Biomedical Sciences, 9182 Johannes Gutenberg-University, Staudingerweg 5, 55128 Mainz, Germany; ‡ Institute of Molecular Infection Biology, 9190 University of Würzburg, Josef-Schneider-Strasse 2, 97080 Würzburg, Germany; § Institute of Molecular Physiology, Johannes Gutenberg-University, Hanns-Dieter-Hüsch-Weg 17, 55128 Mainz, Germany

## Abstract

The tRNA m^1^G37 methyltransferase (TrmD) is considered essential in various bacteria, including *Staphylococcus aureus*, a pathogen responsible for a wide range of diseases. Here, we have performed a high-throughput nanomole-scale synthesis campaign (nanoSAR) by late-stage copper­(I)-catalyzed alkyne–azide cycloaddition (CuAAC)-functionalizing a library of structurally diverse azides (N = 320) to a pyrrolopyrimidone alkyne. We have identified selective *S. aureus* TrmD inhibitors with inhibitory activity in the nanomolar to low micromolar range using a direct-to-biology assay read-out. A carbamate-masked guanidine intermediate of the lead structure selectively inhibited *S. aureus* growth at low micromolar concentrations in cell-based assays, while Gram-negative bacteria and an off-target panel of methyltransferases were not affected. Subsequent cocrystallization resulted in a crystal structure of *S. aureus* TrmD bound to an inhibitor, providing detailed insights into its binding mode and enabling future structure-guided optimization.

## Graphical abstract

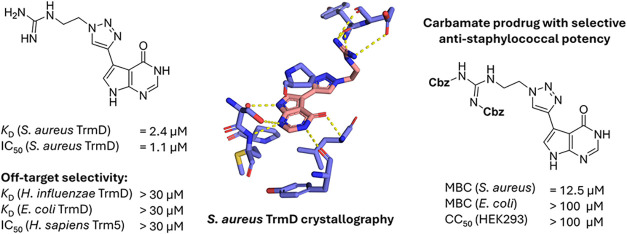

## Introduction

*Staphylococcus aureus* is a prevalent bacterial pathogen, causing a broad spectrum of diseases ranging from skin infections to life-threatening conditions such as pneumonia and sepsis.^1^ The treatment of *S. aureus* infections is increasingly difficult due to the antibiotic resistance of the pathogen, including the emergence of multidrug-resistant isolates.^2^ Particularly, methicillin-resistant *S. aureus* (MRSA) currently represents the deadliest resistance-pathogen pairing globally, causing more than 100,000 deaths in 2019 alone.^3,4^ Therefore, the identification of new antimicrobial drugs is strongly desired to overcome resistance issues in *S. aureus*.

One enzyme essential for *S. aureus* among many other bacteria is the tRNA m^1^G37 methyltransferase (TrmD), which catalyzes the methyl group transfer from *S*-adenosylmethionine (SAM) to the *N* ^1^ position of guanosine 37 in a defined subset of tRNAs.^5–8^ Since this modification is located in close proximity to the 3′-side of the anticodon, its absence can lead to tRNA misrecognition during translation and +1-frameshift errors during protein synthesis.^9,10^ In eukaryotes and archaea, the m^1^G37 modification is introduced by the structurally unrelated tRNA methyltransferase 5 (Trm5).^11,12^ Trm5 belongs to class I of methyltransferases, while bacterial TrmD belongs to the unique class IV, also known as SPOUT class.^11^ TrmD was identified as a potentially selective antimicrobial drug target, due to its essential role as well as the structural differences from Trm5.^13^ Crystal structures of inhibitor-bound TrmD complexes from several organisms, including *Haemophilus influenzae* and *Escherichia coli*, have been solved in the past, offering starting points for drug discovery campaigns.^14–17^

To date, a limited number of TrmD inhibitors targeting diverse bacterial organisms have been reported (15 publications since 2011, according to a PubMed search using the keyword “TrmD Inhibitor”),^14,18–20^ but so far, only very few TrmD inhibitors have been effective in targeting *S. aureus*.^16^ Despite the urgent need for new antibiotics targeting *S. aureus*, previous studies focused mainly on developing TrmD inhibitors for *H. influenzae*, *E. coli*, *Mycobacterium abscessus*, and *Mycobacterium tuberculosis*.^14,18–20^ In a development campaign conducted by AstraZeneca, Hill et al. screened a fragment collection to identify *H. influenzae* TrmD inhibitors.^14^ They investigated thienopyrimidone-based inhibitors that showed inhibitory activity against a panel of TrmD orthologs (*E. coli*, *H. influenzae*, *Acinetobacter baumanni*, *Klebsiella pneumoniae*, and *Pseudomonas aeruginosa*), offering broadband starting points for drug discovery, but this also suggested only limited interspecies selectivity.^14^

To target this gap, in this present study, we employed the thienopyrimidone scaffold discovered by Hill et al. and optimized the inhibitory profile to develop selective inhibitors targeting *S. aureus* TrmD using a high-throughput nanomole-scale (final: 96 nmol) synthesis strategy. Reactions conducted with less than 300 nmol of starting material are commonly referred to as being performed on the nanomole scale.^21^ This miniaturized format offers advantages compared to flask-oriented synthesis campaigns in throughput, development time, and in conserving valuable synthetic intermediates.^22^ Notable contributions in this field feature the implementation of a range of standard medicinal chemistry transformations in plate-based formats, including the development of a copper-catalyzed alkyne–azide click reaction (CuAAC) pipeline as described by Gehrtz et al., suitable for our inhibitor development campaign.^21,23–25^ If the resulting crude nanomole-scale reaction mixtures are directly analyzed using *in vitro* or *in cellulo* assays to derive structure–activity relationship (SAR) insights, this strategy was coined by several recent reports (2021−) as a *direct-to-biology* (D2B) approach.^26–30^

## Results and Discussion

### Definition of an *S. aureus* Targeting Lead Structure

To date, no dedicated *S. aureus* TrmD inhibitors have been reported. Hence, to define a suitable lead for nanoSAR-based optimization, we analyzed literature-known ligands of TrmD isoenzymes from diverse microorganisms. Hill et al. discovered the thienopyrimidone inhibitor **1** by screening a collection of fragments against *H. influenzae* TrmD, and their subsequent optimization resulted in inhibitor **2** with activities in the nanomolar to micromolar range.^14^ X-ray cocrystal structures with *H. influenzae* TrmD were previously solved for inhibitor **1** (PDB: 4MCD) and the optimized *H. influenzae* inhibitor **2** (PDB: 4MCC). Both inhibitors were found to occupy the SAM binding pocket of TrmD (Figure 1A), while the thienopyrimidone moieties targeted the adenine binding pocket, and the phenyl ring of inhibitor **1** extended into the ribose pocket (Figure 1A, Figure S1A). The phenylmethylamine moiety of inhibitor **2** reaches further into the methionine part of the SAM pocket (Figure S1B), where an additional interaction with Glu116 can be formed (Figure 1A).^14^ Due to the considerable sequence similarity (65%) between *H. influenzae* TrmD and *S. aureus* TrmD, we hypothesized that this thienopyrimidone chemotype might provide a reasonable lead for *S. aureus* drug development. To assess if the thienopyrimidone-based compounds, developed as dedicated *H. influenzae* TrmD inhibitors, are suitable starting points for targeting the *S. aureus* orthologous enzyme, we evaluated the binding thermodynamics of **1** and **2** via isothermal titration calorimetry (ITC) on the recombinant *S. aureus* TrmD enzyme (Figure 1B,C, Figure S2). **1** and **2** showed enthalpy-dominated binding profiles in the micromolar range on both *H. influenzae* and *S. aureus* enzymes, with an unfavorable selectivity toward targeting *S. aureus* TrmD for **2**. The determined dissociation constants were 19.8 and 12.9 μM, respectively, and thus, were deemed suitable as starting points for *S. aureus* targeting ligand optimization.

**Figure 1:**
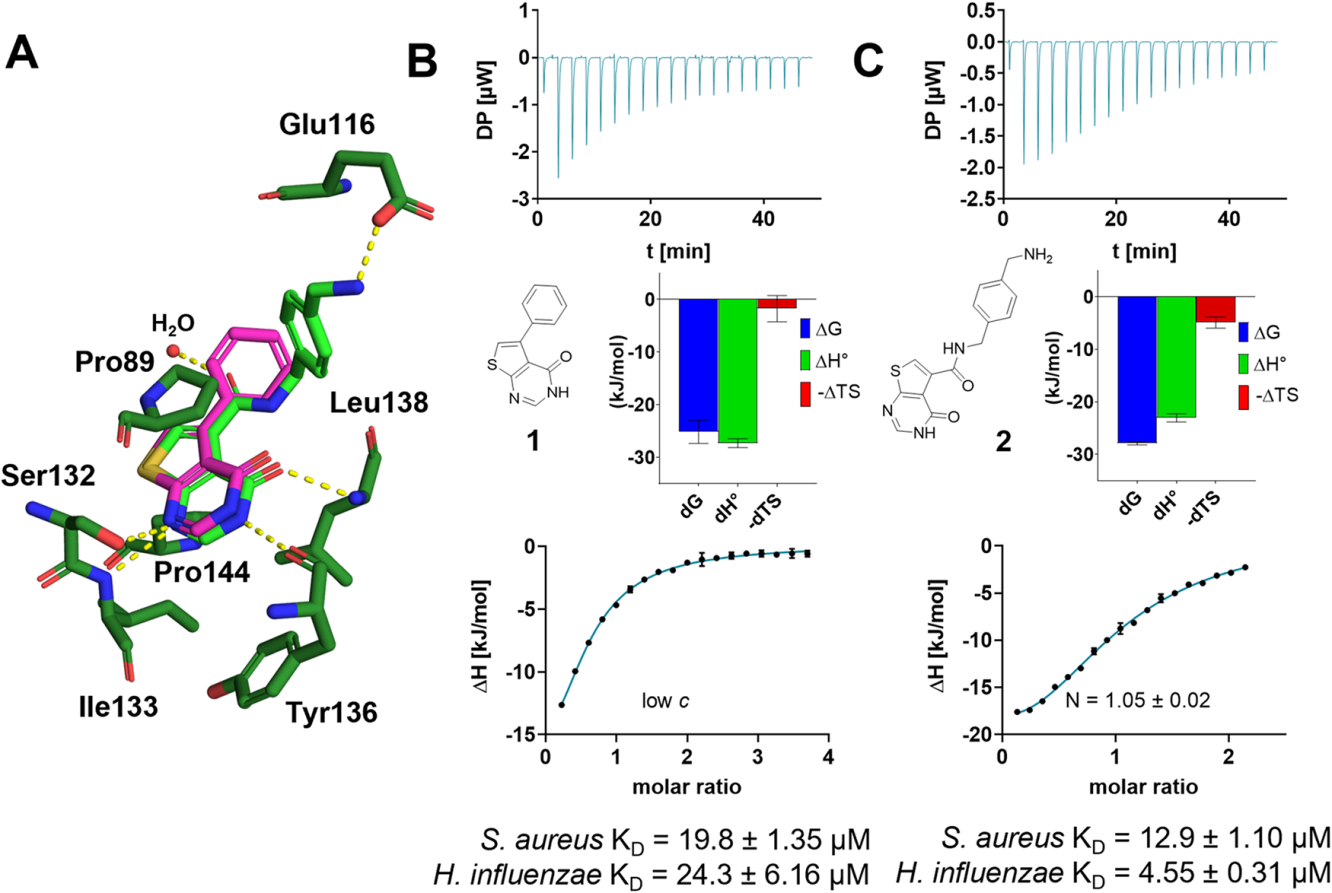
Definition of starting points for the development of *S. aureus* TrmD inhibitors. (A) Crystallographic overlay of literature-known inhibitors **1** (PDB: 4MCD) and **2** (PDB: 4MCC) in the *H. influenzae* SAM-binding pocket. Ligands and amino acids are shown in stick representation; polar interactions are shown as yellow lines, and water as red spheres. The carbon atoms of **1** are shown in pink and those of compound **2** in light green. Interacting residues are displayed in dark green. (B, C) Structures and affinities of inhibitors **1** (B) and **2** (C) binding to *S. aureus* TrmD determined by ITC, incl. thermograms, stoichiometry, and signature plots (data for *H. influenzae* TrmD in Figure S2). Due to low enthalpy signals, a low *c*-titration was performed for inhibitor **1**, resulting in *N* < 1.

### Development of a Direct-to-Biology Assay Platform for Inhibitor Optimization

To enable the screening of hundreds of nanomole-scale synthesized compounds, the implementation of a high-throughput-capable RNA methyltransferase (MTase) assay is considered fundamental. In a previous publication, we demonstrated the suitability of fluorescence polarization (FP)-based assays as a screening method to identify and characterize MTase inhibitors.^31^ In this assay format, a fluorescent, SAM-pocket binding tracer can be displaced by the compound under investigation, and the resulting change in fluorescence polarization defines the read-out of the inhibitor compound’s affinity (Figure 2A).

**Figure 2:**
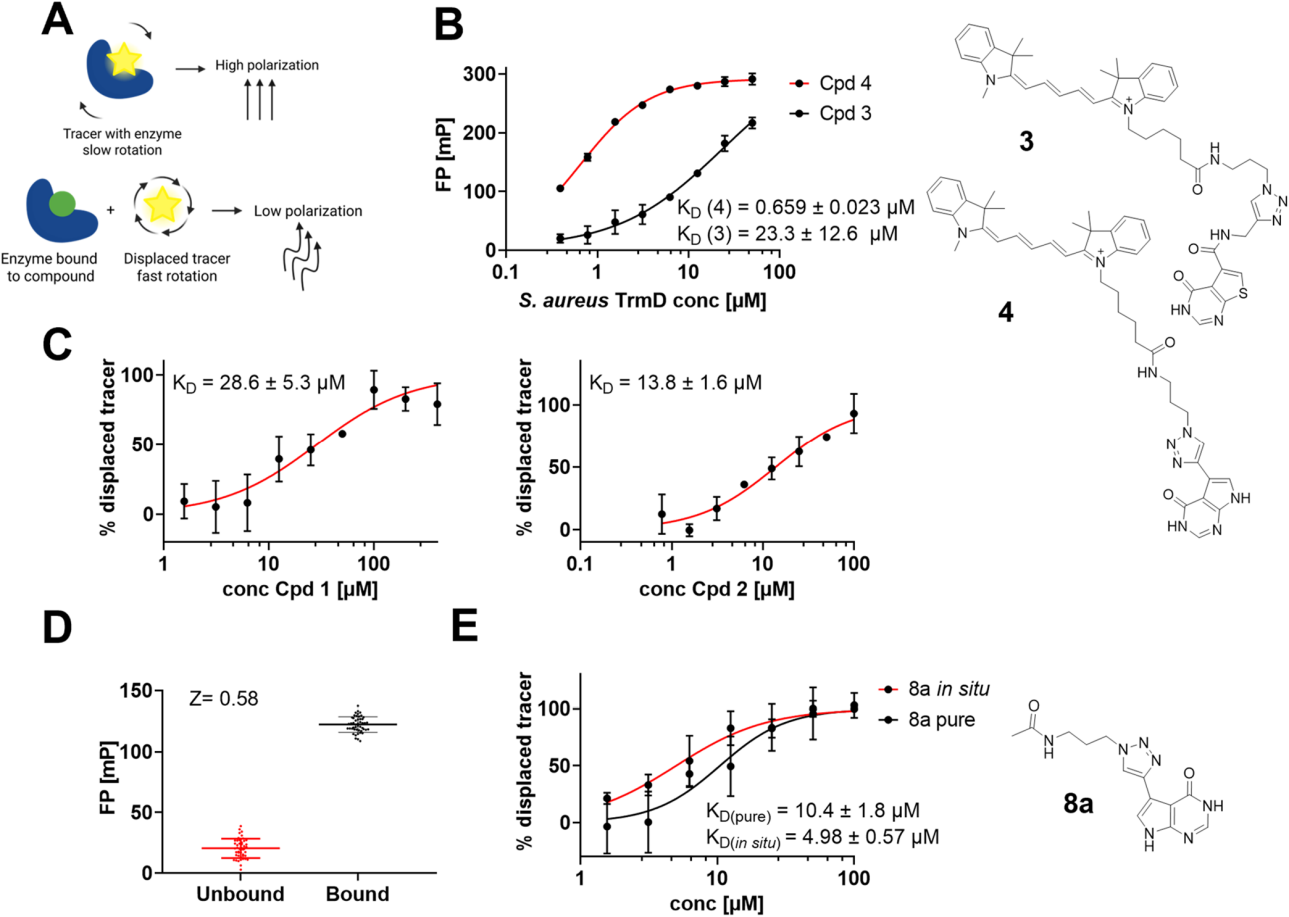
Fluorescence polarization (FP) assays for *S. aureus* TrmD ligand investigation. (A) Concept of the FP displacement assay. (B) Chemical structures and FP-derived affinity of tracers **3** and **4** binding to *S. aureus* TrmD. (C) FP displacement assays for the determination of apparent affinities of parent compounds **1** and **2**. (D) Evaluation of statistical *Z*-factors to assess the high-throughput capability of this FP assay setup. (E) FP displacement assays of nanoSAR test triazole **8a** *in situ* vs chromatographically purified compound.

Previously, we developed an *H. influenzae* TrmD targeting fluorescent tracer **3**, which we originally intended to adapt for *S. aureus* TrmD.^31^ Compared to *H. influenzae* TrmD (*K*_D_ = 2.0 μM, Figure S4A), however, this tracer showed only weak affinity for the *S. aureus* orthologous enzyme (*K*_D_ = 23.3 μM, Figure 2B). To address this issue, we employed scaffold hopping from a thieno- to a pyrrolopyrimidone, as demonstrated in the study by Hill et al., which resulted in improved binding affinities for *S. aureus* TrmD compared to *H. influenzae* TrmD. Thus, here, we report a new fluorescent tracer **4** dedicated to *S. aureus* TrmD with a pyrrolopyrimidone motif and without the amide linker (Figure 2B). Evaluation of tracer **4** in FP assays confirmed this hypothesis and revealed a *K*_D_-value of 659 nM for *S. aureus* TrmD, suitable for the establishment of a high-throughput-capable assay format (Figure 2B). The structural reason for the preference of the thienopyrimidone motif and an amide-free linker for *S. aureus* TrmD could be rationalized by crystallographic analysis of our *S. aureus* TrmD inhibitor complexes (*vide infra*).

To validate that optimized *S. aureus* tracer **4** is suitable for a direct-to-biology screening of TrmD inhibitors, we investigated dose–response series of parent compounds **1** and **2** by FP displacement assays, yielding apparent binding affinities (*K*_D_ = 28.6 and 13.8 μM), and hence, agreeing with the magnitude and relative order determined via ITC (Figure 2C vs Figure 1B,C). Furthermore, we were able to confirm that the assay setup consisting of *S. aureus* TrmD (500 nM) and tracer **4** (10 nM) is suitable as a high-throughput-capable format by determining the assay-specific *Z*-factor (*Z* = 0.58, Figure 2D). The *Z*-factor is a statistical parameter that assesses the dynamic range of an assay to determine its suitability for HTS; a *Z*-factor >0.5 is generally considered indicative of an excellent assay.^32^ Based on the assay results gleaned from the tracer experiments, we decided to use the alkyne handle of the compound **4** precursor (cpd **7**) to build a CuAAC-derived nanoSAR triazole library for the development of new *S. aureus* selective TrmD inhibitors. The workflow is illustrated in Figure 3.

**Figure 3:**
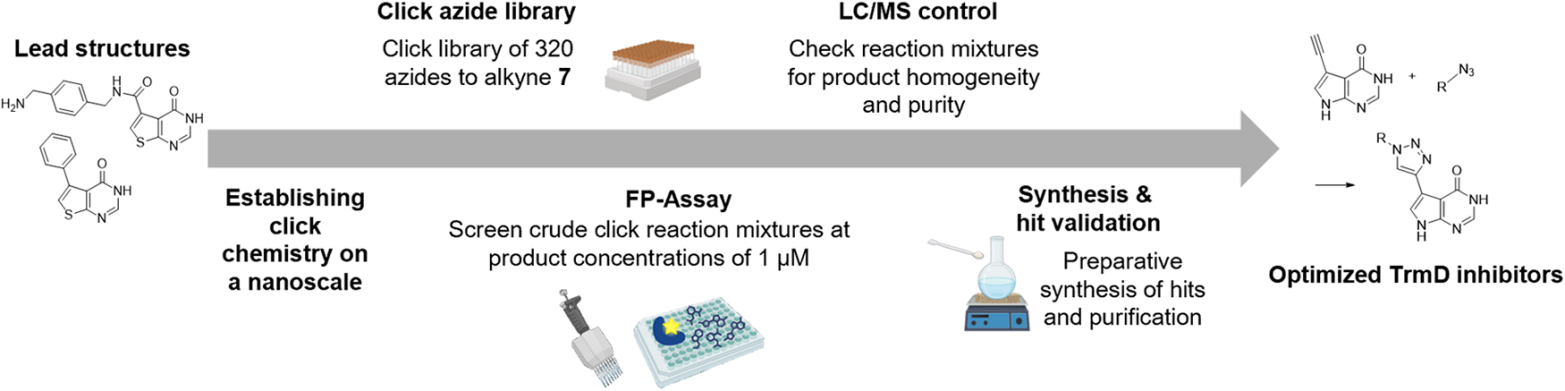
Workflow of the nanoSAR strategy for optimization of *S. aureus* TrmD inhibitors. Reaction of the alkyne precursors **7** with 320 chemically diverse azides resulted in a plate-based library of inhibitor triazoles, which were measured in a direct-to-biology FP displacement assay, followed by an LC/MS confirmation of hit compounds’ crude reactions, and subsequently, the preparative synthesis and validation of the selected candidate compounds.

To confirm that alkyne **7** (Scheme 1) and the resulting crude nanomole-scale triazoles are compatible with the established direct-to-biology platform, we performed a pilot exploration including the *in situ* synthesis and screening of a representative triazole candidate **8a** (Figure 2E). LC/MS reaction control of pilot alkyne–azide coupling reactions showed consistently >90% conversion rates and no production of side-products; thus, we concluded that this D2B outline is suitable for the generation of a TrmD inhibitor library. The nanomole scale reaction mixture was subsequently tested by FP assay both in crude and after chromatographic purification of this test compound, revealing only slight variation in *K*_D_-values of 4.98 and 10.4 μM, respectively, along with a minimal variation of the Hill slope (Figure 2E). This suggests that crude reactions from the azide library provide a reliable semiquantitative indication of binding affinities, as the objective of D2B screenings is to rank the potency of substituents in relative order. Also, the FP assay setup using tracer **4** and *S. aureus* TrmD was found to be insensitive toward all components of the *in situ* click reaction, including unreacted alkyne **7** (*K*_D_ > 100 μM, Figure S4B).

**Scheme 1:**
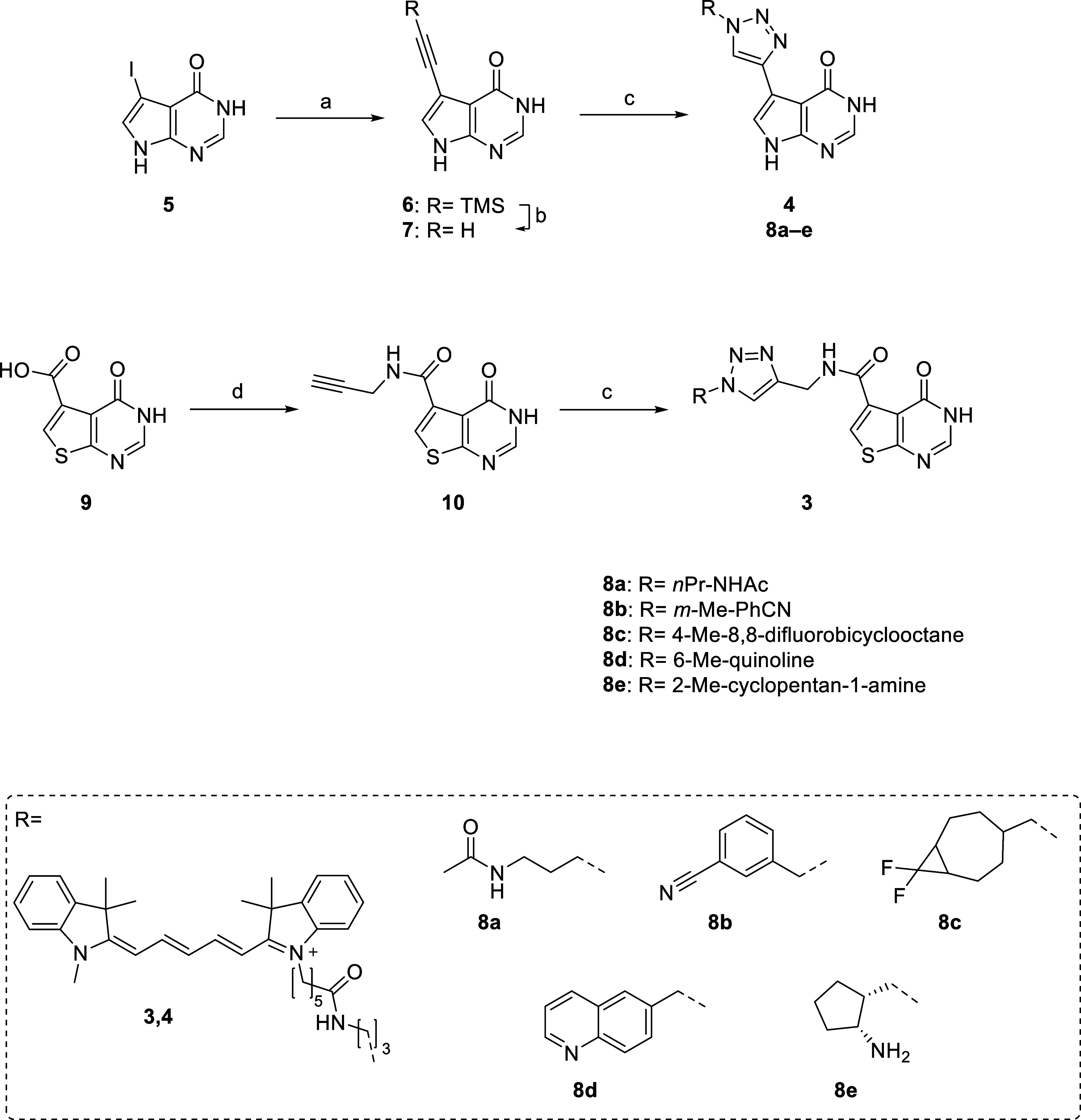
Overview of Synthetic Procedures^a^

a Reagents and conditions: (a) CuI, TMS-acetylene, Pd­(PPh_3_)_4_, triethylamine, DMF/THF, rt, 16 h, 29%; (b) 1 M tetra-*n*-butylammonium fluoride, THF/ACN, 0 °C–rt, 1.5 h, 68%; (c) CuSO_4_×5 H_2_O, TBTA, sodium ascorbate, DMSO, H_2_O, rt, 16 h, 25–74%; (d) HATU, DIPEA, DCM/DMF, rt, 16 h, 77%–quant.

### Chemistry

In this present study, a new alkyne-decorated pyrrolo­[2,3-*d*]­pyrimidone with similar chemical properties to parent structures **1** and **2** was synthesized (Scheme 1) for the preparation of triazole-based *S. aureus* TrmD inhibitors **8a–e**.

For inhibitor generation, this precursor alkyne **7** was subsequently reacted with diverse aromatic and aliphatic azides via copper­(I)-catalyzed azide–alkyne cycloaddition (CuAAC). The thieno-subunit from literature TrmD inhibitors (e.g., **1** and **2**) was substituted with a pyrrolo-unit to facilitate synthesis, without compromising binding affinity. According to Hill et al., replacing the sulfur atom in the thienopyrimidone moiety of the scaffold with a nitrogen was favorable for *S. aureus* selectivity, which allowed us to develop a high-affinity fluorescent tracer **4** (Figure 2).^14^ The unoccupied TrmD methionine pocket (Figure S1) leaves space for modifications at the pyrrolo unit, and thus, we synthesized triazole derivatives for both tracer and inhibitor development. The synthetic routes of compounds **6**–**14**, including the fluorescent tracers and the compounds originating from nanoSAR screening, are described in Schemes 1 and 2.

**Scheme 2:**
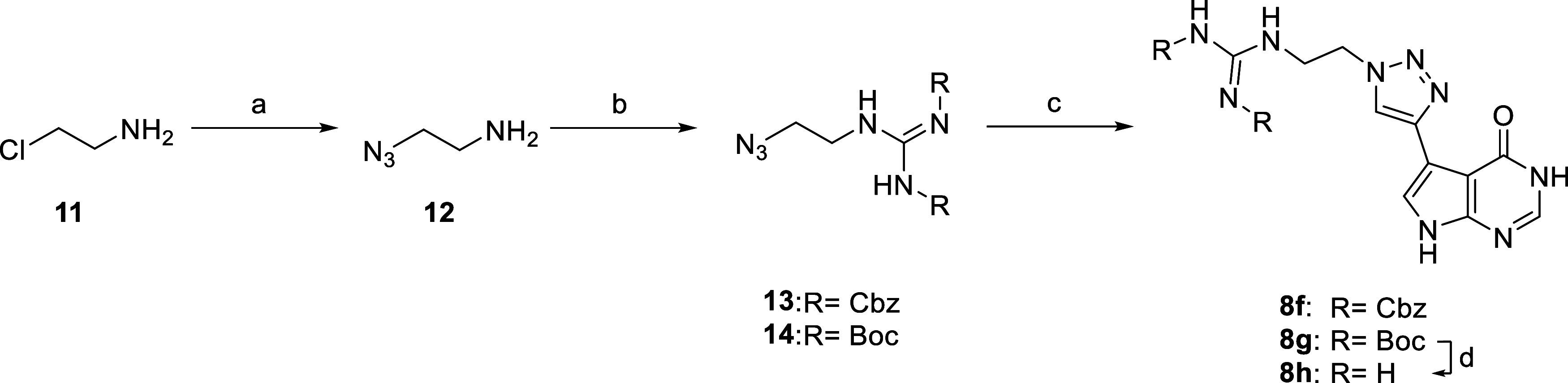
Overview of the Synthetic Procedure for Compounds **8h**–**g** ^a^

a (a) Sodium azide, water, 90 °C, 16 h, quant.; (b) *N*,*N*′-Bis-Boc- or *N*,*N*′-Bis-Cbz-guanylpyrazole, THF, triethylamine, rt, 72 h, 47–90%; (c) CuSO_4_×5 H_2_O, TBTA, sodium ascorbate, DMSO, H_2_O, rt, 16 h, 25–74%; (d) TFA, DCM, 30 min, rt, quant.

Literature-known compound **2** was synthesized by a HATU-mediated coupling of *tert*-butyl (4-(aminomethyl)­benzyl)­carbamate and 4-oxo-3,4-dihydrothieno­[2,3-*d*]­pyrimidine-5-carboxylic acid, followed by a Boc-deprotection with TFA in DCM. The synthesis route for alkyne **7** started with a Sonogashira reaction using commercially available 5-iodo-3,7-dihydro-4*H*-pyrrolo­[2,3-*d*]­pyrimidin-4-one (**5**) and TMS-acetylene, catalyzed by Tetrakis­(triphenylphosphine)­palladium(0) and copper­(I) iodide, yielding compound **6**. Finally, alkyne **7** was obtained by deprotection of the TMS-group by tetra-*n*-butylammonium fluoride (TBAF). Subsequent CuAAC with alkyne **7** and various azides was performed using CuSO_4_×5 H_2_O, which was reduced *in situ* to copper­(I) by sodium ascorbate and stabilized by *tris*((1-benzyl-4-triazolyl)­methyl)­amine (TBTA) in DMSO and water (1:1).^21^ Hit compounds of the nanoSAR study and fluorescent tracers were resynthesized by the same procedure on a preparative scale and purified by reverse-phase flash chromatography.

Preparative synthesis of **8h** started with 2-chloroethylamine (**11**), which was converted to the corresponding azide (**12**) with sodium azide. To introduce the guanidine group, *N*,*N*′-Bis-Boc-1-guanylpyrazole was reacted under basic conditions in THF to afford intermediate **14**. Subsequently, intermediate **14** was reacted with alkyne **7** via CuAAC to yield **8g**. Under similar conditions, compound **8f** was synthesized with *N*,*N*′-Bis-Cbz-1-guanylpyrazole. Final deprotection of the Boc-group of **8g** with TFA and DCM (1:1) yielded lead compound **8h**.

### Preparation of a nanoSAR Triazole Library and FP-Based TrmD Screening

Next, we conducted an *S. aureus* TrmD-targeting nanoSAR study. For this, alkyne **7** was reacted on a 96 nmol scale with an in-house diversity-oriented azide library (N = 320, mean pairwise Tanimoto similarity = 0.11) designed to cover a large chemical fragment space, generating a panel of 320 crude triazoles ready for FP assay investigation. The composition of the azide library is designed to be chemically diverse in several categories, including physicochemical properties such as polar surface area, lipophilicity, and the number of hydrogen bond donors and acceptors, as well as structural features like the number of aromatic rings (Figure S23). A complete list of chemical structures included in the azide library can be found in Table S4. *In situ* reaction conditions for CuAAC were adapted from Gehrtz et al., utilizing CuSO_4_, which is reduced *in situ* to Cu­(I) by sodium ascorbate and stabilized by TBTA.^21^ Degassed water and DMSO (1:1) were used as solvents to prevent the oxidation from Cu­(I) to Cu­(II). For the synthesis of the triazole library, a master mix was prepared, containing all reaction components except the azides. All reactions were started by the addition of the respective azide in 24 μL at a final product concentration of 4 mM (96 nmol). Reaction mixtures were incubated for 16 h at room temperature and under an argon atmosphere in sealed 96-well plates.

Based on the pilot explorations of pyrrolopyrimidine triazoles **4** and **8a** (*K*_D_ = 0.66 and 4.98 μM), we adjusted the final concentration of the triazoles for FP screening to 1 μM, assuming full conversion of the click reaction. In this regard, conversion rates were tested for 24 random reaction mixtures by LC/MS and were found consistently to be >90%. For FP assay screening, we used 500 nM of *S. aureus* TrmD and 10 nM of tracer **4** in a total volume of 40 μL in 96-well plates. We performed control measurements and defined 10 nM of tracer **4** in buffer as 100% displacement and the DMSO control as 0% tracer displacement. As primary hits, we intended to select the top 5 compounds that exhibited tracer displacements >93%. The results of the FP screening were visualized in a waterfall plot and plate-based heatmaps (Figure 4, Figure S3).

**Figure 4:**
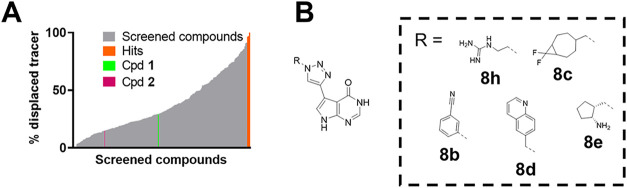
Results, analysis, and hit selection of the nanoSAR TrmD screening. (A) Waterfall plot summarizing the screening of 320 triazole inhibitor candidates at 1 μM. In orange, the five compounds (“hits”) with the highest potency (>93% displaced tracer, **8b**–**e,h**) are shown. (B) Chemical structure representation of the five hits **8b**–**e,h** that were identified during screening.

Five compounds (**8b**–**e,h**) showed near complete displacement during FP screening at an inhibitor concentration of 1 μM, and consequently, these discovered structures (“hits”) were verified for reaction conversion and identity via LC/MS (>90% crude purity). A comprehensive overview of the FP assay results for all compounds generated from the azide library can be found in Table S4. Structurally, hits **8b**–**e,h** appear to have limited similarity; yet **8b**–**d** contain hydrophobic, sterically demanding decorations, whereas **8b,e,h** share a basic amine or amidine moiety, and thus, these five hit compounds (**8b**–**e,h**) were selected for preparative resynthesis and validation. SAR of these enriched ligand features is discussed by crystallography and molecular modeling (*vide infra*).

### Resynthesis and Characterization of *S. aureus* TrmD Inhibitors

The five hit compounds identified during the FP screening were subsequently resynthesized on a preparative scale, and binding profiles were validated using the FP assay with >95% chromatographically purified inhibitors. Synthesis procedures of the hits can be found in Schemes 1 and 2. *K*_D_-values and the respective curves for all hit compounds **8b**–**e,h** were determined by dose–response experiments in the FP assay and can be found in Figure 5 and Table 1. To further investigate the inhibitory efficacy of **8b**–**e,h**, we utilized a literature-known aptamer-based *S. aureus* TrmD enzyme assay for IC_50_ determination (Table 1) and an orthogonal label-free ^3^H-scintillation MTase assay (Figures S9, S10).^33^ Both *K*_D_ and IC_50_ values ranged from 390 nM to 3.22 μM, and in summary, it can be concluded that the nanoSAR strategy has led to a successful optimization of parent compounds **1** and **2** (28.6 and 13.8 μM).

**Figure 5:**
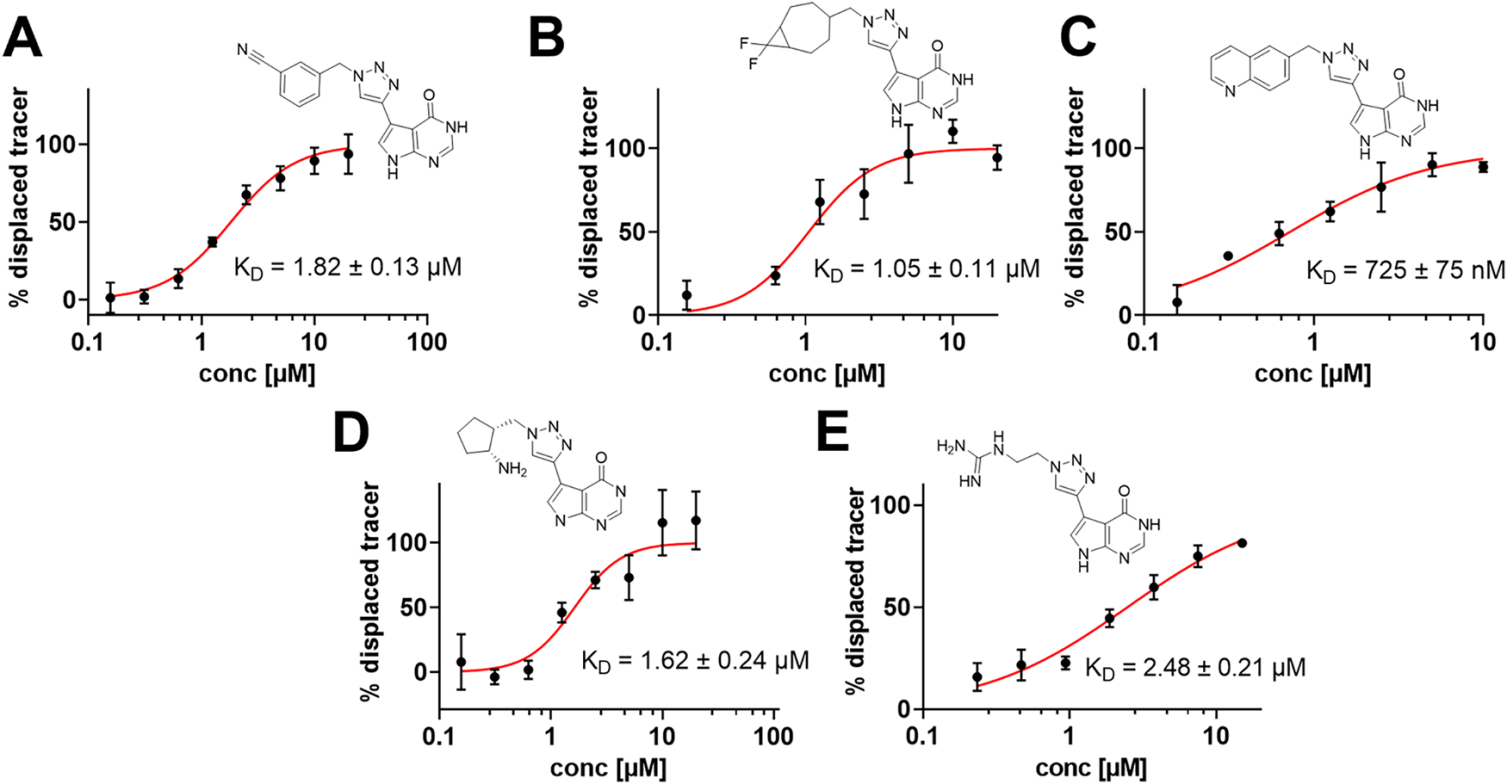
FP-assay of chromatographically purified (A) **8b** (B) **8c** (C) **8d** (D) **8e** (E) **8h** with the respective structures and *K*_D_-values.

**Table 1:** Overview of *K*_D_-Values (Determined by FP Assays) and IC_50_-Values (Determined by Aptamer-Based Enzyme Assays for *S. aureus* TrmD and ^3^H-Based Enzyme Assay for Trm5) for **8b**–**e,h** ^a^

	*S. aureus*		*E. coli*	*H. influenzae*	Trm5
Cpd	*K*_D_ [μM]	IC_50_ [μM]	*K*_D_ [μM]	*K*_D_ [μM]	IC_50_ [μM]
**8a**	10.4 ± 1.8	n.d.	n.d.	n.d.	n.d.
**8b**	1.82 ± 0.13	3.22 ± 1.80	>30	>30	>30
**8c**	1.05 ± 0.11	0.71 ± 0.10	>30	>30	>30
**8d**	0.72 ± 0.07	0.39 ± 0.07	>30	>30	>30
**8e**	1.62 ± 0.24	0.48 ± 0.06	>30	>30	>30
**8h**	2.48 ± 0.21	1.16 ± 0.12	>30	>30	>30

a n.d.: not determined.

Next, we employed the FP-assay using tracers **3** and **4** to test the selectivity of **8b**–**e,h** against a panel of TrmD isoenzymes from *S. aureus*, *E. coli*, and *H. influenzae* (Figures S7, S8). Furthermore, we tested the inhibitory potency of **8b**–**e,h** for the human MTase homologue Trm5 (Figure S6). Since no suitable fluorescent tracer was available, a functional ^3^H-based enzyme assay was performed to assess Trm5 selectivity. All hit compounds exhibited at least >10× *K*_D_- or IC_50_-values for all off-targets compared to the *S. aureus* TrmD, and thus, we conclude that nanoSAR optimization provided not only affine but also selective *S. aureus* TrmD inhibitors (Table 1). In summary, the hit compounds **8b**–**e,h** demonstrated a significant improvement regarding selectivity and affinity toward the *S. aureus* TrmD compared to the parent compounds **1** and **2**. Also, we investigated mammalian cytotoxicity of the hits **8b**–**e,h** using CellTiterGlo assays following cell viability in HEK293 cells. In summary, the hit compounds showed no significant effects on cell viability at relevant concentrations up to 100 μM (Figure S11). Slight cytotoxic effects were only observed for compounds **8b** and **8c** at 100 μM, and for **8e** at >10 μM, whereas the identified lead *S. aureus* TrmD inhibitor compound **8h** showed no mammalian cytotoxicity up to 100 μM.

### Antibacterial Assays and Potential Prodrug Identification

To assess the antibacterial activity of **8b**–**e,h**, the effect on *S. aureus* RN4220 was investigated using a dose–response bacterial growth inhibition assay. Also, two synthetic carbamate intermediates (**8f** and **8g**, Figure 6) obtained during the preparation of **8b**–**e,h** were included in this assay through their similarity with the hit compounds. Here, initial screenings of **8b**–**h** were conducted at 1, 10, and 100 μM but revealed that the growth of *S. aureus* was not affected by most compounds at concentrations up to 100 μM compared to the untreated controls in bacterial growth inhibition (Figure 6, details for hit compounds from the library **8b-e,h** are shown in Figure S12). Also, none of the compounds showed significant growth inhibition for *E. coli* (Figures S13, S14). As TrmD is an essential protein in *S. aureus*, successful inhibition is expected to lead to cell death and/or growth inhibition, which has been substantiated in numerous literature studies to date.^5,8,34^ Yet, it has been observed in similar TrmD drug development campaigns that, despite nanomolar affinity, a majority of TrmD inhibitors fail in terms of antibacterial potency, presumably due to their large polar surface area and insufficient permeability through the bacterial cell wall.^14,18–20,35^ Surprisingly, a significant reduction in *S. aureus* growth was observed during treatment of RN4220 with di-Cbz intermediate **8f** (Figure 6A, Figure S15B: MIC = 12.5 μM), while **8f** and **8g** showed no inhibition of Gram-negative *E. coli* (Figures S13, S14). In fact, a slight increase in *E. coli* growth rate at the highest concentration (100 μM) of **8f** could be observed, possibly due to a compensatory effect of this more permeable drug acting on an unknown Gram-negative off-target (Figure S14B).

**Figure 6:**
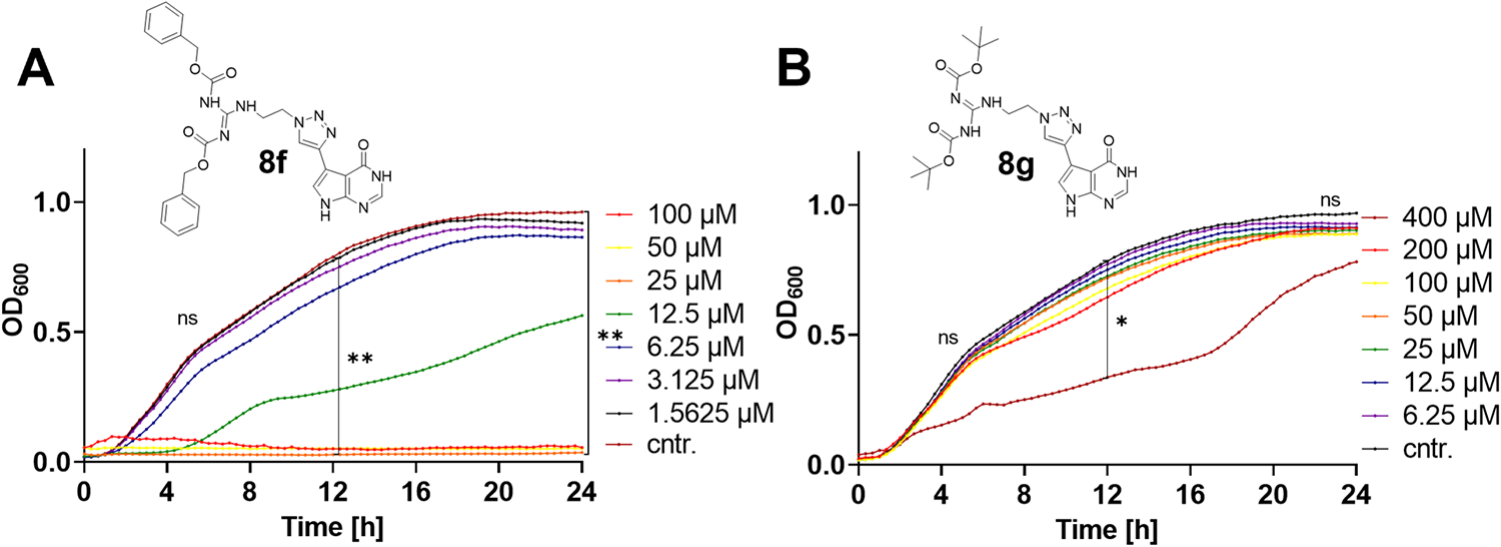
*S. aureus* growth inhibition by **8f** (di-Cbz) and **8g** (di-Boc). (A) *S. aureus* RN4220 treated with **8f** and (B) *S. aureus* RN4220 treated with **8g**. Only **8f** showed a significant effect on *S. aureus* bacteria in lower concentrations. Two-way ANOVA with Dunnett’s multiple comparisons test was used for statistical analysis, using a confidence interval of 95% (Time points: 6, 12, and 24 h).

We hypothesized that the activity of **8f** against *S. aureus* could be attributed to the di-Cbz carbamate moiety, masking the large polar surface area of the guanidine motif of hit **8h**, which itself did not influence bacterial growth, presumably due to the charged nature and limited permeability (Figures S12, S13). The strategy of conjugating a lipophilic tag has previously been shown to improve the permeability of antibacterials.^16,36,37^ To determine the minimal bactericidal concentration (MBC) of the substances, bacterial cultures were prepared as in the bacterial growth inhibition assay. While cell growth is not negatively affected by treatment with Boc-carbamate **8g**, and more CFU/mL were observed after incubation, Cbz-carbamate **8f** affects growth starting at 12.5 μM treatment (Figure S16). On average, fewer CFU/mL were determined after 24 h of incubation, but bacteria were not killed completely. This indicates a rather bacteriostatic mode of action.

Subsequently, both synthetic intermediates **8f** (di-Cbz) and **8g** (di-Boc) were evaluated for their *in vitro* TrmD affinity using FP assays. **8g** displayed a *K*_D_-value of 19.8 μM, while **8f** showed no *S. aureus* TrmD binding up to 100 μM (Figure S5A,B), and thus, we concluded that the antistaphylococcal activity of **8f** is not mediated by direct TrmD inhibition. Hence, we developed a mechanistic hypothesis evaluated below: Since carbamates are known to be susceptible to cleavage by esterases, we hypothesized that *in situ* deprotection by cytosolic esterases might catalyze the transformation of **8f** (di-Cbz) to **8h** (free guanidine), the potent TrmD inhibitor, suggesting that **8f** might function as a more permeable prodrug.^37–40^

To test this hypothesis, we investigated whether carbamates **8f** and **8g** can be cleaved by total *S. aureus* lysates. A native *S. aureus* RN4220 lysate was prepared and incubated for 16 h with carbamates **8f** and **8g** (*c* = 400 μM), followed by LC/MS base-peak analysis of the lysate (Figure 7A). Interestingly, both carbamates **8f** and **8g** were readily cleaved in the presence of the lysate, yielding the production of hit compound **8h**, which could be detected in both lysates (Figure 7C). Both di-Boc and di-Cbz prodrugs appeared to be cleaved to a similar degree (∼30%) even at the very high concentration of 400 μM.

**Figure 7:**
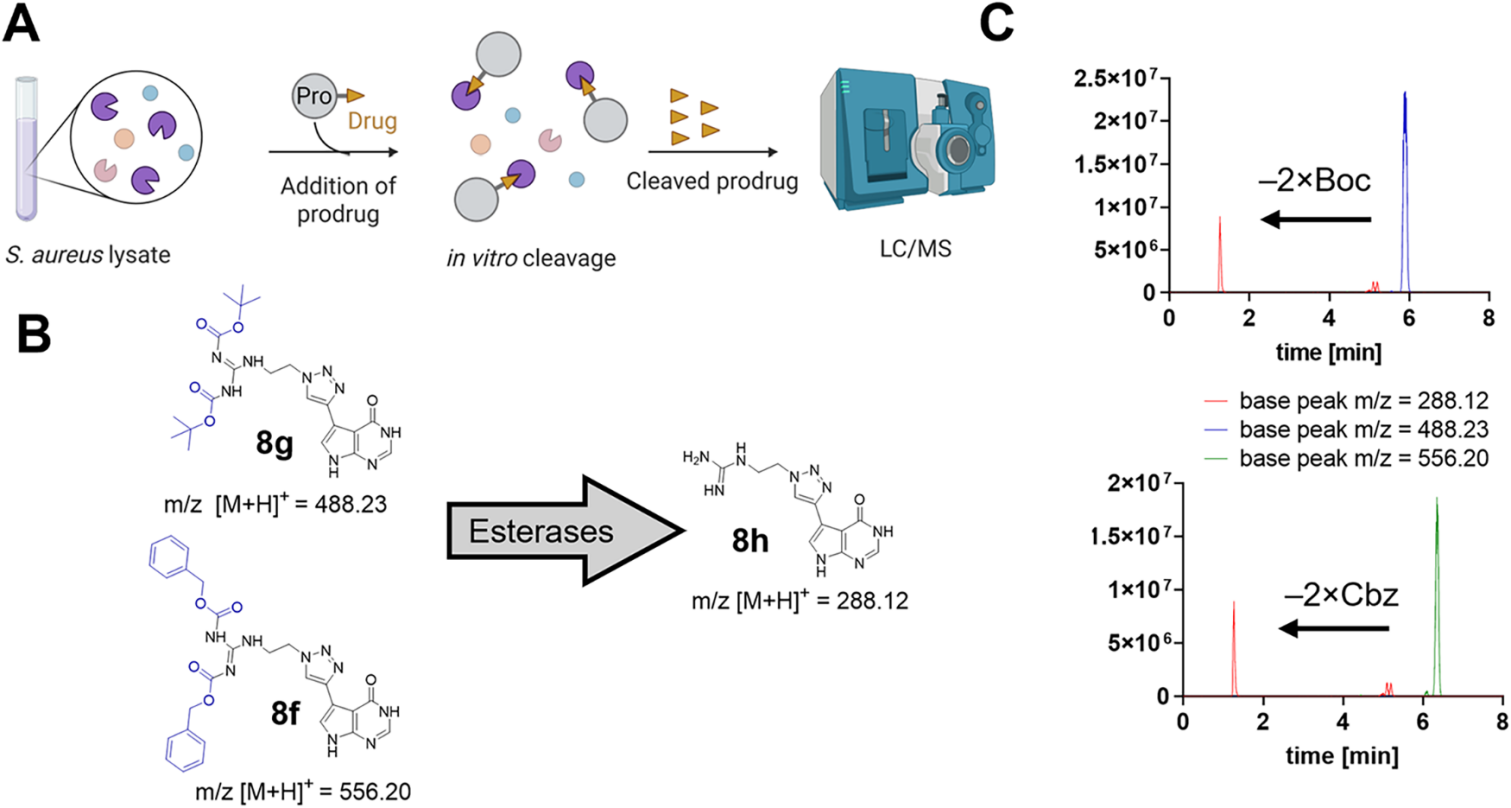
Carbamate-containing synthetic intermediates are readily cleaved by an *S. aureus* cell lysate. (A) Methodology of the conducted prodrug cleavage assay. (B) Structures and calculated masses of carbamates **8f**, **8g**, and for the deprotected hit compound **8h**. (C) LC/MS base-peak chromatograms of *S. aureus* lysates treated with **8f** or **8g** (400 μM) for 16 h. Detailed results of reference and base-peak chromatograms can be found in Figure S17.

In this context, single Boc- or Cbz-conjugated guanidines as intermediary deprotection products were not found by LC/MS analysis. Detailed results of reference and base-peak chromatograms can be found in Figure S17. Since carbamate **8g** (di-Boc) was found to be cleaved in the bacterial lysate, too, its lower antistaphylococcal potency in *S. aureus* experiments might be due to lower permeability compared to **8f** (di-Cbz). In summary, we were able to effectively reduce the growth of *S. aureus* cells with **8f**, which was not the primary end point of the nanoSAR optimization, but potentially acts as a *S. aureus* carbamate prodrug, which is metabolized by *S. aureus* cells to yield the actual inhibitor **8h** through cleavage of the di-Cbz carbamate.

Previous studies on TrmD inhibitors have also demonstrated that the incorporation of lipophilic moieties enhances antibacterial efficacy compared to analogous compounds lacking such features, which were not effective in the Gram-positive bacterium *S. aureus*.^14,16^ Thus, far, lipophilic tags of this Cbz-carbamate type have not been applied as prodrugs in contexts where the differences between IC_50_-values and MICs were mechanistically explainable. Our findings suggest that, through prodrug strategies, *S. aureus* TrmD can be more effectively targeted, demonstrating that TrmD can, in principle, be targeted with more permeable inhibitors.

To further validate our prodrug hypothesis *in vivo*, we next investigated the intracellular formation of the active TrmD inhibitor **8h** in growing *S. aureus* cells by LC/MS-based quantification. Bacterial cultures were treated with sublethal concentrations (4 μM) of compounds **8f**, **8g**, and **8h**, respectively, followed by cell harvesting and metabolite extraction. Remarkably, only treatment with the di-Cbz carbamate **8f** resulted in detectable intracellular levels of **8h**, while no formation of **8h** could be observed upon treatment with equimolar concentrations of **8g** or **8h** (Figure S18). This finding is in line with our prodrug hypothesis, demonstrating that **8f** undergoes enzymatic cleavage in *S. aureus* cells *in vivo* to release the active TrmD inhibitor **8h**. The absence of measurable intracellular **8h** upon direct treatment with **8h** or **8g** suggests limited uptake of these more polar species, further supporting that the improved antibacterial efficacy of **8f** originates from its enhanced permeability combined with intracellular prodrug activation.

### Crystal Structures of TrmD-Inhibitor Complexes

To date, no ligand-bound structure of *S. aureus* TrmD has been reported, with the PDB containing only one apo structure (PDB: 3KY7) of this enzyme.^41^ To analyze the binding mode of the compounds identified in the nanoSAR study and to guide future inhibitor optimization, we cocrystallized *S. aureus* TrmD with inhibitor **2** and the new TrmD inhibitor **8h.**

The cocrystal structure of **2** (Figure 8A) was solved at a resolution of 2.5 Å in the space group *P*4_3_32, containing one protein molecule per asymmetric unit. The structure exhibited a fold similar to that of the apo *S. aureus* TrmD structure (PDB: 3KY7) and other members of the SPOUT-class TrmD enzymes, as reflected by an overall C_α_ RMSD of 0.50 Å (Figure S19A). The N- and C-terminal domains of the monomer were resolved, but the highly flexible linker connecting the domains could not be resolved, similar to many published TrmD structures.^17,42–44^ For the cocrystallized complex, the N-terminal domain showed discrete electron density for inhibitor **2** (Figure 8A, Figure S19). Comparison with the cocrystal structure of inhibitor **2** bound to *H. influenzae* TrmD (PDB: 4MCC) highlights that the structures adopted a similar fold with an overall C_α_ RMSD of 1.44 Å (Figure S19B). Also, the conformation and ligand interactions of **2** were found to be similar for both *H. influenzae* and *S. aureus* enzymes (Figure 8B). An ionic interaction was formed between the primary amine of **2** and Glu132 in *S. aureus* (Glu116 in *H. influenzae*), a residue highly conserved among TrmD orthologues.^8^ The thienopyrimidone ring system displayed interactions with *S. aureus* Met149 (*H. influenzae* Ile133), Tyr152 (Tyr136), Leu154 (Leu138), and Pro105 (Pro89), within the conserved SAM pocket (Figure 8B).

**Figure 8:**
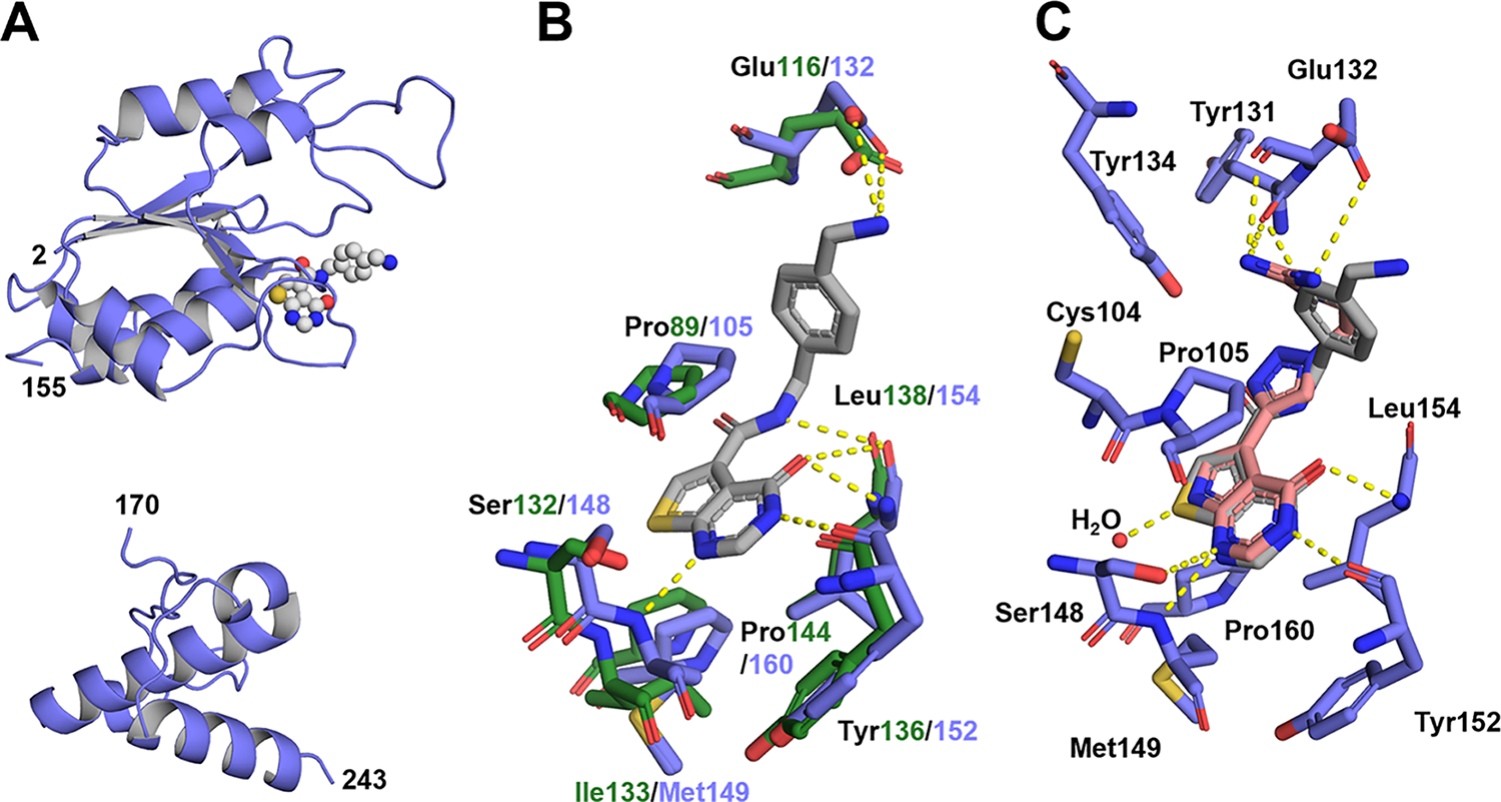
Crystal structures of compounds **2** and **8h** binding to *S. aureus* TrmD. (A) Structure of *S. aureus* TrmD bound to **2**. The protein is depicted as a violet-blue cartoon, and the ligand’s carbon atoms as gray spheres. (B) Compound **2** bound to *S. aureus* TrmD (PDB: 9SDV) overlaid with *H. influenzae* TrmD (PDB: 4MCC) binding pocket amino acids. (C) Overlay of **2** (PDB: 9SDV) and **8h** (PDB: 9SDW) in the *S. aureus* binding pocket. Ligands and amino acids are shown in stick representation; polar interactions are shown as yellow lines, and water as red spheres. The carbon atoms of **2** are shown in gray and those of compound **8h** in salmon. Interacting residues are displayed in violet-blue (*S. aureus*) or dark green (*H. influenzae*).

To analyze the differences in binding affinity of parent compound **2** between the *H. influenzae* and *S. aureus* enzymes, we used the HYDE scoring function in the SeeSAR toolkit to predict favorable and unfavorable contributions of individual ligand atoms to the overall binding energy (Figure S20). The computer-aided investigation revealed that the main difference stems from the amide group interactions, which contributed favorably to *H. influenzae* TrmD (HYDE: −3.8 kJ/mol), but unfavorably to the *S. aureus* enzyme (HYDE: +11.0 kJ/mol). This is in line with the observation that fluorescent tracer **3** (*K*_D_ = 23.3 μM), containing an amide moiety, showed lower *S. aureus* TrmD affinity to the target than tracer **4** (*K*_D_ = 659 nM), where the amide bond was omitted (Figure 2B). Additionally, the terminal amine of inhibitor **2** contributed favorably (HYDE: −3.0 kJ/mol) for the *H. influenzae* enzyme, whereas it resulted in a slightly unfavorable interaction for the *S. aureus* enzyme (HYDE: +0.3 kJ/mol). Also, these two crystallographic findings rationalize the results gleaned from the nanoSAR optimization: the transformation of amide to triazole and the transformation of benzylamine to guanidine were beneficial for *S. aureus* TrmD affinity due to their protein–ligand interactions.

To evaluate the binding interactions and conformation of hit compound **8h**, we cocrystallized this compound with the *S. aureus* TrmD (Figure 8C, Figure S21A). The structure was solved at a resolution of 2.6 Å in the space group *P*4_3_32. The fold compared to the apo structure (PDB: 3KY7) had an overall RMSD­(C_α_) of 0.51 Å (Figure S21B), indicating minimal changes in the conformation after ligand binding. For the cocrystallized complex, electron density for **8h** was visible in the SAM-binding N-terminal domain (Figure S21). A comprehensive schematic of ligand-protein interactions for compound **2** and hit compound **8h** was generated with LigPlot+ (Figure S22).^45^ In the ligand-binding pocket, the pyrrolopyrimidone ring of **8h** occupies a subpocket analogous to the thienopyrimidone ring system of compound **2** (Figure 8C). In both compounds, the pyrimidone *N* ^1^ nitrogen formed hydrogen bonds to the backbone of Met149; in **8h**, it additionally formed a hydrogen bond to the Ser148 side-chain hydroxyl. The pyrimidone *N* ^3^ nitrogen formed hydrogen bonds to the backbone of Tyr152 in both compounds. Likewise, the pyrimidone carbonyl oxygen interacts with the backbone of Leu154 in each compound. Unique to **8h**, the pyrrole nitrogen atom was found to interact with a water molecule inside the binding pocket, and the guanidine group of **8h** displayed ionic interactions with the side chain of Glu132, analogous to the primary amine of **2**. However, the orientation of the guanidine group differs from that of the amine, extending slightly deeper into the binding pocket and forming additional polar interactions with the backbone of Glu132 and Tyr131, rationalizing the improved affinity and selectivity for *S. aureus* TrmD.

To gain further insights into the selectivity and binding mode of parent compound **2** and compounds **8b–e,h** for the TrmD isoenzymes used in this study, molecular docking was conducted using the Glide algorithm.^46^ This analysis compared TrmD structures from *H. influenzae* (PDB: 4MCC), *E. coli* (PDB: 1P9P), and the *S. aureus* structure containing compound **8h** (PDB: 9SDW). Analysis of the docking scores (Table S3) was highly consistent with the ITC and FP assay results: parent compound **2** is predicted with higher binding affinity than compounds **8b–e,h** for *H. influenzae* and *E. coli* isoenzymes, while for *S. aureus* TrmD the triazole-containing **8b–e,h** is preferred over compound **2.** Comparing the predicted poses, in general for compounds **8b–e,h**, we found either ionic interactions between Glu132 and the basic moieties (guanidine and amine) of the inhibitor or hydrophobic interactions between the nonpolar aliphatic and aromatic proportions of the ligand with the binding pocket (Figure S24). The relative affinity differences between TrmD isoenzymes, aside from the scaffold exchange from thienopyrimidone to pyrrolopyrimidone, likely arise from linker-dependent differences in positioning (azide vs amide), which modulate interactions of the azide/amide moiety and the placement of azide-connected substituents within the pocket. This is reflected in both docking scores (Table S3) and predicted poses (Figure S24).

Notably, Tyr134 in *S. aureus* is positioned close to the substituents of the compounds; for example, the side chain oxygen lies only 5.2 Å from the guanidine group carbon of compound **8h**. As this residue is replaced by isoleucine in the *H. influenzae* and *E. coli* isoenzymes, selectivity might be enhanced by targeting this position. In addition, Cys104, which is not conserved in TrmD isoenzymes, is located near the ligands, and addressing this residue with a covalent warhead could further increase selectivity.^8^

## Conclusion

In this work, we employed a nanoSAR triazole synthesis strategy coupled with a direct-to-biology FP read-out assay to develop affine and selective *S. aureus* TrmD inhibitors. This screening pipeline identified five hit compounds, which exhibited IC_50_-values and *K*_D_-values in the nanomolar to low micromolar range. In line with previous TrmD drug development campaigns, the identified inhibitors engage the adenine binding subpocket, and the substituents identified by nanoSAR screening expand toward the conserved acidic residue containing TrmD loop.^14,16,18–20^ Ionic interactions with Glu132 (or orthologous residues) are a frequently identified interaction fingerprint of previous TrmD inhibitors.^14,16,18–20^ Yet, the D2B optimization presented here was able to identify not only potent but also selective *S. aureus* TrmD inhibitors. Yet, during cellular screening, none of these initial inhibitors showed antibacterial activity against *S. aureus*, a property several disclosed TrmD inhibitors with diverse chemotypes share.^14,16,18–20^ Surprisingly, one of the synthetic intermediates (**8f**, di-Cbz-masked form of **8h**) showed antimicrobial effects at low micromolar concentrations. We hypothesized that ligand permeability was preventing active inhibitors from engaging cytosolic TrmD target localization, an assumption that has been made in previous TrmD drug discovery campaigns.^14,16,18–20^ In a prodrug-lysate assay, we observed that compound **8f** is indeed readily cleaved to form the active inhibitor **8h**, thereby confirming our hypothesis that **8f** might function as a prodrug of **8h**. This concept of Cbz-based prodrug might have broader applicability beyond the presented TrmD inhibitor type, both for other TrmD inhibitors incorporating amine/amidine chemotype and for a broader range of antibiotics.

Subsequently, X-ray crystallography of hit **8h** revealed its binding mode within the SAM-binding pocket of *S. aureus* TrmD, providing structural insights into key interactions. These findings will enable structure-guided optimization of this scaffold to further enhance both the affinity and selectivity of future inhibitors. In this regard, molecular modeling revealed two key targets for selectively increasing the affinity to *S. aureus* TrmD compared to other isoenzymes: Cys104 and Tyr134, which are located in close proximity to the bound ligand **8h** (PDB: 8SDW). These two residues are a unique feature of *S. aureus* TrmD and do not exist in most other TrmD isoenzymes; thus, it is conceivable that future campaigns could address these residues, for example, by covalent warheads.^8^

## Experimental Procedures

### Protein Preparation and Purification

#### Recombinant Expression of *H. influenzae* TrmD

TrmD of *H. influenzae* was expressed as described previously.^31^ In short, the pET28b­(+) plasmid encoding for TrmD of *H. influenzae*, kindly provided by Se Won Suh (Addgene ID: 12665), was used to transform competent BL21 *E. coli* cells. The cells were grown in LB medium containing 50 μg/mL kanamycin at 37 °C and 160 rpm until they reached an OD_600_ of ∼0.7. After reducing the temperature to 16 °C, 0.5 mM isopropyl-β-d-1-thiogalactopyranoside (IPTG) was added to induce overexpression, which was maintained overnight. The cells were harvested by centrifugation and suspended in lysis buffer (20 mM HEPES pH 7.5, 300 mM NaCl, 0.5 mM TCEP, 5% glycerol, 0.1% polysorbate-20). Lysozyme and DNase I were added, and after a 30 min incubation on ice, the cells were lysed by sonication. Cell debris was removed by centrifugation. The supernatant was applied to a HisTrap HP column for affinity purification, pre-equilibrated in binding buffer (20 mM HEPES pH 7.5, 300 mM NaCl, 5 mM imidazole, 0.5 mM TCEP, 5% glycerol, 0.1% polysorbate-20) and washed with several column volumes of binding buffer. Afterward, the protein was eluted in elution buffer (20 mM HEPES pH 7.5, 300 mM NaCl, 500 mM imidazole, 0.5 mM TCEP, 5% glycerol, 0.1% polysorbate-20). Finally, *H. influenzae* TrmD was purified via size-exclusion chromatography (SEC), on a Superdex 16/600 75 pg SEC column equilibrated with SEC buffer (25 mM HEPES pH 7.5, 300 mM NaCl, 1 mM TCEP, 10% glycerol, 0.1% polysorbate-20). The enzyme was flash-frozen in liquid nitrogen and stored at −80 °C until further use.

#### Recombinant Expression of *S. aureus* TrmD

Expression and purification were performed similarly to *H. influenzae* TrmD, as described above. The pET28b­(+) plasmid encoding for TrmD of *S. aureus*, kindly provided by Angelika Gründling, was used to transform competent BL21 *E. coli* cells. The cells were grown in LB medium containing 50 μg/mL kanamycin at 37 °C and 160 rpm until they reached an OD_600_ of ∼0.7. After reducing the temperature to 16 °C, 0.5 mM IPTG was added to induce overexpression, which was maintained overnight. The cells were harvested by centrifugation and suspended in lysis buffer (20 mM HEPES pH 7.5, 300 mM NaCl, 0.5 mM TCEP, 5% glycerol, 0.1% polysorbate-20). Lysozyme and DNase I were added, and after a 30 min incubation on ice, the cells were lysed by sonication. Cell debris was removed by centrifugation. The supernatant was applied to a HisTrap HP column for affinity purification, pre-equilibrated in binding buffer (20 mM HEPES pH 7.5, 300 mM NaCl, 5 mM imidazole, 0.5 mM TCEP, 5% glycerol, 0.1% polysorbate-20) and washed with several column volumes of binding buffer. Afterward, the protein was eluted in elution buffer (20 mM HEPES pH 7.5, 300 mM NaCl, 500 mM imidazole, 0.5 mM TCEP, 5% glycerol, 0.1% polysorbate-20). Finally, *S. aureus* TrmD was purified on a Superdex 16/600 75 pg SEC column equilibrated with SEC buffer (40 mM Tris pH 7.5, 100 mM NaCl, 10 mM MgCl_2_, 5% Glycerol). The enzyme was flash-frozen in liquid nitrogen and stored at −80 °C until further use.

#### Recombinant Expression of *E. coli* TrmD

Bacterial strains carrying the expression pCA24N plasmid of *E. coli* TrmD were received from the Microbial Physiology Laboratory of the National Institute of Genetics in Japan (National BioResource Project). Expression and purification were performed as described in the original publication with slight adaptations.^47^ The cells were grown in LB medium containing 30 μg/mL chloramphenicol at 37 °C and 160 rpm. After the cells reached an OD_600_ of ∼0.7, 1 mM IPTG was added to induce overexpression. After a 2 h incubation, the cells were harvested by centrifugation and suspended in binding buffer (50 mM sodium phosphate pH 7.5, 150 mM NaCl, 10 mM imidazole, 0.1% Tween 20). Lysozyme was added, and after a 30 min incubation on ice, the cells were lysed by sonication. Cell debris was removed by centrifugation. The supernatant was applied to a HisTrap HP column for affinity purification, pre-equilibrated in binding buffer, and washed with several column volumes of binding buffer. Afterward, the protein was eluted in elution buffer (50 mM sodium phosphate pH 7.5, 150 mM NaCl, 500 mM imidazole, 0.1% Tween 20). Finally, *E. coli* TrmD was mixed 1:5 with storage buffer (50 mM sodium phosphate pH 8.0, 300 mM NaCl, 2 mM DTT, 1 mM EDTA, 0.1% Tween 20, 60% Glycerol). The enzyme was flash-frozen in liquid nitrogen and stored at −20 °C until further use.

#### Recombinant Expression of Human Trm5

A pET-28a­(+) plasmid encoding a truncated construct of Trm5 (aa 66–475), with flexible termini omitted, carrying the putative RNA and SAH binding sites, was used to transform competent *E. coli* Rosetta2 (DE3) cells. The cells were grown at 37 °C and 160 rpm in LB medium containing 50 μg/mL kanamycin and 25 μg/mL chloramphenicol. After reaching an OD_600_ of ∼0.7. The temperature was reduced to 18 °C. 0.2 mM IPTG was added to induce overexpression, and the cells were incubated overnight. After harvesting the bacteria by centrifugation, these were suspended in lysis buffer (50 mM Tris HCl, 500 mM sodium chloride, 1 mM β-mercaptoethanol, 0.5 mM PMSF, 5% glycerol, pH 7.5). A protease inhibitor tablet (cOmplete) was added, and the cells were lysed by sonication. After removing the cell debris by centrifugation, the supernatant was applied to a HisTrap FF column for affinity purification, pre-equilibrated in binding buffer (20 mM Tris HCl, 500 mM sodium chloride, 1 mM β-mercaptoethanol, 0.5 mM PMSF, 25 mM imidazole, 5% glycerol, pH 7.5). After washing the column with several column volumes of binding buffer, the protein was eluted in elution buffer (20 mM Tris HCl, 500 mM sodium chloride, 1 mM β-mercaptoethanol, 0.5 mM PMSF, 250 mM imidazole, 5% glycerol, pH 7.5). As a final purification step, Trm5 was purified on a Superdex 16/600 75 pg SEC column equilibrated with SEC buffer (20 mM HEPES, 250 mM sodium chloride, 10 mM β-mercaptoethanol, 10% glycerol, pH 7.5). The enzyme was flash-frozen in liquid nitrogen and stored at −80 °C until further use.

### Fluorescence Polarization (FP) Assays

Fluorescence polarization (FP) assays were performed as follows. The assays for *H. influenzae* TrmD have been described previously.^31^ All assays were conducted in technical triplicate using black 96-well half area plates. For the FP assays, a tracer (20 nM Cy5TPD^31^ for *E. coli* and *H. influenzae* TrmD, 10 nM tracer **4** for *S. aureus* TrmD) was mixed with the respective enzyme (2 μM *E. coli* TrmD, 2 μM *H. influenzae* TrmD, 500 nM *S. aureus* TrmD) in buffer (*E. coli* and *H. influenzae* TrmD: 50 mM HEPES, 500 mM NaCl, 5% Glycerol, pH 7.5, *S. aureus* TrmD: 40 mM Tris HCl, 100 mM NaCl, 10 mM MgCl_2_, 5% Glycerol, pH 7.5). The compounds were added in varying concentrations for *K*_D_-value determination. For *K*_D_-value determination of tracers, the enzyme concentration was varied, and no inhibitory compounds were added (DMSO < 0.5% for TrmDs). The Z-factor for tracer **4** was determined by measuring 48 replicates of the free tracer (positive controls, PC) and 48 replicates of a mixture of enzyme and tracer (negative controls, NC). The *Z*-factor is defined in terms of the means (μ) and standard deviations (σ) of the positive and negative controls and calculated as *Z* = 1–(3*­(σ_nc_ + σ_pc_)/(μ_nc_–μ_pc_)). Fluorescence polarization was measured using a Spark 10 M plate reader (Tecan) equipped with polarization filters and a monochromator setup (λ_ex_ = 635 nm, λ_em_ = 680 nm). Polarization values (mP) were determined from polarization-specific parallel and orthogonal fluorescence intensities. *K*_D_-values were calculated in GraphPad Prism 8.0.1 using the four-parameter Hill equation: *y*(% tracer bound) = bottom + ([ligand]­slope) × (top–bottom)/([ligand]­slope + *K*_D_slope).

### Aptamer-Based MTase Enzyme Inhibition Assay

For the determination of *S. aureus* TrmD IC_50_-values, a split aptamer assay according to Nidoieva et al. was employed.^33^ The assay was performed in 40 mM Tris HCl, 100 mM NaCl, 10 mM MgCl_2_, 5% Glycerol, pH 7.5. Enzyme reaction mixtures contained synthetic tRNA^LeuCAG^ substrate (synthetic tRNA^Leu^: 5′-GCGAAGGUGGCGGAAUUGGUAGACGCGCUAGCUUCAGGUGUUAGUGUCCUUACGGACGUGGGGGUUCAAGUCCCCCCCCUCGCACCA-3′, Genscript, final concentration: 2 μM), *S. aureus* TrmD (final concentration: 100 nM), SAM (New England Biolabs, final concentration: 2 μM), and the test compounds in varying concentrations (3% DMSO, final volume 10 μL). Pure DMSO was used as a mock treatment reaction control. The assay was carried out in triplicate in black 384-well plates (Greiner). Enzyme reactions were quenched by the addition of 1 μL SDS (3%) after an incubation time of 60 min at room temperature. Subsequently, the aptamer detection mix described in Nidoieva et al. was added to the reactions, and samples were measured using a Tecan Spark 10 M plate reader equipped with a monochromator setup (λ_ex_ = 485 nm; λ_em_ = 600 nm) exported for statistical analysis and plotting in GraphPad Prism 8.0.1.

### ^3^H-based MTase Enzyme Inhibition Assay

^3^H-based inhibition assays for TrmD *S. aureus* were performed in 50 mM HEPES, 5 mM MgCl_2_, 80 mM NaCl, 0.005% Tween-20, 1 mM DTT, pH 7.5 buffer; and for Trm5 ^48^ in 100 mM Tris-HCl, 0.1 mM EDTA, 6 mM MgCl_2_, 100 mM KCl, 2 mM DTT, pH 7.4. The reaction mixture contained for *S. aureus* TrmD: 1 μM synthetic tRNA^Leu^ (Genscript, see above) and 100 nM *S. aureus* TrmD; For human Trm5:10 μM synthetic tRNA^Leu^ and 1.5 μM Trm5. Compounds were screened for *S. aureus* TrmD at 1 μM and for Trm5 at 30 μM. The enzymatic reactions were started by the addition of SAM as a mixture of ^3^H-SAM (Hartmann Analytics, *S. aureus* TrmD: 0.5 μM, Trm5: 2 μM) and cold SAM (New England Biolabs, *S. aureus* TrmD: 0.5 μM, Trm5: 98 μM) to a final activity of 0.04 μCi μL^–1^ (TrmD) or 0.16 μCi μL^–1^ (Trm5). The reaction mixtures were incubated in duplicate for 60 min (TrmD)/30 min (Trm5) at 37 °C. As a negative control, the reaction mixture without the enzyme was used, and as a positive control, the reaction mixture without the test compound was used. After the enzyme incubation time, aliquots of 8 μL were taken from the reaction mixture and spotted on Whatman glass microfiber filters (GF/C, 25 mm). The tRNA was precipitated on the filters with 5% ice-cold TCA for 15 min. The filters were washed twice with 5% TCA at room temperature for 20 and 10 min, and once in EtOH for 10 min. After drying, the filters were transferred into scintillation vials with 3 mL of PerkinElmer Gold MV liquid scintillation cocktail. Scintillation was measured with a scintillation counter (TriCarb Liquid Scintillation Analyzer 4810TR; measurement time of 1 min).

### Isothermal Titration Calorimetry (ITC)

Experiments were conducted in the *S. aureus* TrmD SEC buffer (40 mM Tris, pH 7.5, 100 mM NaCl, 10 mM MgCl_2_, 5% glycerol) for the *S. aureus* enzyme. The *H. influenzae* enzyme was buffer exchanged into its ITC buffer (50 mM HEPES, pH 7.5; 500 mM NaCl; 5% glycerol) using Amicon Ultra-15 centrifugal filter units (Merck) prior to use. Compound **1** (BLD Pharmatech) and compound **2** were dissolved in DMSO to a final concentration of 20.0 and 11.6 mM, respectively, and diluted in ITC buffer to give ligand solutions at 1 and 0.58 mM, respectively. Due to a low enthalpy signal, a low c-value titration was performed for compound **1**. In this type of experiment, the ligand is typically added at high concentration and in large excess relative to its binding partner, driving more binding events per injection. This causes the equivalence point to be reached after only a few injections and leads to the loss of the characteristic sigmoidal binding curve. TrmD was diluted to a final concentration of 50 μM, and DMSO was added to a final concentration of 5% to match the buffers. ITC measurements were performed using a MicroCal PEAQ-ITC Automated workstation (Malvern Panalytical) equipped with a 200 μL Hastelloy cell and a 40 μL injection syringe. The experiments were conducted in duplicates at 25 °C with a stirring speed of 750 rpm and a reference power of 42 μW. Each titration consisted of 19 injections of 2 μL compound solution, delivered into the cell containing 50 μM TrmD at 0.5 μL/s, with 150 s between injections. Control experiments were performed by titrating ligands into the ITC buffer. Data were analyzed and fitted using the MicroCal PEAQ-ITC Analysis Software 1.21 and plotted in GraphPad Prism 8.0.1.

### Bacterial Growth Inhibition Assays

Compounds were dissolved in DMSO to a final concentration of 20 mM. To determine the compound’s activity against different bacteria, a bacterial culture (*E. coli* ATCC10536, *S. aureus* RN4220) was set up to an optical density at 600 nm (OD_600_) of 0.05 from an overnight culture in Mueller-Hinton broth (Carl Roth). The compound was added in different concentrations ranging from 400 μM to 1 μM. DMSO concentration was constantly adjusted to be 1% in the final culture. Cultures of 200 μL were incubated for 24 h at 37 °C in flat-bottom 96-well polystyrene culture plates (Greiner) using a BioTek Synergy H1 plate reader. Plates were incubated under constant shaking, and absorbance at 600 nm was measured every 20 min. The experiment was performed in three biological replicates with three technical replicates each. For the statistics, two-way ANOVA with Dunnett’s multiple comparisons test was used because results were affected by two factors (time and compound concentration). As all results were compared to an untreated sample, Dunnett’s test was used to correct for multiple comparisons using a confidence interval of 95%. Time points 360 min (6 h), 720 min (12 h), and 1440 min (24 h) were chosen and analyzed using GraphPad PRISM (10.4.2).

To determine the minimal bactericidal concentration of the substances, bacterial cultures were prepared as in the bacterial growth inhibition assay but incubated statically at 37 °C. A serial dilution of the initial culture was made and plated on a TSB agar plate to determine the CFU/mL of the original culture. After 24 h, the cultures were diluted again and plated. Bacterial colonies were counted, and the CFU/mL was calculated. The experiment was performed in *n* = 3 biological replicates. Data evaluation was done using GraphPad Prism. One-way ANOVA with Dunnett’s multiple comparisons test was used for statistical analysis, and all samples were compared to the starting CFU/mL.

### Cell Titer Glo Cell Viability Assay

Cell viability assays were conducted with HEK293 cells (ATCC) to investigate the compounds’ mammalian cytotoxicity. Cells were cultured according to standard protocols (DMEM, 10% FBS, 1× Penicillin/Streptomycin, 37 °C, 5% CO_2_). Cell viability was assessed using the Promega CellTiterGlo assay kit. Briefly, 1000 cells were seeded in white half a rea 96-well plates (Greiner) and incubated for 48 h with inhibitors from DMSO stocks (final: 0.1% DMSO) under standard cultivation conditions. Then, the cell titer reagent was added to each well according to the manufacturer’s protocols, and luminescence was measured using a Tecan Spark 10 M plate reader.

### Bacterial Lysate Carbamate Cleavage Assays

An overnight culture of *S. aureus* RN4220 was set up in TSB medium (BD Bacto Tryptic Soy Broth). The next day, a 150 mL day culture was started at an initial OD_600_ of 0.1. The culture was incubated at 200 rpm shaking at 37 °C. The bacteria were harvested after 3 h and 40 min (OD_600_ = 3.30). A 1 mL aliquot was retained to determine cell number. 150 mL of the culture was centrifuged at 4000 rpm for 10 min at 4 °C. The resulting pellet was resuspended in 10 mL of JB Lysis Buffer (0.1% Triton X-100, 20 mM Tris-HCl, 150 mM KCl, 1 mM MgCl_2_, 1 mM DTT, and protease inhibitors) and transferred to FastPrep tubes. Cells were mechanically lysed (2 × 30 s, 6.5 m/s) and cooled for 5 min on ice. The samples were centrifuged, and the supernatant was transferred to a new tube. Subsequently, it was centrifuged again at 5000 rpm for 30 min at 4 °C. The pellet was washed three times with 1× PBS, reducing the volume to 1 mL. Protein concentration was determined using the Pierce BCA Protein Assay Kit (Thermo Scientific). The final sample was frozen in liquid nitrogen and stored at −80 °C. 100 μL of this lysate was incubated with **8f**–**h** (final concentration 400 μM, 2% DMSO) for 16 h at 30 °C. Afterward, 100 μL acetonitrile was added, and the samples were passed through a syringe filter. Ten μL of the samples were injected during LC/MS (Agilent 1100 system, LC/MSD trap) using an Agilent Poroshell 120 EC-C18 150 × 2.10 mm 4 μm column; mobile phase (gradient: acetonitrile/H_2_O, 0.1% formic acid). The LC/MS chromatograms and their corresponding mass spectra (base-peak) were analyzed using MestReNova (12.0.4) for prodrug deprotection products.

### *S. aureus* Inhibitor Uptake Assay

An overnight culture of *S. aureus* RN4220 was set up in Müller-Hinton medium. The next day, a 100 mL day culture with 4 μM of compound (**8f**, **8g**, **8h**, or DMSO) was started at an initial OD_600_ of 0.05. The culture was incubated at 200 rpm shaking at 37 °C. The total bacterial culture was harvested after reaching an OD_600_ of 0.3 by centrifugation at 4,000 rpm for 10 min at 4 °C. The resulting pellet was washed twice with 1× PBS, then resuspended in 1 mL of JB Lysis Buffer (0.1% Triton X-100, 20 mM Tris-HCl, 150 mM KCl, 1 mM MgCl_2_, 1 mM DTT, and protease inhibitors) and transferred to Lysis matrix E tubes. Cells were mechanically lysed by Fast prep (2 × 30 s, 6.5 m/s) and cooled for 5 min on ice. The samples were centrifuged, and the supernatants were transferred to a new tube. Subsequently, they were centrifuged again at 5000 rpm for 30 min at 4 °C. The resulting pellet was washed twice with 1× PBS, reducing the volume to 100 μL, while the resulting supernatant was cleared once more by centrifugation. The samples were frozen in liquid nitrogen and stored at −80 °C. The prepared lysates were loaded on a solid-phase extraction cartridge (C18 Chromabond, Macherey-Nagel) and eluted with 30% ACN in water (1 mL). The collected eluate was lyophilized and resolved in 100 μL ACN/water. 10 μL of the samples were injected during LC/MS (Waters Alliance 2695 coupled to a Waters QDa mass-sensitive detector) using an Agilent Poroshell 120 EC-C18 150 × 2.10 mm 4 μm column; mobile phase (gradient: acetonitrile/H_2_O, 0.1% formic acid). The LC/MS chromatograms and their corresponding mass spectra (base-peak) were analyzed using MestReNova (12.0.4) for prodrug deprotection products.

### Crystallization, Data Collection, Model Building, and Refinement

Co-crystals of the *S. aureus* TrmD protein and compounds **2** or **8h** were obtained at 20 °C with a hanging drop setup. The protein (271 μM) was incubated with compounds in 7-fold molar excess (2 mM final concentration) for 30 min on ice before crystallization. The reservoir solution contained 1.4 M Na Malonate pH 7.4, 0.1 M Bis-Tris Propane.^42^ Crystallization drops consisted of 1 μL reservoir solution and 1 μL of the preincubated protein and compound. Cubic crystals grew within 2 weeks. Crystals were frozen in liquid nitrogen using 25% glycerol as the cryo-protectant. Diffraction data were collected at the Diamond Light Source Beamline I03 (Oxfordshire, U.K.). All data sets were processed with XDS^49^ and scaled with AIMLESS within the CCP4 ^50^ cloud.^50–52^ The resulting data sets had a maximum resolution of 2.50 Å (Structure with compound **2**) or 2.60 Å (Structure with compound **8h**). The crystals belong to space group *P*4_3_32 with one TrmD molecule per asymmetric unit. The structures were solved by molecular replacement with PHASER using Apo-TrmD (PDB: 3KY7) as the search model.^53,54^ The compounds were parametrized from SMILES using phenix.elbow.^55^ Model building and refinement were performed with Coot, Refmac, and phenix.refine.^50,56–58^ The quality of the resulting structural models was assessed using MolProbity.^59,60^ Due to the high flexibility of the loop between amino acids Val175 and Ser188, this region was not modeled. Additionally, some amino acids with flexible side chains and insufficient electron density to fit the side chain were truncated to alanine residues. In the structure with compound **2**, residues Leu35, Asn36, Lys41, Arg42, Gln44, Lys48, Gln50, Lys63, Glu90, Glu193, and Lys204 were truncated. In the structure with compound **8h**, residues Asn36, Lys41, Arg42, Lys48, and His64 were truncated. The crystallographic data are summarized in Tables S1 and S2. Coordinates and structure factors are deposited at the Protein Data Bank (PDB) under accession codes: 9SDV and 9SDW.

### Molecular Modeling

For molecular docking of compounds **2** and **8b–e,h**, a Glide docking protocol was conducted within the Schrödinger Maestro 2020.04 worksuite.^46^ The protein structures 4MCC (*H. influenzae* TrmD) and 1P9P (*E. coli* TrmD) were downloaded from the PDB, and 9SDW was used for docking into the *S. aureus* isoenzyme. Receptor preparation was performed with the automated binding site, protonation, and energy minimization routine within Maestro “Protein preparation” and “Receptor grid generation”. Energy minimization of ligands was conducted using the “LigPrep” routine. The docking protocol was performed under default parameters with standard precision settings. Ligand binding poses were visualized using PyMol.

### General Synthesis Procedures

All reagents and solvents were of analytical grade quality and purchased from standard commercial suppliers. Chemicals were used without further purification unless otherwise noted. ^1^H and ^13^C spectra were recorded on Bruker Fourier 300 MHz, Bruker Avance Neo 400 MHz, and Bruker Avance III 600 MHz spectrometers. Chemical shifts δ are given in parts per million (ppm) using residual proton peaks of the solvent as the internal standard. HPLC and mass spectra were obtained by LC/MS, consisting of an 1100 series HPLC system from Agilent with an Agilent Poroshell 120 EC-C18 150 × 2.10 mm, 4 μm column. The detection wavelengths were dependent on the specific compound absorbance characteristics: 210 and 254 nm. The MTase tracers (**3** and **4**) were analyzed for their purity at 650 nm (Cy5). The molecular mass of all compounds and intermediates was analyzed by an Agilent 1100 series LC/MSD trap with electron spray ionization (ESI) in positive mode. Preparative column chromatography was performed with silica gel (0.06–0.02 mm) obtained from Macherey-Nagel or prepacked Biotage reverse-phase silica columns for flash chromatography (Biotage Isolera One) eluting with ACN/H_2_O. Preparative HPLC was performed on an Agilent 1290 system with an MZ-Aqua Perfect C18 7 μm column. All compounds are >95% pure by HPLC analysis.

### Protocol for the Nanomole-Scale Synthesis

A commercial azide diversity library (Enamine) containing 320 azides at a concentration of 100 mM in DMSO in a 96-well tube rack was employed for this nanoSAR study. For the reaction of the azides with alkyne **7** via CuAAC, a master mix was prepared. The master mix contained for one 96-well plate: 600 μL of a 33.33 mM sodium ascorbate solution dissolved in Milli-Q water, 200 μL of a 12.52 mM TBTA solution dissolved in DMSO, 500 μL of a 20 mM alkyne **7** solution dissolved in DMSO, 600 μL of a 4.81 mM CuSO_4_×5 H_2_O solution dissolved in Milli-Q water and 400 μL of pure DMSO. The solvents were degassed with argon to avoid oxidation of the copper­(I) during the reaction. To each well of a 96-well PCR plate (Applied Biosystems MicroAmp) were added 23 μL of the master mix and 1 μL of the 100 mM azide stock. The final concentrations in the Mastermix were: 8.7 mM sodium ascorbate (8.3 mM in reaction well), 1.1 mM TBTA (1.0 mM in reaction well), 4.3 mM **7** (4.1 mM in reaction well), 1.3 mM CuSO_4_×5 H_2_O (1.2 mM in reaction well). The 96-well PCR plate was purged with argon, and the implementation of the experiments was conducted under an argon atmosphere. The reactions were mixed, centrifuged, and sealed airtight. The plate was allowed to incubate at room temperature for 16 h before the formed triazoles were investigated via FP-assays.

### General Procedure for Preparative CuAAC

Compound **7** (2–5 mg, 0.013–0.031 mmol, 1.0 equiv), the respective azide (0.015–0.037 mmol, 1.2 equiv), TBTA (2–5 mg, 0.004–0.009 mmol, 0.3 equiv), copper­(II)­sulfate pentahydrate (1–2 mg, 0.004–0.009 mmol, 0.3 equiv), and sodium ascorbate (5–12 mg, 0.026–0.062 mmol, 2.0 equiv) were dissolved in water (500 μL) and DMSO (1.0 mL). The reaction was stirred for 16 h at room temperature under an argon atmosphere before the solvents were removed *in vacuo*. Afterward, the crude product was purified via reverse-phase flash chromatography.

#### Compound **2**: *N*-(4-(Aminomethyl)­benzyl)-4-oxo-3,4-dihydrothieno­[2,3-*d*]­pyrimidine-5-carboxamide

4-Oxo-3,4-dihydrothieno­[2,3-*d*]­pyrimidine-5-carboxylic acid (22 mg, 0.110 mmol, 1.0 equiv) and *tert*-butyl (4-(aminomethyl)­benzyl)­carbamate (26 mg, 0.110 mmol, 1.0 equiv) were mixed with HATU (42 mg, 0.110 mmol, 1.0 equiv) and DIPEA (60 μL, 0.343 mol, 3.0 equiv) in 4 mL anhydrous DMF. The mixture was stirred for 16 h at room temperature before the organic solvent was removed *in vacuo*. The residue was dissolved in DCM (1 mL) and TFA (1 mL) and stirred for 30 min at room temperature. The crude product was purified via flash chromatography. Yield: 17 mg, quant. ^1^H NMR (300 MHz, MeO*D*) δ [ppm] = 8.40 (s, 1H), 8.18 (s, 1H), 7.52–7.43 (m, 4H), 4.66 (d, *J* = 2.3 Hz, 2H), 4.13 (s, 2H). ^13^C NMR (75 MHz, MeOD) δ [ppm] = 168.9, 163.1, 161.2, 146.7, 141.0, 133.4, 132.8, 130.2, 129.4, 121.0, 44.1. LC/MS: *m*/*z* calculated for C_15_H_14_N_4_O_2_S [M + H]^+^: 315.1, found: 315.0.

#### Compound **3**: 3,3-Dimethyl-1-(6-oxo-6-((3-(5-((4-oxo-3,4-dihydrothieno­[2,3-*d*]­pyrimidine-5-carboxamido)­methyl)-1*H*-1,2,3-triazol-1-yl)­propyl)­amino)­hexyl)-2-((1*E*,3*E*)-5-((*E*)-1,3,3-trimethylindolin-2-ylidene)­penta-1,3-dien-1-yl)-3*H*-indol-1-ium trifluoroacetate

The compound was synthesized according to the general procedure for preparative click reactions with alkyne **10** (0.63 mg, 0.0027 mmol, 1.5 equiv) and Cy5-azide (1.1 mg, 0.0018 mmol, 1.0 equiv). Yield: 1.5 mg, quant. LC/MS: *m*/*z* calculated for C_45_H_52_N_9_O_3_S^+^ [M + H]^2+^: 399.7, found: 399.6. Purity (HPLC): 98%.

#### Compound **4**: 3,3-Dimethyl-1-(6-oxo-6-((3-(4-(4-oxo-4,7-dihydro-3*H*-pyrrolo­[2,3-*d*]­pyrimidin-5-yl)-1*H*-1,2,3-triazol-1-yl)­propyl)­amino)­hexyl)-2-((1*E*,3*E*)-5-((*E*)-1,3,3-trimethylindolin-2-ylidene)­penta-1,3-dien-1-yl)-3*H*-indol-1-ium trifluoroacetate

The compound was synthesized according to the general procedure for preparative click reactions with alkyne **7** (0.19 mg, 0.0016 mmol, 1.3 equiv) and Cy5-azide (0.7 mg, 0.0012 mmol, 1.0 equiv). LC/MS: *m*/*z* calculated for C_43_H_50_N_9_O_2_^+^ [M + H]^2+^: 362.7, found: 362.7. Purity (HPLC): 97%.

#### Compound **6**: 5-((Trimethylsilyl)­ethynyl)-3,7-dihydro-4*H*-pyrrolo­[2,3-*d*]­pyrimidin-4-one

To a minimum volume flask was added 5-iodo-3,7-dihydro-4*H*-pyrrolo­[2,3-*d*]­pyrimidin-4-one (232 mg, 0.890 mmol, 1.0 equiv), CuI (34 mg, 0.178 mmol, 0.2 equiv), TMS-acetylene (190 μL, 1.832 mmol, 2.0 equiv), Pd­(PPh_3_)_4_ (54 mg, 0.046 mmol, 0.05 equiv), THF (5.0 mL), DMF (2.0 mL), and triethylamine (70 μL). The reaction was stirred for 16 h at room temperature. Subsequently, the solvent was removed *in vacuo*, and the residue was dissolved in DCM (10 mL). The solution was washed with water (3 × 10 mL), and the solvent was removed. The crude product was purified via reverse-phase flash chromatography. Yield: 60 mg, 29%. ^1^H NMR (300 MHz, DMSO-*d*_6_) δ [ppm] = 12.17 (s, 1H), 11.86 (s, 1H), 7.84 (s, 1H), 7.39 (d, *J* = 2.6 Hz, 1H), 0.20 (s, 9H). ^13^C NMR (75 MHz, DMSO) δ [ppm] = 157.5, 147.8, 144.6, 126.4, 107.2, 99.9, 98.4, 93.6, 0.1. LC/MS: *m*/*z* calculated for C_11_H_13_N_3_OSi [M + H]^+^: 232.1, found: 231.8.

#### Compound **7**: 5-Ethynyl-3,7-dihydro-4*H*-pyrrolo­[2,3-*d*]­pyrimidin-4-one

Intermediate **6** (30 mg, 0.129 mmol, 1.0 equiv) was dissolved under an argon atmosphere in anhydrous acetonitrile (5.0 mL). Subsequently, TBAF (1 m in THF, 261 μL, 0.261 mmol, 2.0 equiv) was added dropwise at 0 °C. The reaction was stirred for 1.5 h at room temperature before it was terminated by the addition of 2.0 mL of saturated ammonium chloride solution. The solution was lyophilized, and the crude product was purified via reverse-phase flash chromatography. Yield: 14 mg, 68%. ^1^H NMR (300 MHz, DMSO-*d*_6_) δ [ppm] = 7.85 (s, 1H), 7.38 (s, 1H), 3.90 (s, 1H). ^13^C NMR (75 MHz, DMSO) δ [ppm] = 157.7, 147.8, 144.6, 126.1, 107.4, 97.7, 80.6, 77.9. LC/MS: *m*/*z* calculated for C_8_H_5_N_3_O [M + H]^+^: 160.0, found: 159.9.

#### Compound **8a**: *N*-(3-(4-(4-Oxo-4,7-dihydro-3*H*-pyrrolo­[2,3-*d*]­pyrimidin-5-yl)-1*H*-1,2,3-triazol-1-yl)­propyl)­acetamide

The compound was synthesized according to the general procedure for *in situ* click reactions and to general procedure for preparative click reactions with alkyne **7** and azide **15**. Yield (preparative): 2.0 mg, 51%. LC/MS: *m*/*z* calculated for C_13_H_15_N_7_O_2_ [M + H]^+^: 302.1, found: 302.0.

#### Compound **8b**: 3-((4-(4-Oxo-4,7-dihydro-3*H*-pyrrolo­[2,3-*d*]­pyrimidin-5-yl)-1*H*-1,2,3-triazol-1-yl)­methyl)­benzonitrile

The compound was synthesized according to the general procedure for preparative click reactions with alkyne **7** and commercially available 3-(azidomethyl)­benzonitrile. Yield: 2.5 mg, 25%. ^1^H NMR (400 MHz, DMSO-*d*_6_) δ [ppm] = 8.76 (s, 1H), 7.84 (d, *J* = 15.0 Hz, 3H), 7.67–7.53 (m, 3H), 5.75 (s, 2H). ^13^C NMR (101 MHz, DMSO) δ [ppm] = 143.9, 138.0, 132.8, 131.9, 131.5, 130.1, 122.9, 118.5, 118.1, 117.3, 111.7, 109.5, 104.1, 51.7. LC/MS: *m*/*z* calculated for C_16_H_11_N_7_O [M + H]^+^: 318.1, found: 317.9. Purity (HPLC): 96%.

#### Compound **8c**: 5-(1-((8,8-Difluorobicyclo[5.1.0]­octan-4-yl)­methyl)-1*H*-1,2,3-triazol-4-yl)-3,7-dihydro-4*H*-pyrrolo­[2,3-*d*]­pyrimidin-4-one

The compound was synthesized according to the general procedure for preparative click reactions with alkyne **7** and commercially available 4-(azidomethyl)-8,8-difluorobicyclo[5.1.0]­octane. Yield: 3 mg, 28%. ^1^H NMR (400 MHz, DMSO-*d*_6_) δ [ppm] = 8.64 (t, *J* = 4.1 Hz, 1H), 7.86 (s, 1H), 7.52 (d, *J* = 3.0 Hz, 1H), 4.39 (d, *J* = 8.0 Hz, 2H), 1.96–1.41 (m, 9H), 1.21 (q, *J* = 13.4, 12.1 Hz, 3H). ^13^C NMR (101 MHz, DMSO) δ [ppm] = 158.8, 148.7, 143.9, 123.1, 117.1, 109.7, 104.1, 55.4, 50.8, 44.0, 37.1, 32.0, 29.3, 25.1, 25.0, 24.9, 20.4, 16.7. LC/MS: *m*/*z* calculated for C_17_H_18_F_2_N_6_O [M + H]^+^: 361.2, found: 361.1. Purity (HPLC): 100%.

#### Compound **8d**: 5-(1-(Quinolin-6-ylmethyl)-1*H*-1,2,3-triazol-4-yl)-3,7-dihydro-4*H*-pyrrolo­[2,3-*d*]­pyrimidin-4-one

The compound was synthesized according to the general procedure for preparative click reactions with alkyne **7** and commercially available 6-(azidomethyl)­quinoline. Yield: 6 mg, 56%. ^1^H NMR (400 MHz, DMSO-*d*_6_) δ [ppm] = 8.93–8.81 (m, 1H), 8.58 (t, *J* = 7.2 Hz, 1H), 8.26–7.49 (m, 6H), 5.92 (s, 2H). ^13^C NMR (101 MHz, DMSO) δ [ppm] = 148.5, 143.9, 137.7, 135.2, 130.0, 129.9, 127.1, 126.9, 117.2, 117.0, 109.6, 52.3, 52.2. LC/MS: *m*/*z* calculated for C_18_H_13_N_7_O [M + H]^+^: 344.2, found:344.0. Purity (HPLC): 99%.

#### Compound **8e**: 5-(1-(((1*R*,2*R*)-2-Aminocyclopentyl)­methyl)-1*H*-1,2,3-triazol-4-yl)-3,7-dihydro-4*H*-pyrrolo­[2,3-*d*]­pyrimidin-4-one

The compound was synthesized according to the general procedure for preparative click reactions with alkyne **7** and commercially available (1*R*,2*R*)-2-(azidomethyl)­cyclopentan-1-amine. Yield: 6 mg, 64%. ^1^H NMR (400 MHz, DMSO-*d*_6_) δ [ppm] = 8.73 (s, 1H), 7.98 (s, 2H), 7.87 (d, *J* = 3.5 Hz, 1H), 7.56–7.52 (m, 1H), 4.57 (dd, *J* = 13.7, 5.7 Hz, 1H), 4.41 (dd, *J* = 13.7, 9.8 Hz, 1H), 3.70–3.66 (m, 1H), 2.61 (dq, *J* = 13.0, 6.7 Hz, 1H), 2.02 (dh, *J* = 13.1, 6.1 Hz, 1H), 1.86–1.67 (m, 2H), 1.64–1.46 (m, 3H). ^13^C NMR (101 MHz, DMSO) δ [ppm] = 158.8, 148.8, 144.0, 122.8, 117.2, 109.6, 104.1, 52.9, 48.6, 42.7, 30.0, 26.6, 20.9. LC/MS: *m*/*z* calculated for C_14_H_17_N_7_O [M + H]^+^: 300.2, found: 300.1. Purity (HPLC): 96%.

#### Compound **8f**: Di-Cbz-1-(3-(4-(4-oxo-4,7-dihydro-3*H*-pyrrolo­[2,3-*d*]­pyrimidin-5-yl)-1*H*-1,2,3-triazol-1-yl)­propyl)­guanidine

The compound was synthesized according to the general procedure for preparative click reactions with alkyne **7** and azide **13**. Yield: 4 mg, 29%. ^1^H NMR (400 MHz, DMSO-*d*_6_) δ [ppm] = 8.68 (s, 1H), 8.62 (t, *J* = 5.7 Hz, 1H), 7.85 (s, 1H), 7.54–7.52 (m, 1H), 7.38 (dd, *J* = 8.4, 3.5 Hz, 6H), 7.31 (d, *J* = 3.1 Hz, 4H), 5.19 (s, 2H), 5.00 (s, 2H), 4.59 (t, *J* = 5.9 Hz, 2H), 3.81 (q, *J* = 5.8 Hz, 2H). ^13^C NMR (101 MHz, DMSO) δ [ppm] = 162.9, 158.8, 155.4, 152.4, 148.7, 143.9, 141.3, 136.8, 135.1, 128.6, 128.5, 128.4, 128.3, 128.0, 127.8, 123.0, 117.1, 109.8, 104.2, 67.7, 66.5, 48.1, 40.8. LC/MS: *m*/*z* calculated for C_27_H_25_N_9_O_5_ [M + H]^+^: 556.2, found: 556.0. Purity (HPLC): 98%.

#### Compound **8g**: Di-Boc-1-(3-(4-(4-oxo-4,7-dihydro-3*H*-pyrrolo­[2,3-*d*]­pyrimidin-5-yl)-1*H*-1,2,3-triazol-1-yl)­propyl)­guanidine

The compound was synthesized according to the general procedure for preparative click reactions with alkyne **7** and azide **14**. Yield: 9 mg, 74%. ^1^H NMR (400 MHz, DMSO-*d*_6_) δ [ppm] = 8.68 (s, 1H), 8.49 (t, *J* = 5.7 Hz, 1H), 7.85 (d, *J* = 3.3 Hz, 1H), 7.52 (d, *J* = 2.4 Hz, 1H), 4.58 (t, *J* = 5.9 Hz, 2H), 3.76 (q, *J* = 5.9 Hz, 2H), 1.45 (s, 9H), 1.36 (s, 9H). ^13^C NMR (101 MHz, DMSO) δ [ppm] = 163.0, 158.8, 155.5, 151.7, 148.7, 143.9, 141.2, 123.0, 117.0, 109.8, 104.2, 82.9, 78.3, 48.2, 27.9, 27.6. LC/MS: *m*/*z* calculated for C_21_H_29_N_9_O_5_ [M + H]^+^: 488.2, found: 488.0. Purity (HPLC): 96%.

#### Compound **8h**: 1-(3-(4-(4-Oxo-4,7-dihydro-3*H*-pyrrolo­[2,3-*d*]­pyrimidin-5-yl)-1*H*-1,2,3-triazol-1-yl)­propyl)­guanidine

The compound was synthesized according to the general procedure for preparative click reactions with azide **14**. Subsequently, DCM (0.5 mL) and TFA (0.5 mL) were added to the residues, and the mixture was stirred for 30 min at room temperature. Afterward, the solvents were removed *in vacuo*, and the crude product was purified via reverse-phase flash chromatography. Yield: 3 mg, 32%. ^1^H NMR (400 MHz, DMSO-*d*_6_) δ [ppm] = 12.14 (s, 1H), 11.93 (d, *J* = 3.8 Hz, 1H), 8.73 (s, 1H), 7.87 (d, *J* = 3.2 Hz, 1H), 7.54 (m, 1H), 4.54 (t, *J* = 6.0 Hz, 2H), 3.69–3.63 (m, 2H), 2.54 (s, 1H), 1.39–1.16 (m, 2H). ^13^C NMR (101 MHz, DMSO) δ [ppm] = 158.8, 156.9, 156.8, 148.8, 143.9, 141.3, 123.0, 117.2, 109.6, 104.1, 48.3, 40.8. LC/MS: *m*/*z* calculated for C_11_H_13_N_9_O [M + H]^+^: 288.1, found: 288.0. Purity (HPLC): 98%.

#### Compound **10**: 4-Oxo-*N*-(prop-2-yn-1-yl)-3,4-dihydrothieno­[2,3-*d*]­pyrimidine-5-carboxamide

4-Oxo-3,4-dihydrothieno­[2,3-*d*]­pyrimidine-5-carboxylic acid (12 mg, 0.061 mmol, 1.0 equiv), DIPEA (43 μL, 0.244 mmol, 4.0 equiv), and HATU (23 mg, 0.061 mmol, 1.0 equiv) were dissolved in 3 mL anhydrous DCM. The mixture was stirred for 15 min at room temperature before propargylamine (5 μL, 0.073 mmol, 1.2 equiv) was added in one portion. The reaction was stirred for 16 h at room temperature. The reaction mixture was washed with a saturated sodium bicarbonate solution and 1 m HCl. The crude reaction mixture was purified via reverse-phase flash chromatography. Yield: 11 mg, 77%. ^1^H NMR (300 MHz, DMSO-*d*_6_) δ [ppm] = 8.39 (s, 1H), 8.29 (s, 1H), 4.13 (dd, *J* = 5.1, 2.5 Hz, 2H), 3.17 (t, *J* = 2.5 Hz, 1H). ^13^C NMR (75 MHz, DMSO) δ [ppm] = 167.1, 159.9, 159.6, 146.0, 131.8, 131.6, 119.2, 80.6, 73.5, 28.5. LC/MS: *m*/*z* calculated for C_10_H_7_N_3_O_2_S [M + H]^+^: 234.0, found: 233.9.

#### Compound **12**: 2-Azidoethan-1-amine

2-Chloroethylamine hydrochloride (100 mg, 0.862 mmol, 1.0 equiv) was dissolved in water (5.0 mL). Sodium azide (168 mg, 2.586 mmol, 3.0 equiv) was added, and the reaction was allowed to stir for 12 h at 90 °C. Subsequently, a 15% potassium hydroxide solution (8.0 mL) was added, and the product was extracted with diethyl ether. The organic solvent was removed *in vacuo*. Yield: 85 mg, quant. ^1^H NMR (300 MHz, C*D*Cl_3_) δ [ppm] = 3.36 (t, *J* = 5.7 Hz, 2H), 2.87 (t, *J* = 5.7 Hz, 2H), 1.51 (s, 2H). ^13^C NMR (75 MHz, *C*DCl_3_) δ [ppm] = 77.5, 77.1, 76.6, 54.6, 53.5, 41.4.

#### Compound **13**: Benzyl *N*-[(1*Z*)-[(2-azidoethyl)­amino]­({[(benzyloxy)­carbonyl]­amino}) methylidene]­carbamate

*N,N′*-Di-Cbz-1*H*-pyrazol-1-carbamidine (380 mg, 1.005 mmol, 1.0 equiv) was dissolved in THF (2.0 mL), and **12** (185 mg, 2.150 mmol, 2.2 equiv) in THF (2.0 mL) was added dropwise. One drop of triethylamine was added, and the reaction was allowed to stir for 16 h at room temperature. The solvent was removed *in vacuo*, and the crude product was purified via reverse-phase flash chromatography. Yield: 357 mg, 90%. ^1^H NMR (300 MHz, C*D*Cl_3_) δ [ppm] = 8.54 (s, 1H), 7.34–7.14 (m, 10H), 5.06 (d, *J* = 11.0 Hz, 5H), 3.43 (dt, *J* = 33.8, 5.2 Hz, 4H). ^13^C NMR (75 MHz, *C*DCl_3_) δ [ppm] = 163.0, 156.0, 153.6, 136.4, 134.4, 128.8, 128.6, 128.42, 128.35, 128.1, 128.0, 68.3, 67.3, 50.1, 40.3. LC/MS: *m*/*z* calculated for C_19_H_20_N_6_O_4_ [M + H]^+^: 397.2, found: 397.1.

#### Compound **14**: [*N,N*′-Bis­(*tert*-butoxycarbonyl)-*N*″-2-azidoethyl]­guanidine

*N,N′*-Di-Boc-1*H*-pyrazol-1-carbamidine (310 mg, 1.005 mmol, 1.0 equiv) was dissolved in THF (2.0 mL), and **12** (85 mg, 1.000 mmol, 1.0 equiv) in THF (1.0 mL) was added dropwise. One drop of triethylamine was added, and the reaction was allowed to stir for 72 h at room temperature. The solvent was removed *in vacuo*, and the crude product was purified via reverse-phase flash chromatography. Yield: 153 mg, 47%. ^1^H NMR (300 MHz, DMSO-*d*_6_) δ [ppm] = 3.49 (s, 4H), 3.37 (s, 2H), 1.48 (d, *J* = 3.9 Hz, 9H), 1.39 (s, 9H). ^13^C NMR (75 MHz, DMSO) δ [ppm] = 83.1, 78.3, 49.4, 28.0, 27.6. LC/MS: *m*/*z* calculated for C_13_H_24_N_6_O_4_ [M + H]^+^: 329.2, found: 329.0.

#### Compound **15**: *N*-(3-Azidopropyl)­acetamide

3-Azidopropan-1-amine (20 mg, 0.200 mmol, 1.0 equiv), acetic anhydride (30 mg, 0.249 mmol, 1.5 equiv), and acetic acid (40 μL) were mixed at room temperature. The reaction was left to stand for 3 min, then 1.0 mL acetonitrile was added, and the mixture was lyophilized. Yield: 22 mg, 77%. ^1^H NMR (300 MHz, C*D*Cl_3_) δ [ppm] = 3.32 (dt, *J* = 16.9, 6.6 Hz, 4H), 1.96 (s, 3H), 1.77 (p, *J* = 6.7 Hz, 2H). ^13^C NMR (75 MHz, *C*DCl_3_) δ [ppm] = 170.6, 49.4, 37.3, 28.8, 23.3. LC/MS: *m*/*z* calculated for C_5_H_10_N_4_O [M + H]^+^: 143.1, found: 143.0.

## Supplementary Material







## Notes

Supplementary results, NMR, and LC/MS spectra (PDF)
Supplementary Data set including azide library decoding and SMILES strings (XLSX)
Molecular formula strings and associated biochemical and biological data (CSV)

## Abbreviations Used

ACN: acetonitrile
ANOVA: analysis of variance
CuAAC: copper-catalyzed alkyne–azide click reaction
D2B: direct-to-biology
DCM: dichloromethane
DIPEA: *N*,*N*-diisopropylethylamine
DMF: dimethylformamide
DMSO: dimethyl sulfoxide
FP: fluorescence polarization
HATU: *O*-(7-azabenzotriazol-1-yl)-*N*,*N*,*N*′,*N*′-tetramethyluronium hexafluorphosphate
HEPES: 2-[4-(2-hydroxyethyl)­piperazin-1-yl]­ethanesulfonic acid
IPTG: isopropyl-β-d-1-thiogalactopyranoside
ITC: isothermal titration calorimetry
LB: lysogeny broth
m^1^G: *N* ^1^-methylguanosine
MBC: minimum bactericial concentration
MTase: methyltransferase
NC: negative control
n. d.: not determined
PC: positive control PMSF, phenylmethylsulfonyl fluoride
quant.: quantitative
rt: room temperature
SAH: *S*-adenosylhomocysteine
SAM: *S*-adenosylmethionine
SEC: size exclusion chromatography
SPOUT: trefoil knot protein fold
TBAF: tetra-*n*-butylammonium fluoride
TBTA: *tris*((1-benzyl-4-triazolyl)­methyl)­amine
TCEP: *tris*(2-carboxyethyl)­phosphine
TFA: trifluoroacetic acid
THF: tetrahydrofuran
TrmD: tRNA (guanine-*N* ^1^-)-methyltransferase
