# Supplementary material for: Nanoscale Direct-to-Biology Optimization and Structural Insights into Selective S. aureus TrmD Inhibitors
Source: J Med Chem. 2025 Dec 10;68(24):26246–62. doi: 10.1021/acs.jmedchem.5c02323 (PMC12751012; doi:10.1021/acs.jmedchem.5c02323)

Supporting Information

Nanoscale Direct-to-Biology Optimization and Structural Insights into Selective *S. aureus* TrmD Inhibitors

Ariane F. Hübner[‡,a], Annabelle C. Weldert[‡,a], Tessa Marciniak[b], Florian Hof[c], Vivien S. Beck[b], Samuel Carien[b], Sophie N. Mulartschyk[a], Eva Wolf[c], Wilma Ziebuhr[b], Fabian Barthels[*,a]

[a]Institute of Pharmaceutical and Biomedical Sciences, Johannes Gutenberg-University, Staudingerweg 5, 55128 Mainz, Germany.

[b]Institute of Molecular Infection Biology, University of Würzburg, Josef-Schneider-Strasse 2, 97080 Würzburg, Germany.

[c]Institute of Molecular Physiology, Johannes Gutenberg-University, Hanns-Dieter-Hüsch-Weg 17, 55128 Mainz, Germany.

Corresponding Author's email address: barthels@uni-mainz.de

Table of contents

Crystallography of parent compounds....S3

Isothermal titration calorimetry (ITC)....S3

Supplementary results of the nanoSAR screening....S4

Fluorescence polarization (FP) assays and selectivity....S4

S. aureus TrmD inhibition (aptamer-based enzyme assay)....S6

TrmD inhibition (^{3}H incorporation enzyme assay)....S6

Cell viability assay (Cell Titer Glo)....S7

Supplementary results of anti-bacterial experiments....S7

Carbamate cleavage assays (LC/MS lysate assay)....S9

Supplementary crystallographic figures....S11

Physicochemical properties of the azide-library....S17

Docking poses compounds 2 and 8b-e,h....S18

NMR and LC/MS Spectra....S19

Crystallography of parent compounds

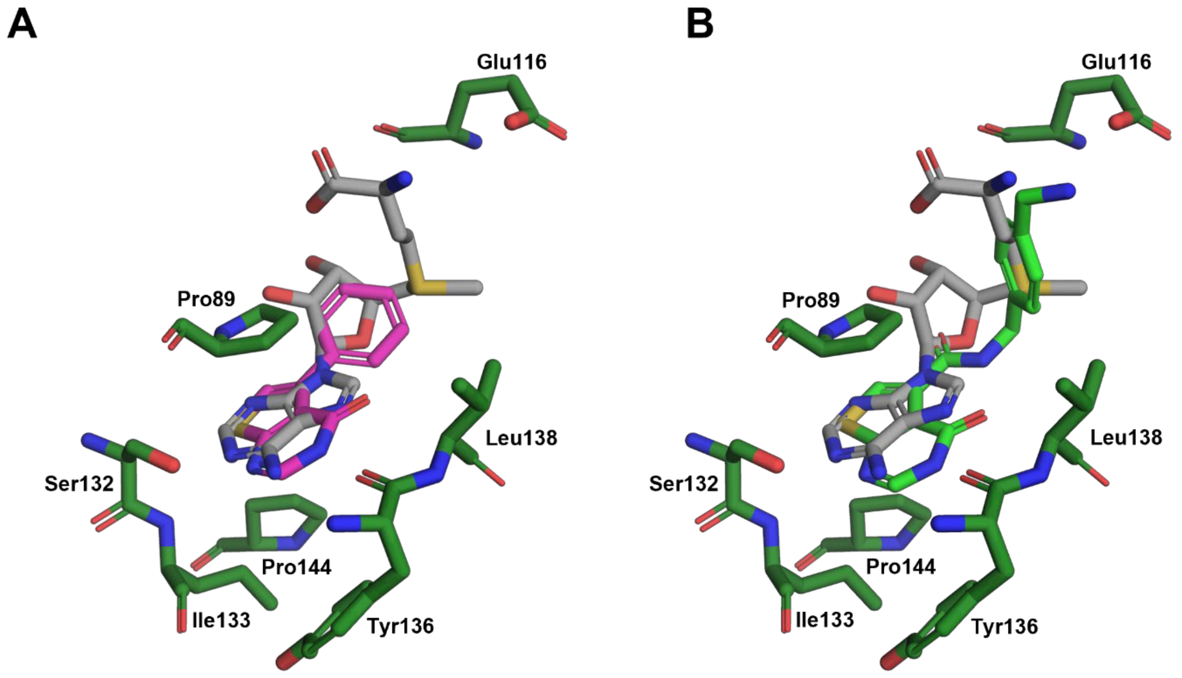

Figure S1: Parent structures for the development of *S. aureus* TrmD inhibitors. Crystallographic overlay with SAM (PDB:1UAK) of literature-known compounds **1** (**A**, PDB: 4MCD) and **2** (**B**, PDB 4MCC) in the *H. influenzae* SAM-binding pocket. Ligands and amino acids are shown in stick representation. The carbon atoms of SAM are shown in grey, those of **1** are shown in pink, and those of compound **2** are shown in lime. Interacting residues are displayed in dark green.

Isothermal titration calorimetry (ITC)

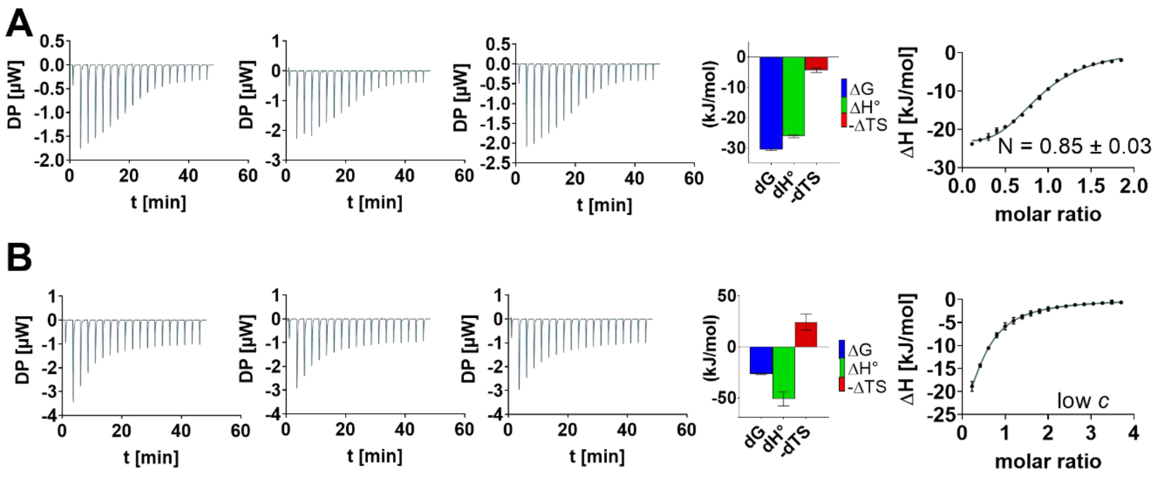

Figure S2. ITC data for the titration of compounds **1** and **2** against *Haemophilus influenzae* TrmD. (**A**) Compound **1** was titrated into *Haemophilus influenzae* TrmD. (**B**) Compound **2** was titrated into *Haemophilus influenzae* TrmD. Note: a low-c titration was performed for compound **2**.

Supplementary results of the nanoSAR screening

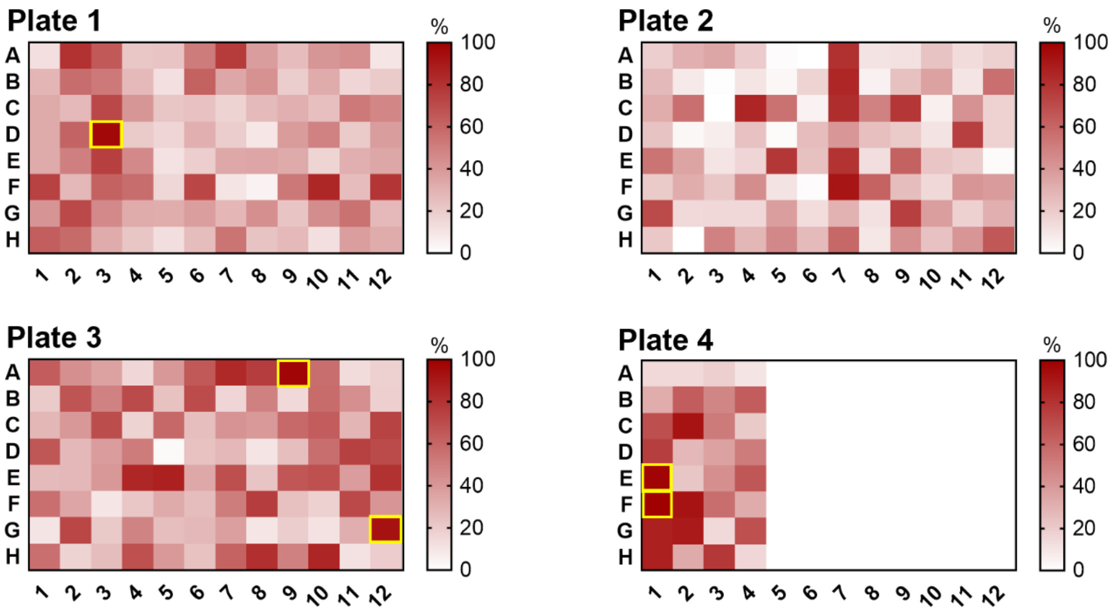

Figure S3. Results of the nanoSAR FP screening are presented in a heat-map format (displaced tracer [%]). The hit compounds **8b–e,h** are highlighted in yellow.

Fluorescence polarization (FP) assays and selectivity

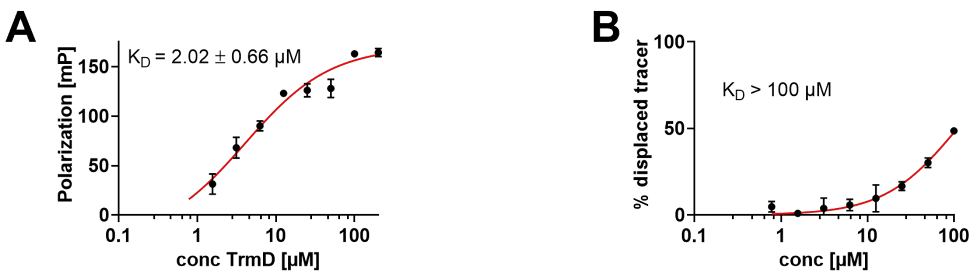

Figure S4. (**A**) Fluorescence polarization assay with tracer **3** titrated against *H. influenzae* TrmD. (**B**) K$_D$-value determination of alkyne **7** titrated against *S. aureus* TrmD.

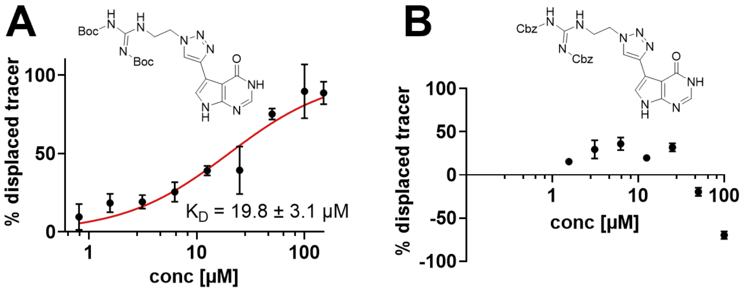

Figure S5. Supplementary FP-assay data on *S. aureus* TrmD. (**A**) **8f** (**B**) **8g.**

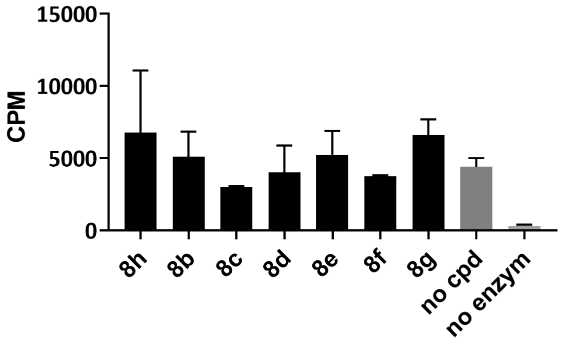

Figure S6. ^{3}H-based enzyme assay for Trm5 selectivity analysis of compound **8b**–**h**. Compounds were assayed at a final concentration of 30 µM, exhibiting no significant inhibition.

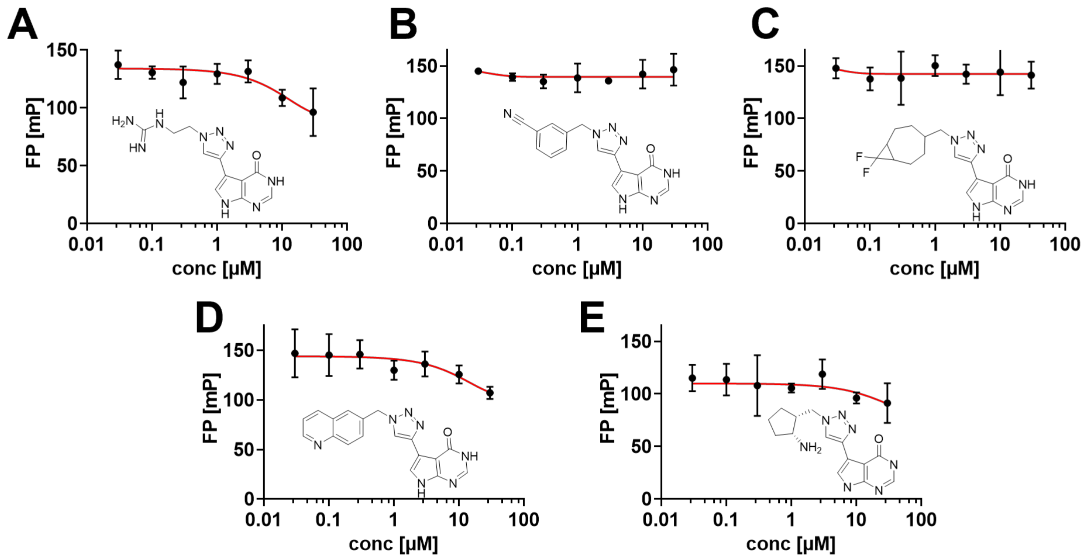

Figure S7. FP selectivity assays on *H. influenzae* TrmD for (**A**) **8h** (**B**) **8b** (**C**) **8c** (**D**) **8d** (**E**) **8e**.

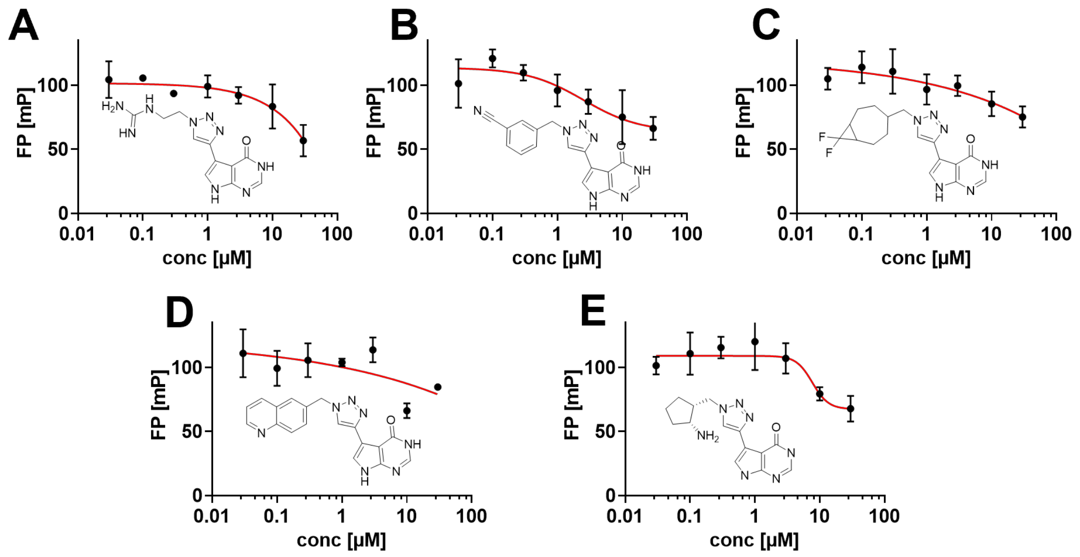

Figure S8. FP selectivity assays on *E. coli* TrmD for (**A**) **8h** (**B**) **8b** (**C**) **8c** (**D**) **8d** (**E**) **8e**.

***S. aureus* TrmD inhibition (aptamer-based enzyme assay)**

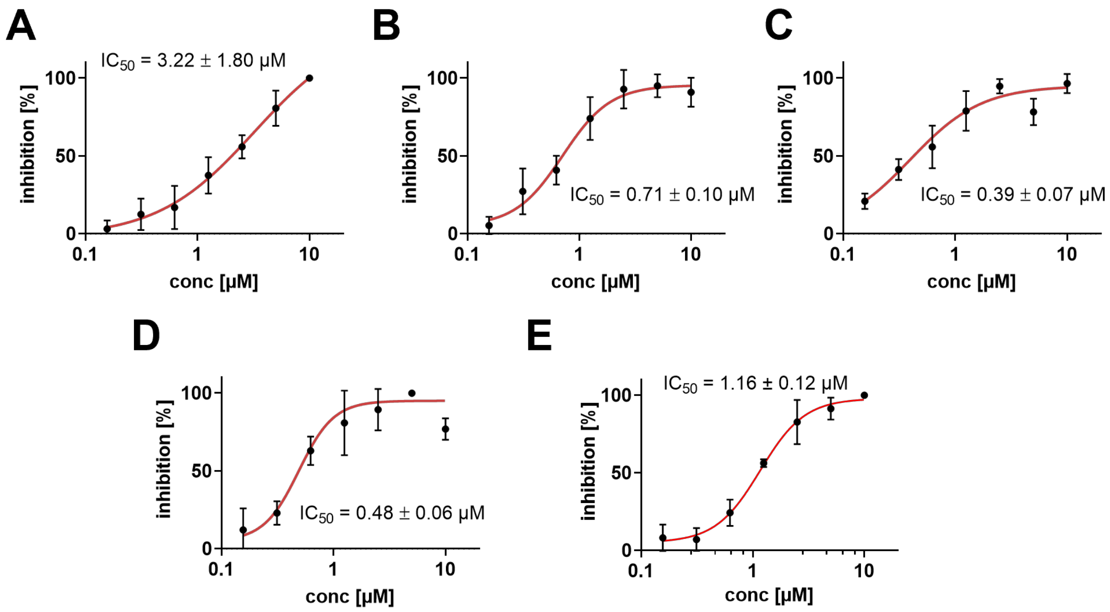

Figure S9. *S. aureus* TrmD inhibition measured by aptamer-based enzyme assay for the determination of IC_{50} values. (**A**) **8b** (**B**) **8c** (**C**) **8d** (**D**) **8e** (**E**) **8h**.

TrmD inhibition (^{3}H incorporation enzyme assay)

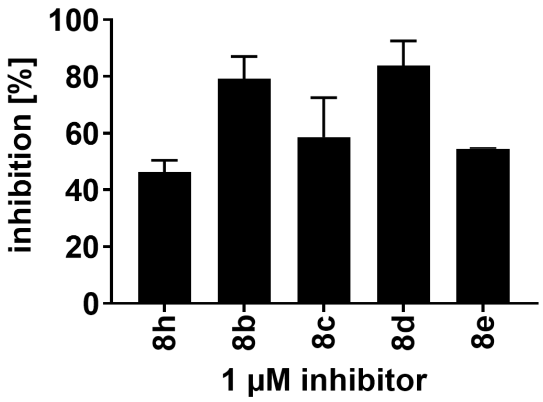

Figure S10. *S. aureus* TrmD inhibition measured by ^{3}H incorporation assay. Compounds were assayed at a final concentration of 1 µM, exhibiting inhibition values between 46% and 84%, confirming the inhibitory potency determined by aptamer-based assays (Figure S9).

Cell viability assay (Cell Titer Glo)

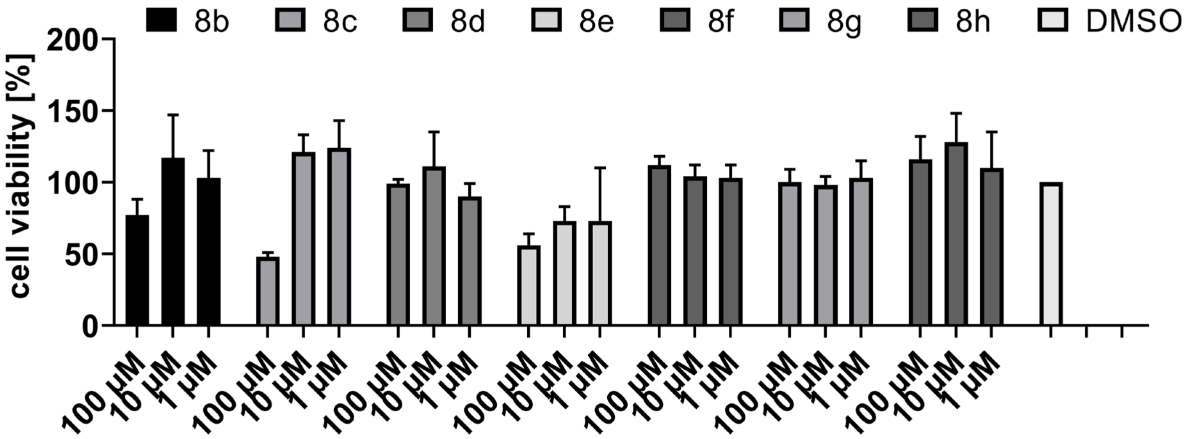

Figure S11. Screening results of the cell viability assay (HEK293). Pure DMSO was used as a control and set to 100% cell viability.

Supplementary results of anti-bacterial experiments

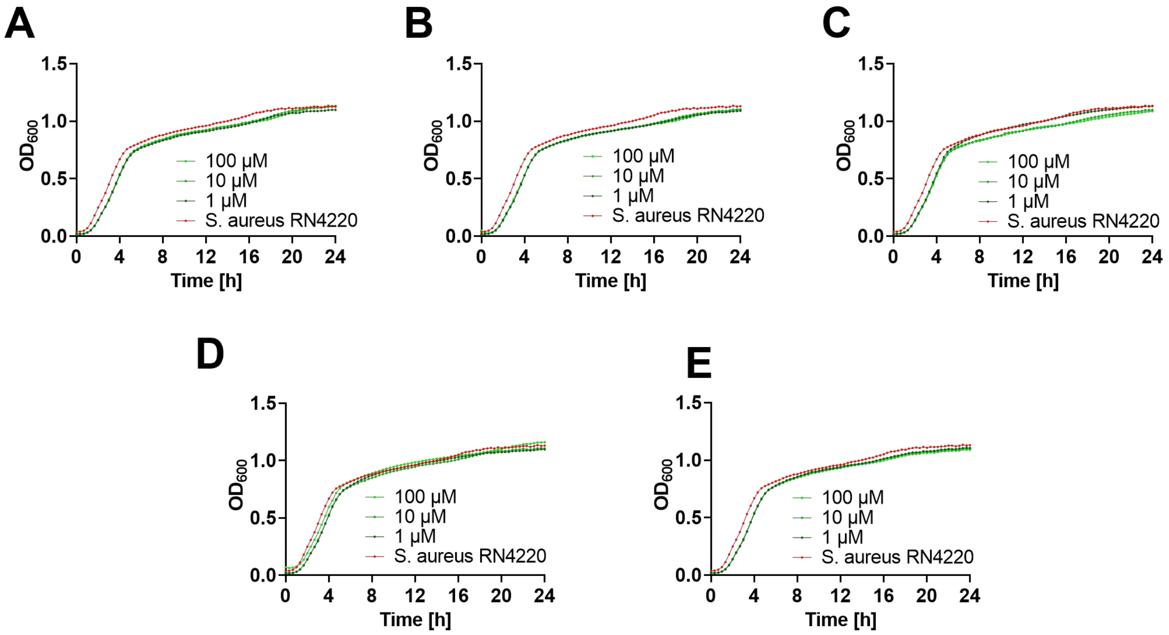

Figure S12. Growth curves of *S. aureus* RN4220 demonstrating the effect of compounds on bacterial growth. Compared to the negative control, no pronounced effect on growth was observed. (**A**) **8h** (**B**) **8b** (**C**) **8c** (**D**) **8d** (**E**) **8e**.

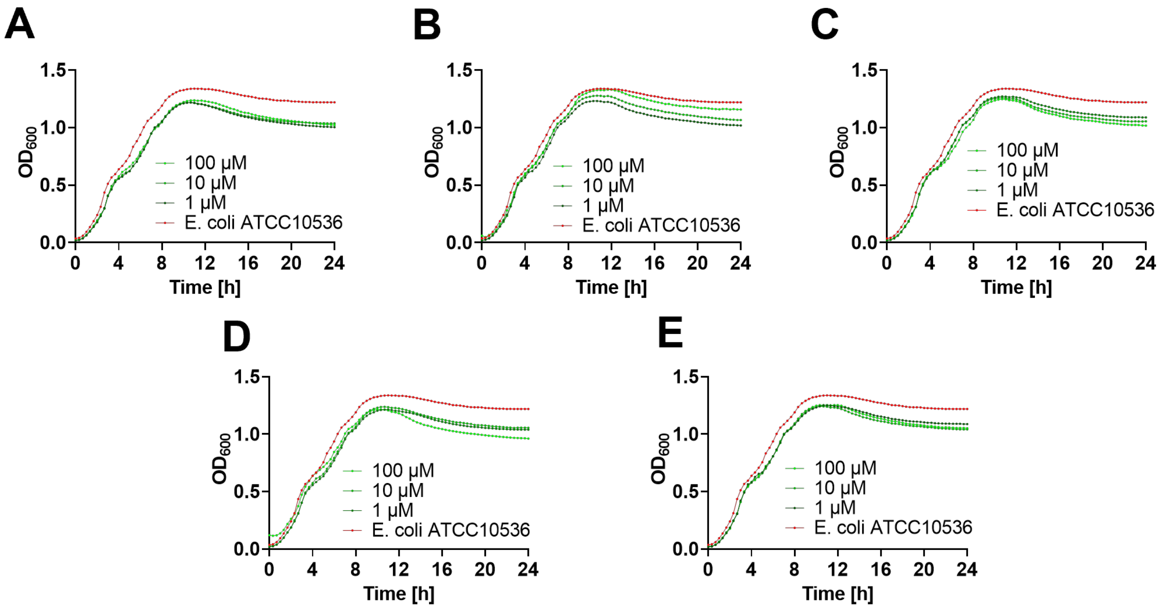

Figure S13. Growth curves of *E. coli* demonstrating the effect of compounds on bacterial growth. Compared to the negative control, no pronounced effect on growth was observed. (**A**) **8h** (**B**) **8b** (**C**) **8c** (**D**) **8d** (**E**) **8e**.

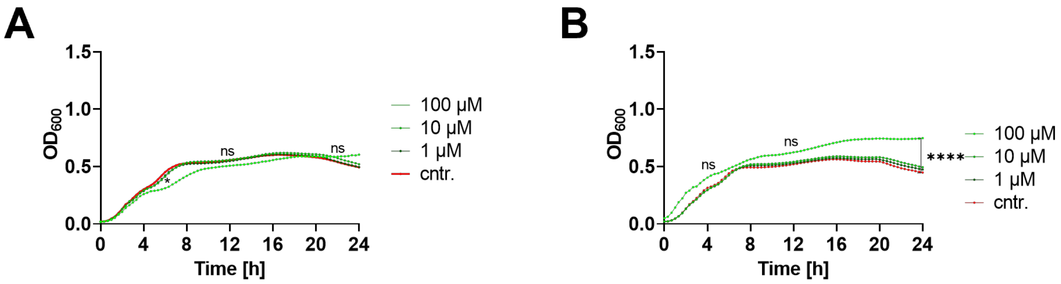

Figure S14. Growth curves of *E. coli* demonstrating the effect of **8g** (**A**) and **8f** (**B**) on bacterial growth. Compared to the negative control, no significant growth inhibition was observed. Two-way ANOVA with Dunnett's multiple comparisons test was used for statistical analysis, using a confidence interval of 95%.

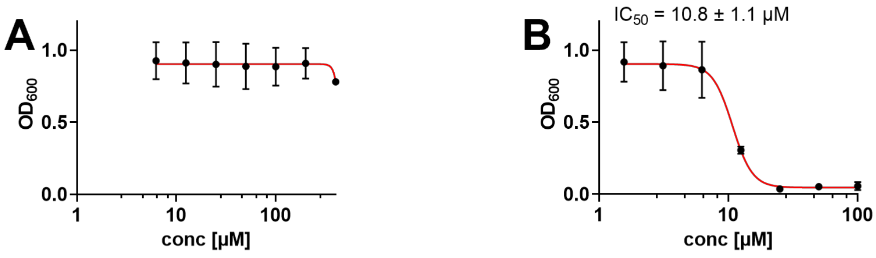

Figure S15. IC_{50} curves of **8g** (**A**) and **8f** (**B**) affecting *S. aureus* growth at 1440 min.

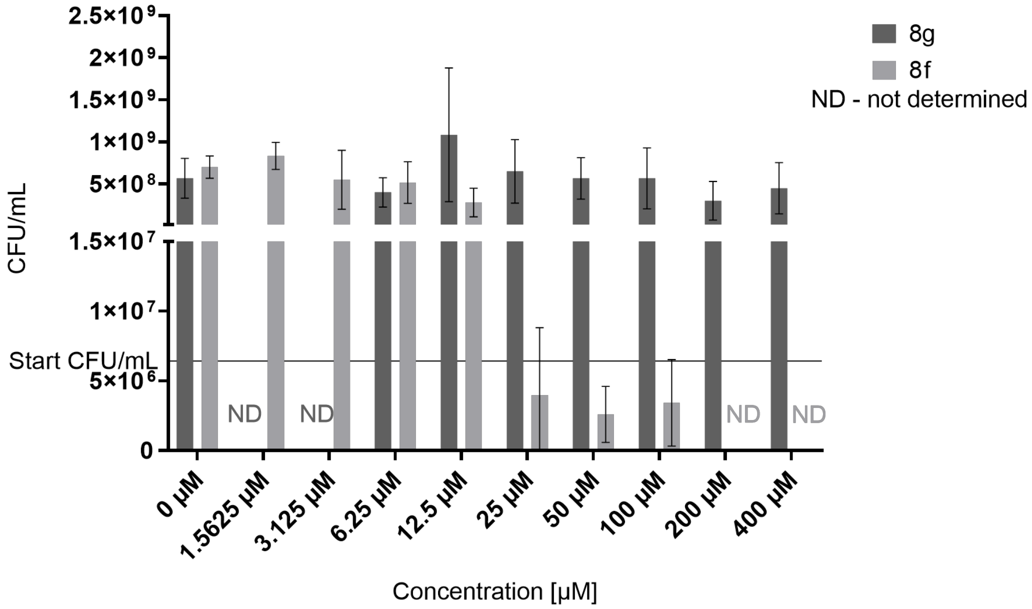

Figure S16. MBC determination of compounds **8f** and **8g**. Only **8f** affects growth starting at 12.5 µM.

Carbamate cleavage assays (LC/MS lysate assay)

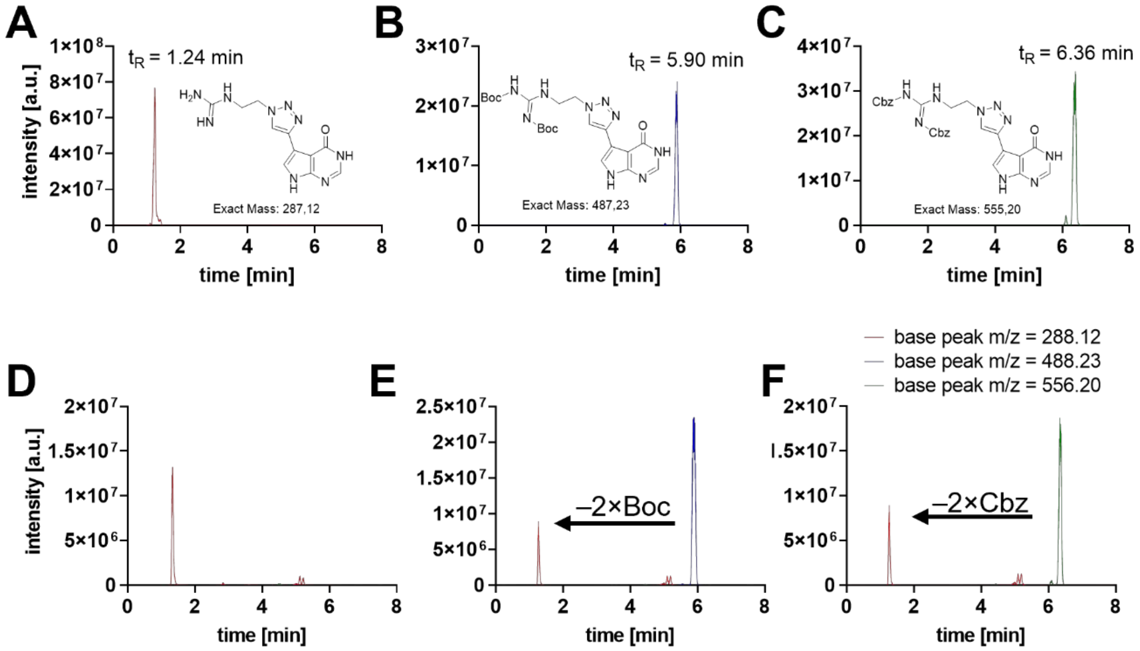

Figure S17. Supplementary results of the in vitro prodrug cleavage assay. (**A**) Basepeak chromatogram of pure reference compound **8h** m/z = 288±0.5. (**B**) Basepeak chromatogram of pure reference compound **8g** m/z = 488±0.5. (**C**) Basepeak chromatogram of pure reference compound **8f** m/z = 556±0.5. (**D**) Basepeak chromatogram of *S. aureus* lysate treated with **8h** m/z = 288±0.5. (**E**) Basepeak chromatogram of *S. aureus* lysate treated with **8g** m/z = 288±0.5; m/z = 488±0.5. (**F**) Basepeak chromatogram of *S. aureus* lysate treated with **8f** m/z = 288±0.5; m/z = 556±0.5.

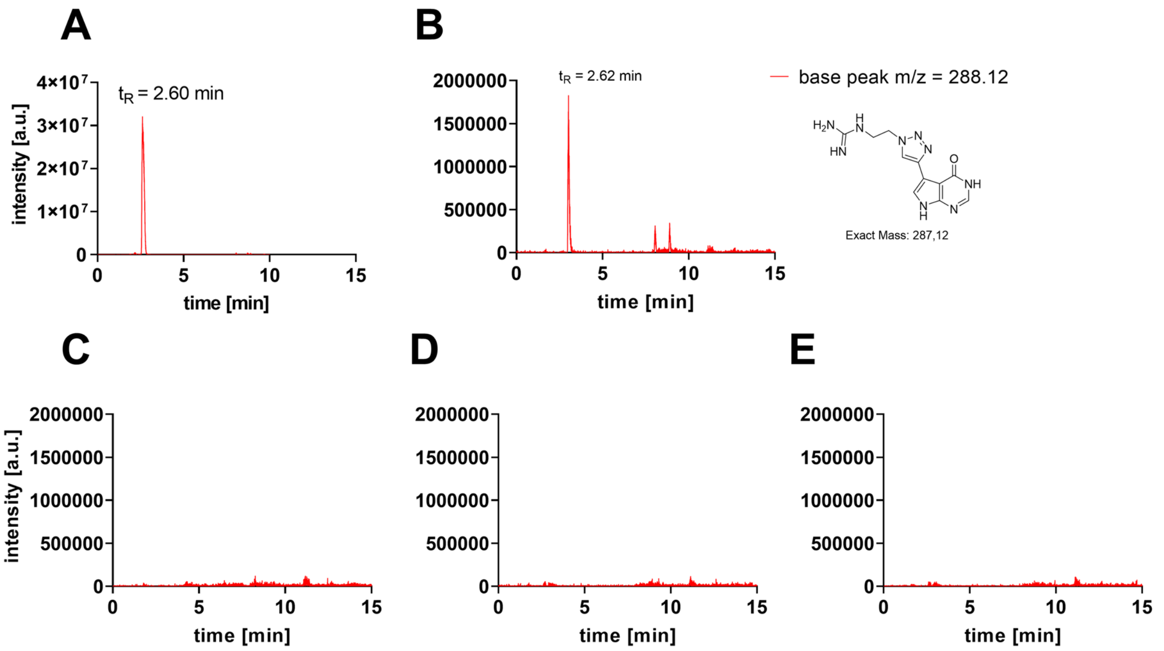

Figure S18. Supplementary results of the in vivo prodrug uptake and cleavage assay. (**A**) Basepeak chromatogram of pure reference compound **8h** m/z = 288±0.5. (**B**) Basepeak chromatogram of the sample derived from growing *S. aureus* culture incubated with compound **8f** and lysed after treatment. (**C**) Basepeak chromatogram of the sample derived from growing *S. aureus* culture incubated with compound **8g** and lysed after treatment. (**D**) Basepeak chromatogram of the sample derived from growing *S. aureus* culture incubated with compound **8h** and lysed after treatment. (**E**) Basepeak chromatogram of the sample derived from growing *S. aureus* culture mock-treated with DMSO and lysed after treatment.

Supplementary crystallographic figures

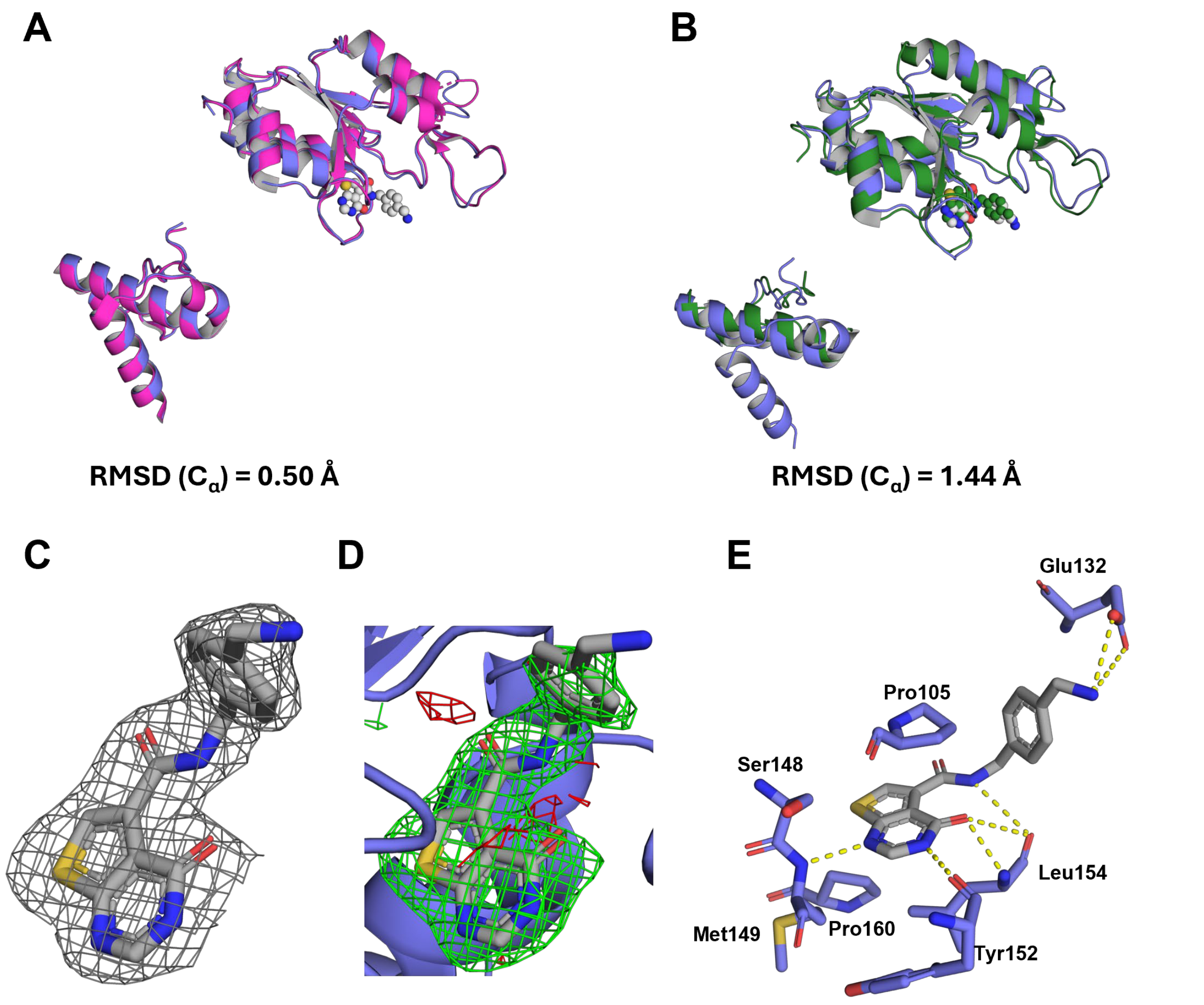

Figure S19. Supplementary crystallographic images for *S. aureus* TrmD co-crystallized with compound **2** (PDB: 9SDV). (**A**) Global superposition of the X-ray structures of *S. aureus* TrmD with compound **2** (violet-blue) and the apo structure (pink, 3KY7). (**B**) Global superposition of the X-ray structures of *S. aureus* TrmD with compound **2** (violet-blue) and *H. influenzae* TrmD with compound **2** (light blue, 4MCC). (**C**) Compound **2** bound *S. aureus* TrmD with $2mF_{obs}$-DF_{calc} electron density map at a contour level of +1σ. (**D**) Compound **2** bound *S. aureus* TrmD with mF_{obs}-DF_{calc} electron density map at a contour level of +3σ. (**E**) Compound **2** in the *S. aureus* SAH binding pocket. Protein structures are depicted as cartoons, interacting residues are shown as sticks, the ligand as spheres or in stick form, and polar interactions as yellow dashed lines.

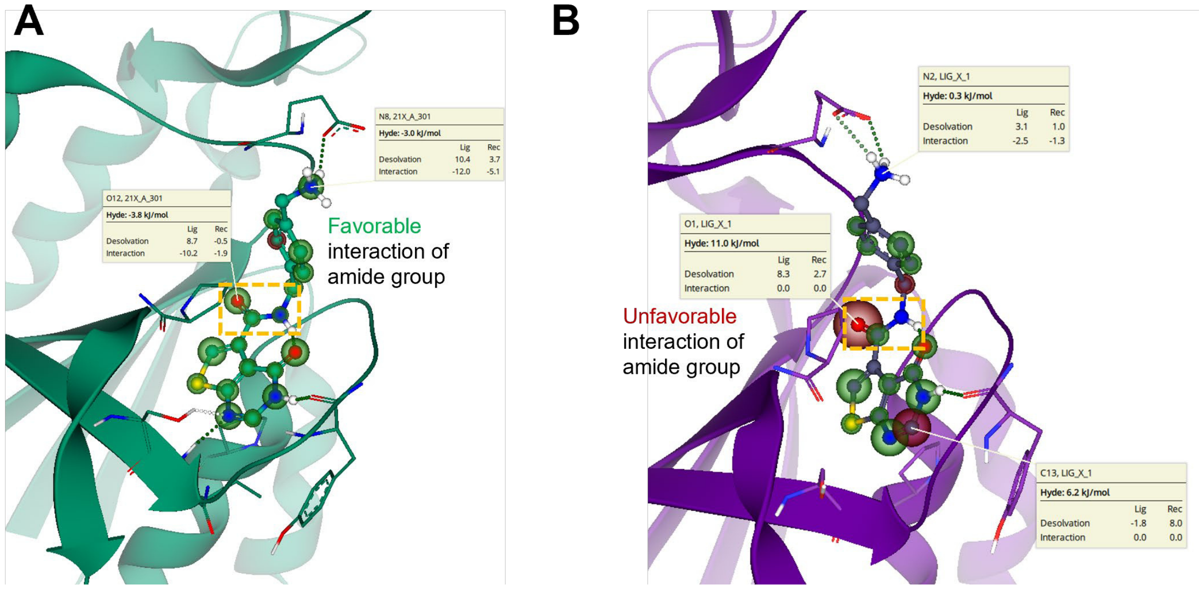

Figure S20. SeeSAR analysis by binding pose inspection of compound **2** bound to *H. influenzae* TrmD (**A**, green, PDB: 4MCC) and *S. aureus* TrmD (**B**, purple, PDB: 9SDV). Proteins are depicted as cartoons with representative binding site amino acids shown as lines. The compound is shown as stick. Hyde coloring enables the identification of favorable resp. unfavorable atom placements (colored spheres; green: favorable, red: unfavored).

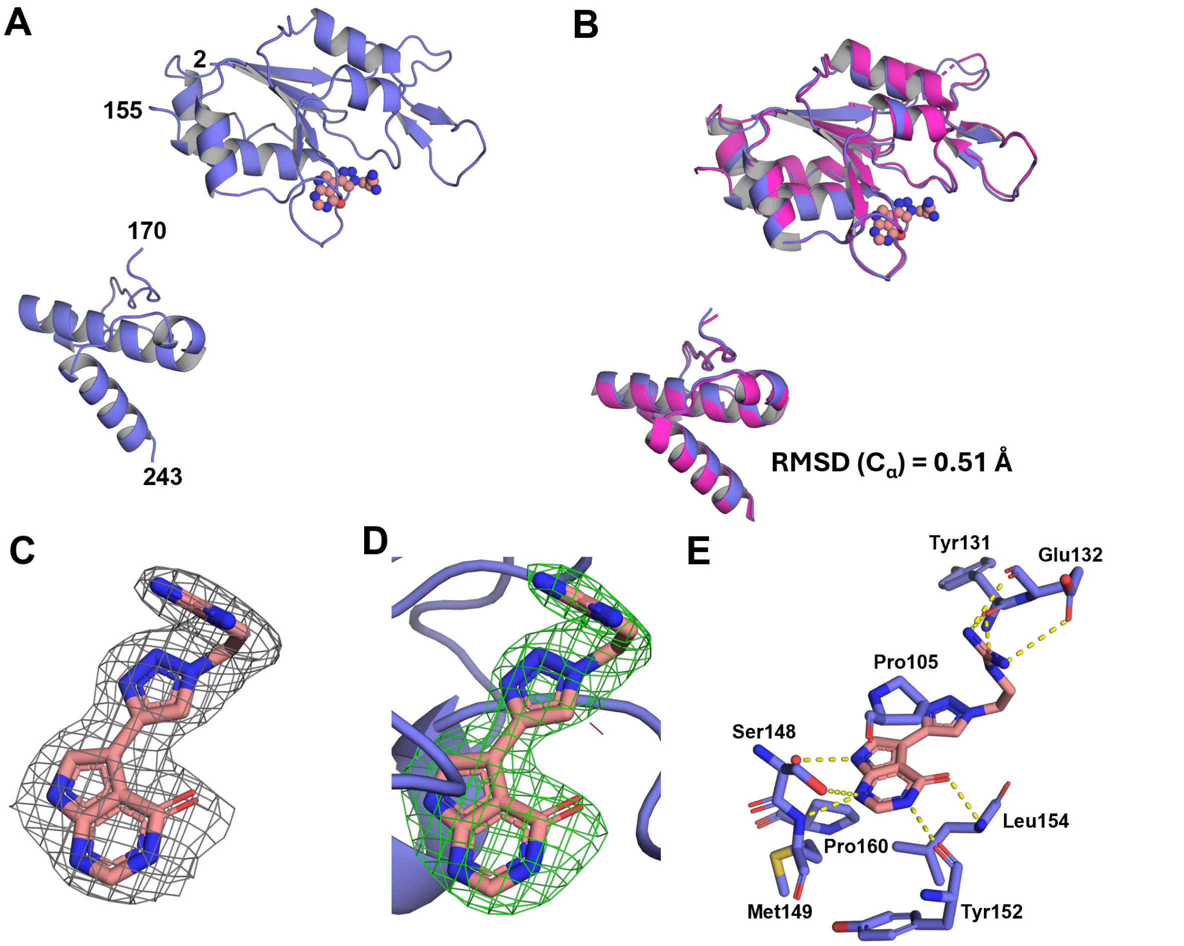

Figure S21. Supplementary crystallographic images for *S. aureus* TrmD co-crystallized with compound **8h** (PDB: 9SDW). (**A**) Structure of *S. aureus* TrmD bound to compound **8h.** (**B**) Global superposition of the X-ray structures of *S. aureus* TrmD with compound **8h** (violet-blue) and the apo structure (pink, 3KYZ). (**C**) Compound **8h** (salmon) bound *S. aureus* TrmD with $2mF_{obs}$-DF_{calc} electron density map at a contour level of +1σ. (**D**) Compound **8h** bound *S. aureus* TrmD with mF_{obs}-DF_{calc} electron density map at a contour level of +3σ. (**E**) Compound **8h** in the *S. aureus* SAH binding pocket. Protein structures are depicted as cartoons, interacting residues are shown as sticks, the ligand as spheres or in stick form, water molecules are shown as spheres, and polar interactions are shown as yellow dashed lines.

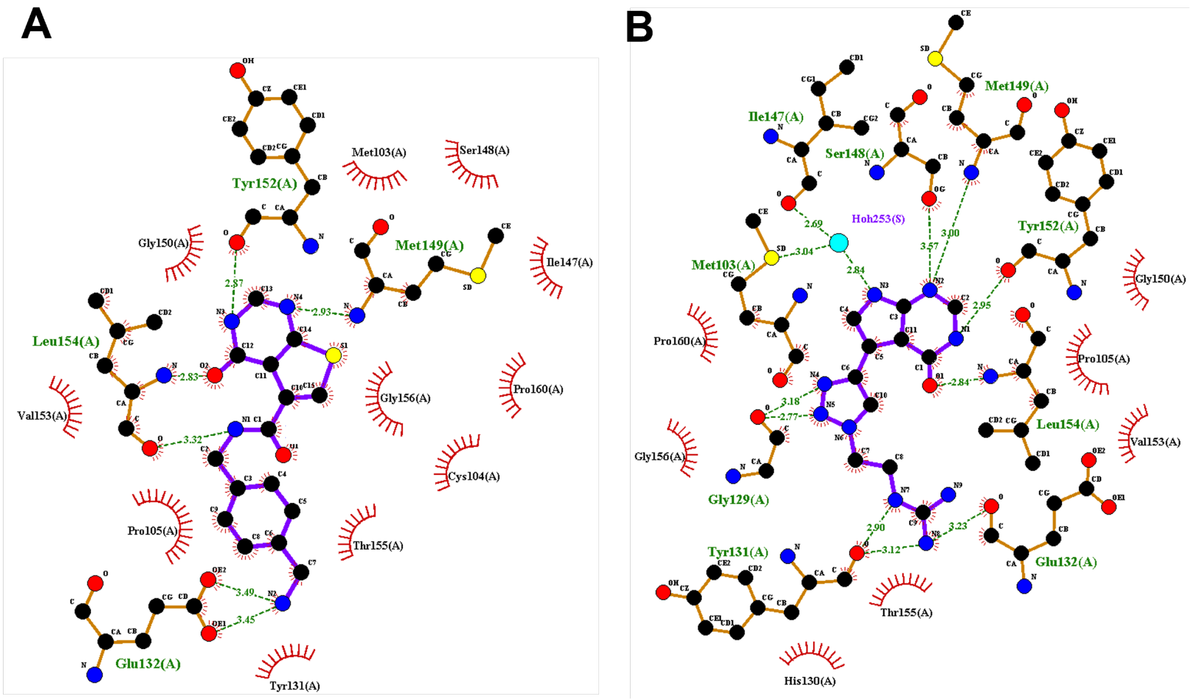

Figure S22. A schematic ligand-protein interaction diagram created with LigPlot+ for compound **2** (**A**) and compound **8h** (**B**). Intermolecular hydrogen bonds are given in green, hydrophobic interaction residues in red, and water as sky blue spheres.

Table S1: X-ray data collection refinement statistics of the structure co-crystalized with compound **2**.

**PDB entry 9SDV**	
Beamline	Diamond light source I03
Wavelength (Å)	0.976*
Resolution range(Å)	42.23 - 2.5 (2.75 - 2.5)
Space group	P 43 3 2
Unit cell	126.695 126.695 126.695 90 90 90
Total reflections	994338 (101554)*
Unique reflections	12564 (3062)
Multiplicity	78.90 (73.70)*
Completeness (%)	99.98 (99.93)
Mean I/sigma(I)	66.60 (13.10)*
Wilson B-factor (Å^2)	51.86
R-merge	0.06 (0.47)*
R-meas	0.06 (0.47)*
R-pim	0.01 (0.05)*
CC1/2	1.00 (0.99)*
Reflections used in refinement	12564 (3062)
Reflections used for R-free	608 (145)
R-work	0.2226 (0.2594)
R-free	0.2806 (0.3403)
Number of non-hydrogen atoms	1909
macromolecules	1825
Ligands	26
Solvent	58
Protein residues	231
RMS(bonds) (Å)	0.010
RMS(angles) (°)	1.13
Ramachandran favored (%)	95.15
Ramachandran allowed (%)	4.85
Ramachandran outliers (%)	0.00
Rotamer outliers (%)	0.52
Clashscore	8.08
Average B-factor (Å^2)	75.54
Macromolecules (Å^2)	76.10
Ligands (Å^2)	72.08
Solvent (Å^2)	59.51

Statistics for the highest resolution shell are shown in parentheses. * shows data from derived from ccp4.

Table S2: X-ray data collection refinement statistics of the structure co-crystalized with **8h**.

**PDB entry 9SDW**	
Beamline	Diamond light source I03
Wavelength (Å)	0.976*
Resolution range(Å)	44.86 - 2.6 (2.86 - 2.6)
Space group	P 43 3 2
Unit cell	126.875 126.875 126.875 90 90 90
Total reflections	899457 (112484)*
Unique reflections	11266 (2734)
Multiplicity	79.60 (83.90)*
Completeness (%)	99.97 (99.93)
Mean I/sigma(I)	44.70 (7.00)*
Wilson B-factor (Å^2)	59.97
R-merge	0.10 (0.97)*
R-meas	0.10 (0.98)*
R-pim	0.01 (0.11)*
CC1/2	1.00 (0.97)*
Reflections used in refinement	11266 (2734)
Reflections used for R-free	566 (140)
R-work	0.2275 (0.2534)
R-free	0.2912 (0.3343)
Number of non-hydrogen atoms	1883
macromolecules	1832
Ligands	21
Solvent	30
Protein residues	230
RMS(bonds) (Å)	0.007
RMS(angles) (°)	1.00
Ramachandran favored (%)	97.35
Ramachandran allowed (%)	2.65
Ramachandran outliers (%)	0.00
Rotamer outliers (%)	3.52
Clashscore	14.84
Average B-factor (Å^2)	79.94
Macromolecules (Å^2)	80.47
Ligands (Å^2)	66.73
Solvent (Å^2)	56.82

Statistics for the highest resolution shell are shown in parentheses. * shows data from derived from ccp4.

Physicochemical properties of the azide-library

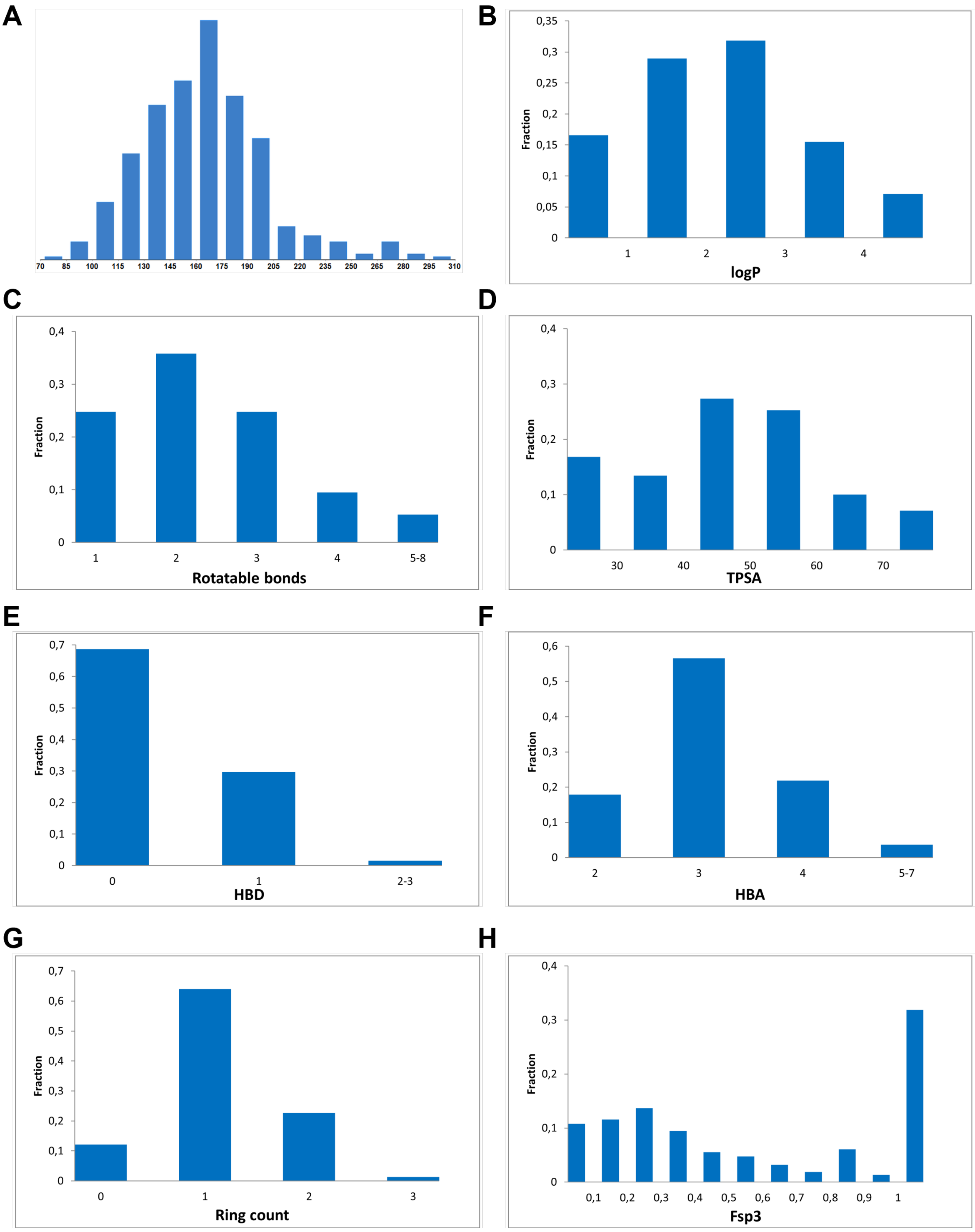

Figure S23. Overview of the physicochemical properties of the azide library purchase from Enamine.net. (**A**) Molecular weight [g/mol], (**B**) log P value, (**C**) Number of rotatable bonds, (**D**) Toplogical polar surface area [Å²], (**E**) Number of H-bond donors, (**F**) Number of H-bond acceptors, (**G**) Number of rings, (**H**) Fraction of sp^3 atoms.

Docking poses compounds 2 and 8b–e,h

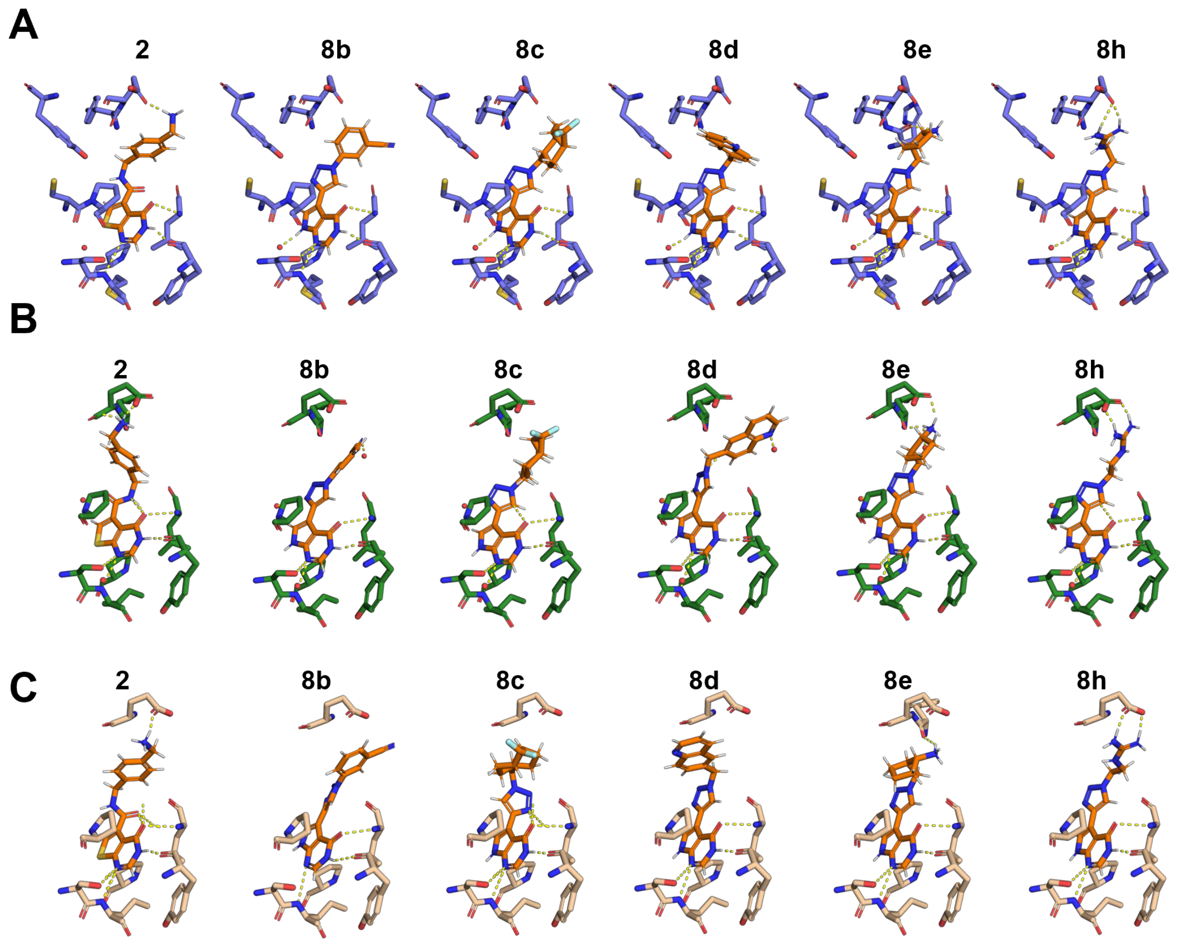

Figure S24: Glide docking poses for compounds **2** and **8b**–**e,h** after docking to (**A**) *Staphylococcus aureus* (PDB: 9SDW, purple carbons), (**B**) *Haemophilus influenzae* (PDB: 4MCC; green carbons), and (**C**) *Escherichia coli* (PDB: 1P9P, sand carbons). Ligand carbons are shown as sticks in orange, selected amino acids are shown as sticks, and water is shown as red spheres.

Table S3: Glide scores for compounds **2** and **8b–e,h** after docking to *Haemophilus influenzae* (PDB: 4MCC), *Escherichia coli* (PDB: 1P9P), and *Staphylococcus aureus* (PDB: 9SDW).

	**2**	**8b**	**8c**	**8d**	**8e**	**8h**
Haemophilus influenzae (PDB: 4MCC)	-13.44	-10.78	-11.03	-9.73	-10.74	-11.91
Escherichia coli (PDB: 1P9P)	-12.18	-8.28	-10.61	-11.32	-11.73	-11.38
Staphylococcus aureus (PDB: 9SDW)	-7.95	-11.623	-11.27	-11.80	-12.86	-12.74

NMR and LC/MS Spectra

Compound 2

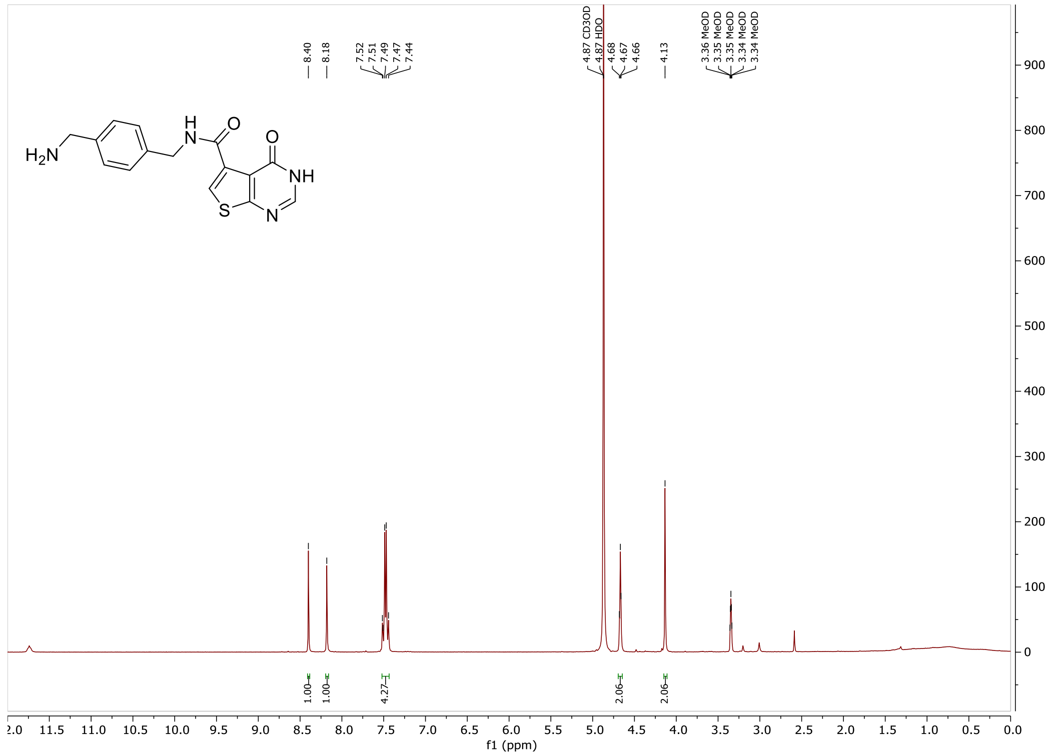

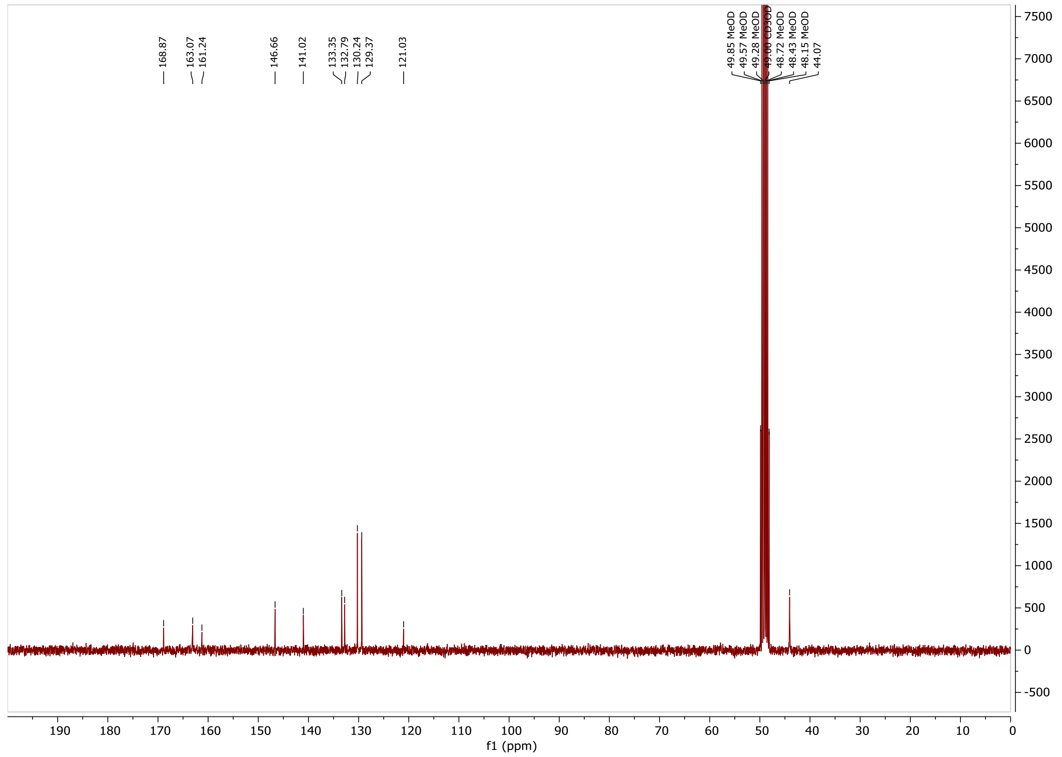

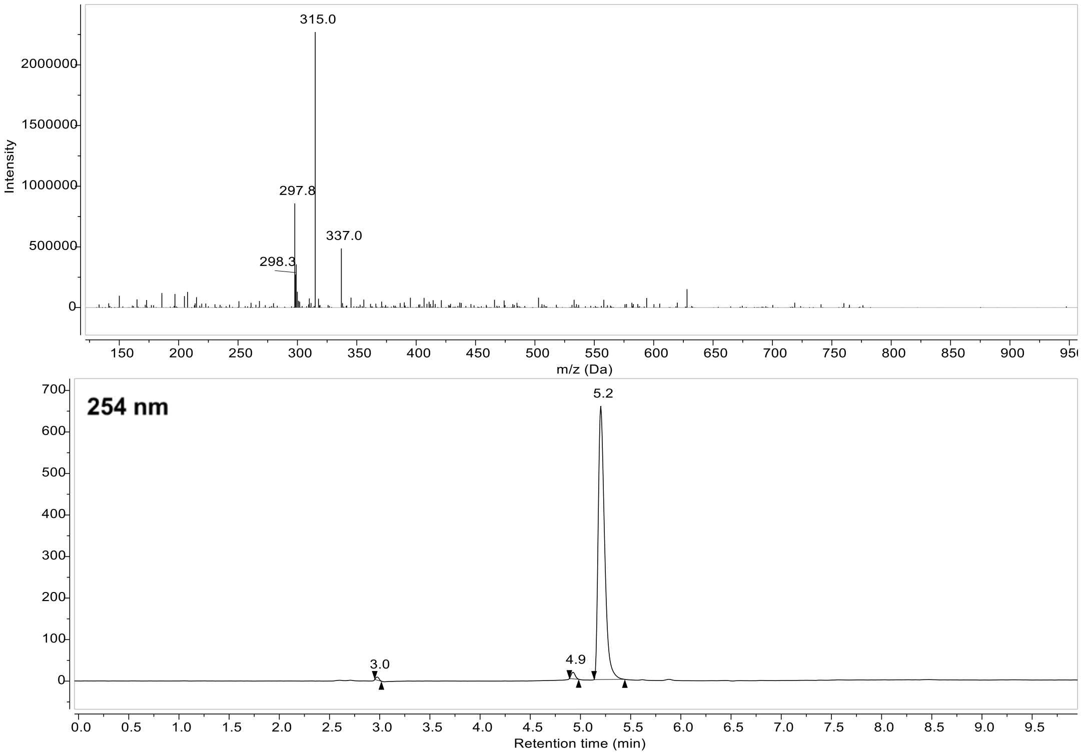
315.0
297.8
337.0
298.3
Intensity
m/z (Da)
254 nm
5.2
3.0
4.9
Retention time (min)

Compound 6

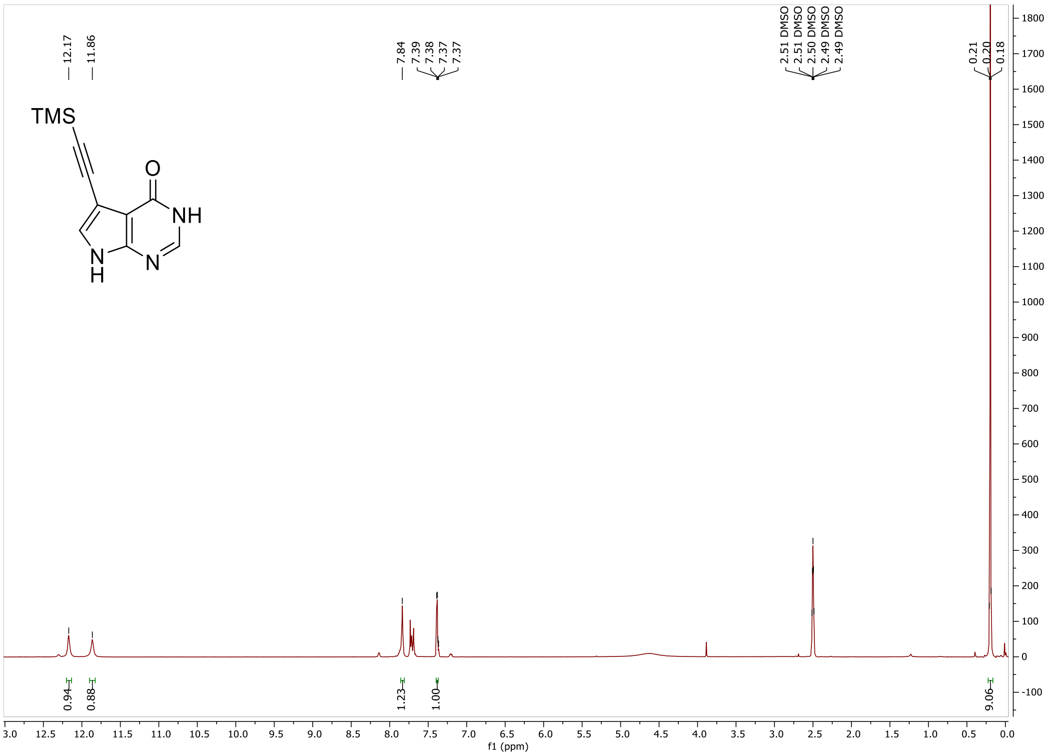
12.17
11.86
7.84
7.39
7.38
7.37
7.37
2.51 DMSO
2.51 DMSO
2.50 DMSO
2.49 DMSO
2.49 DMSO
0.21
0.20
0.18
TMS
O
NH
N
N
H
0.94
0.88
1.23
1.00
9.06
f1 (ppm)

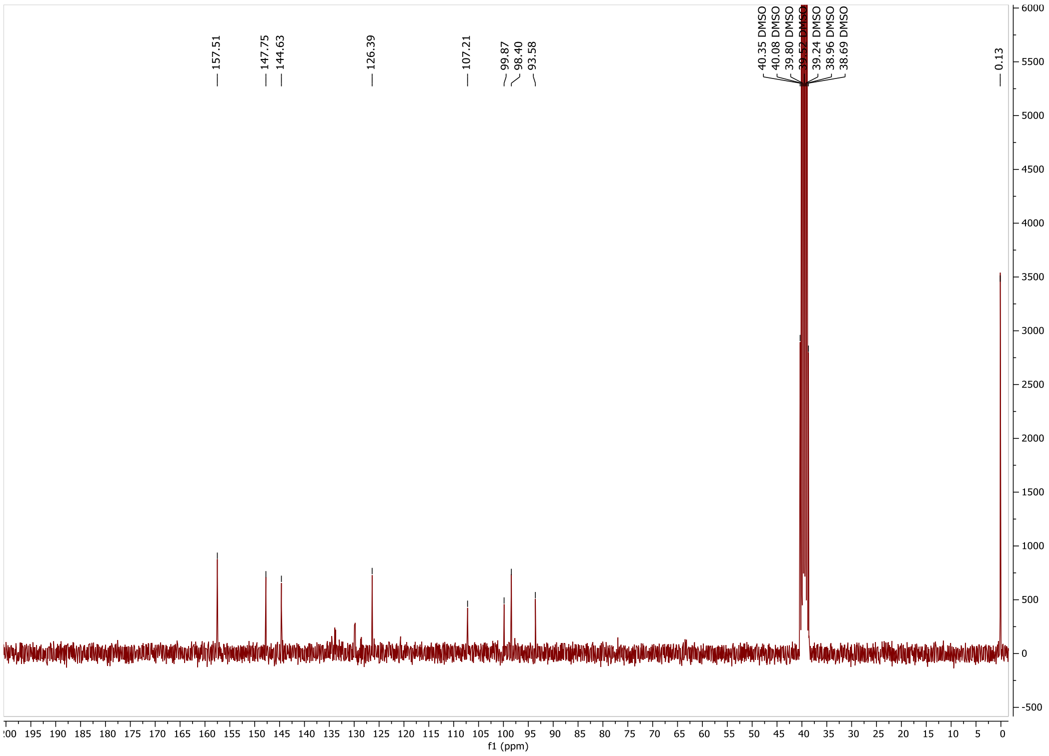
157.51
147.75
144.63
126.39
107.21
99.87
98.40
93.58
40.35 DMSO
40.08 DMSO
39.80 DMSO
39.24 DMSO
38.96 DMSO
38.69 DMSO
0.13
f1 (ppm)

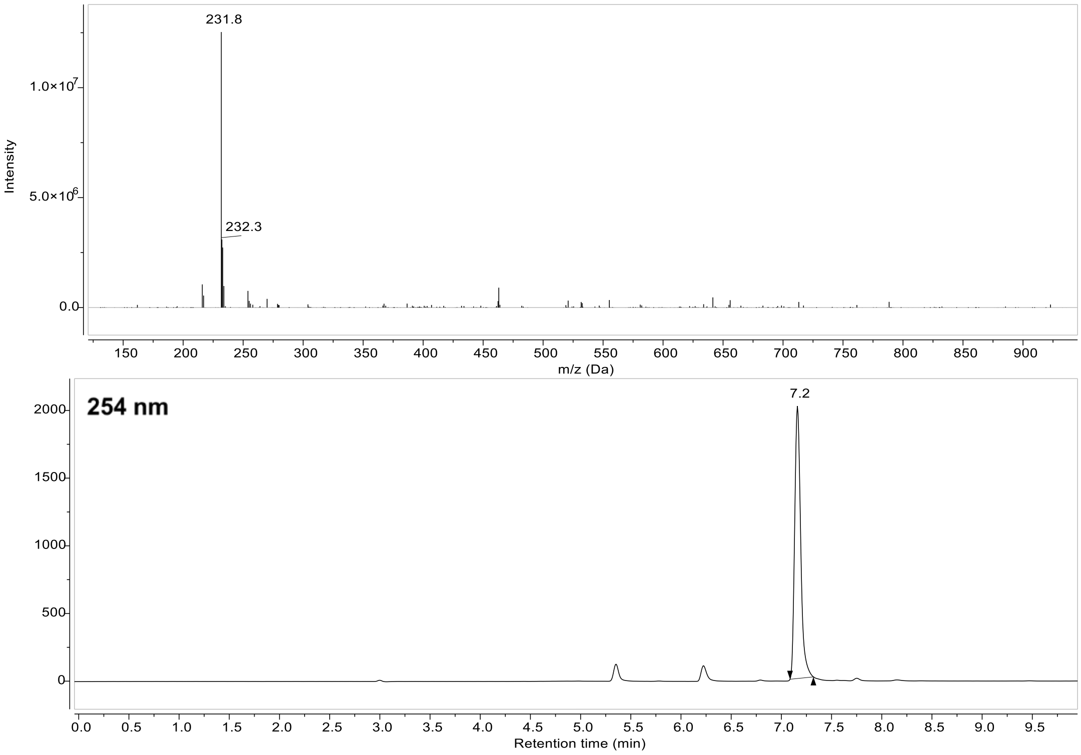

231.8
232.3
1.0×10^7
5.0×10^6
0.0
Intensity
150 200 250 300 350 400 450 500 550 600 650 700 750 800 850 900
m/z (Da)
254 nm
7.2
2000
1500
1000
500
0
0.0 0.5 1.0 1.5 2.0 2.5 3.0 3.5 4.0 4.5 5.0 5.5 6.0 6.5 7.0 7.5 8.0 8.5 9.0 9.5
Retention time (min)

Compound 7

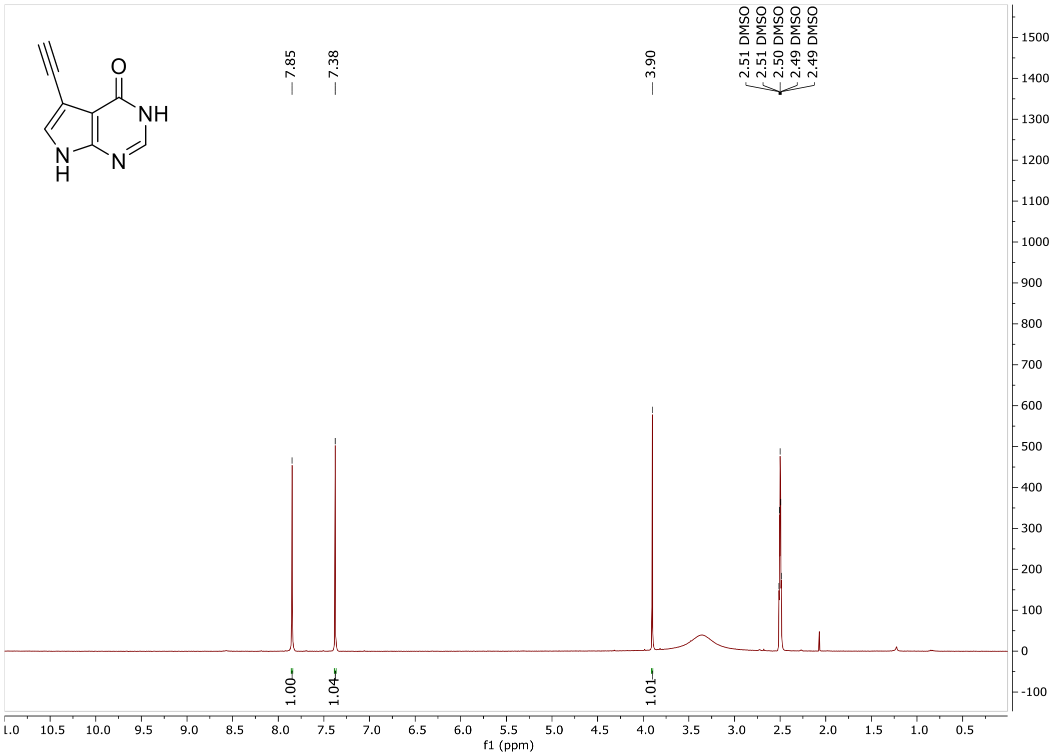

7.85
7.38
3.90
2.51 DMSO
2.51 DMSO
2.50 DMSO
2.49 DMSO
2.49 DMSO
1.00
1.04
1.01
f1 (ppm)

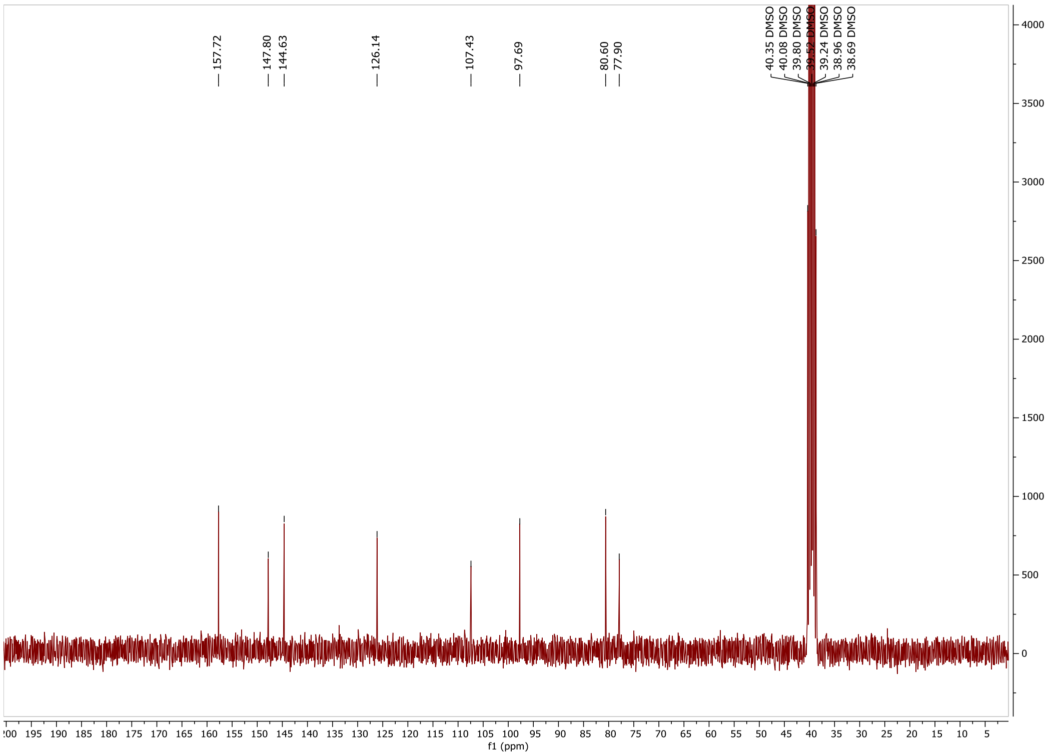

157.72
147.80
144.63
126.14
107.43
97.69
80.60
77.90
40.35 DMSO
40.08 DMSO
39.80 DMSO
39.52 DMSO
39.24 DMSO
38.96 DMSO
38.69 DMSO
f1 (ppm)

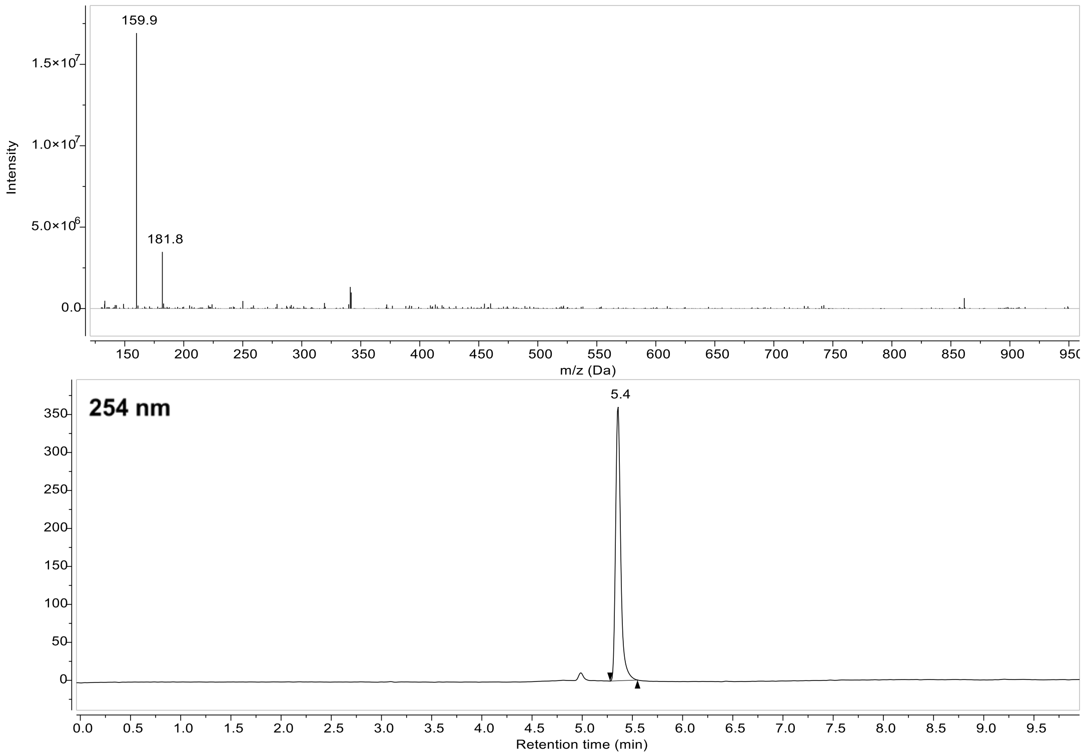
159.9
181.8
Intensity
1.5×10^7
1.0×10^7
5.0×10^6
0.0
150 200 250 300 350 400 450 500 550 600 650 700 750 800 850 900 950
m/z (Da)
254 nm
5.4
350 300 250 200 150 100 50 0
0.0 0.5 1.0 1.5 2.0 2.5 3.0 3.5 4.0 4.5 5.0 5.5 6.0 6.5 7.0 7.5 8.0 8.5 9.0 9.5
Retention time (min)

Compound 10

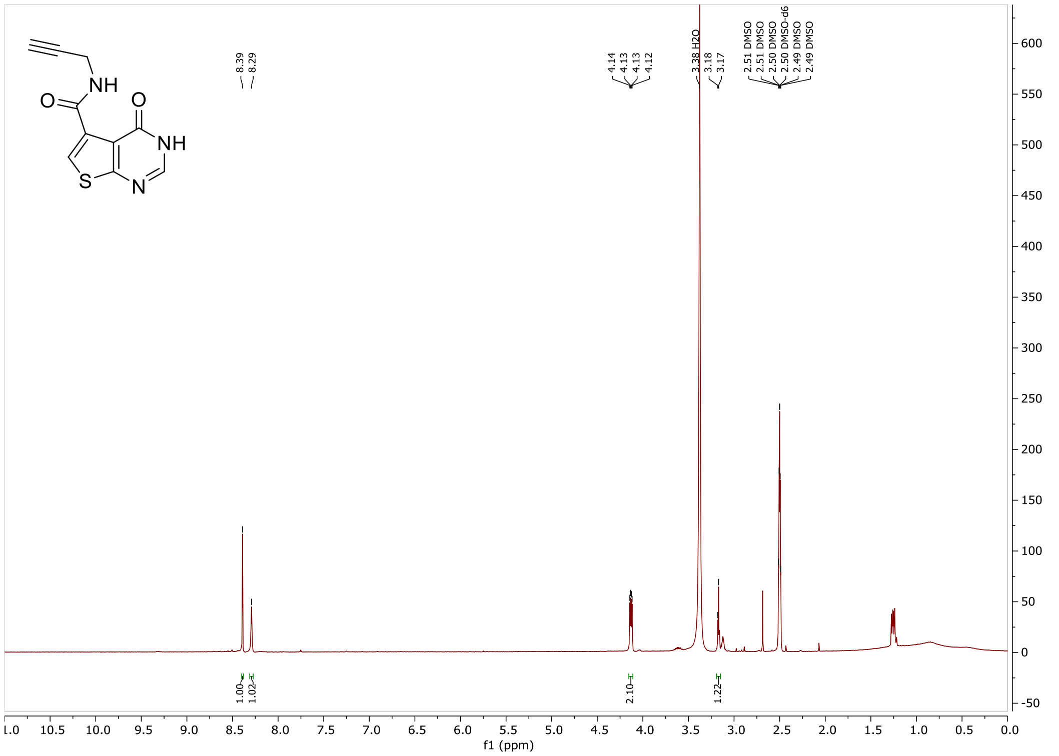

NH
O
O
NH
S
N
8.39
8.29
4.14
4.13
4.13
4.12
3.38 H2O
3.18
3.17
2.51 DMSO
2.51 DMSO
2.50 DMSO
2.50 DMSO-d6
2.49 DMSO
2.49 DMSO
1.00
1.02
2.10
1.22
f1 (ppm)

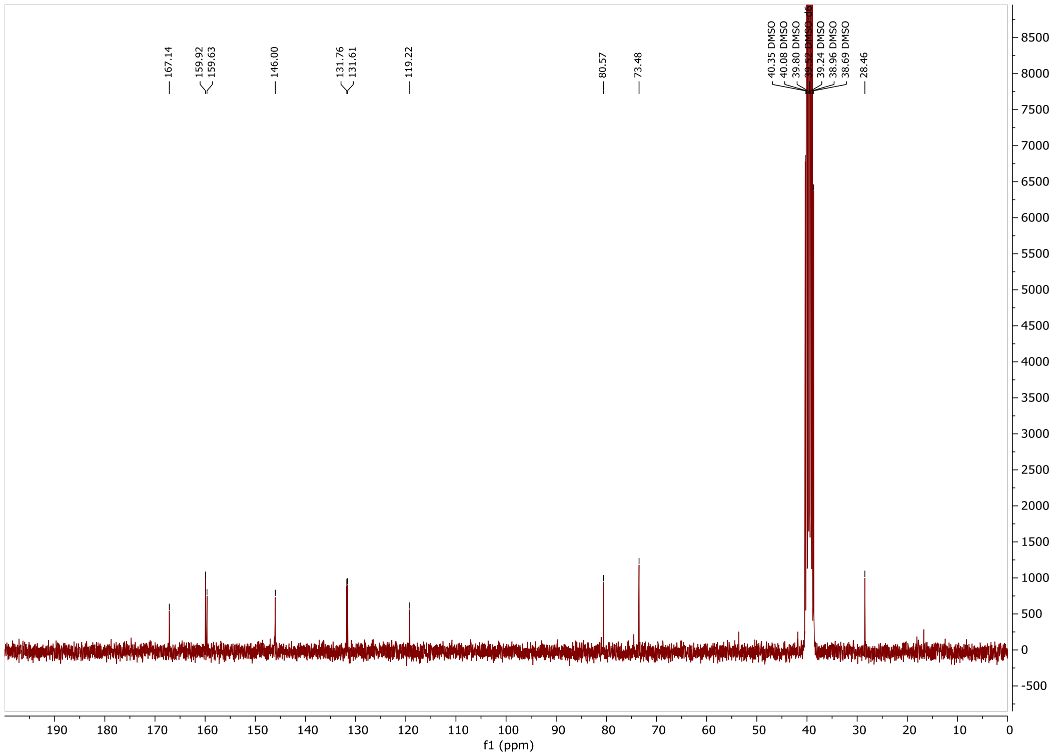

167.14
159.92
159.63
146.00
131.76
131.61
119.22
80.57
73.48
40.35 DMSO
40.08 DMSO
39.80 DMSO
39.24 DMSO
38.96 DMSO
38.69 DMSO
28.46
f1 (ppm)

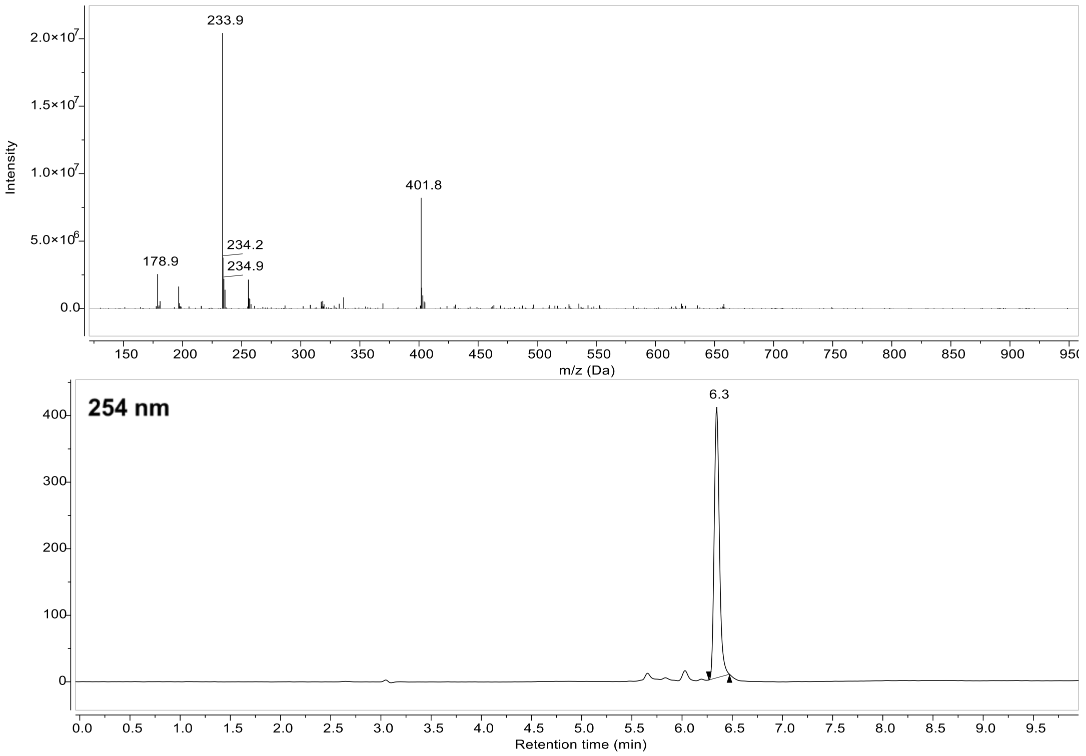
233.9
401.8
178.9
234.2
234.9
Intensity
m/z (Da)
254 nm
6.3
Retention time (min)

Compound 12

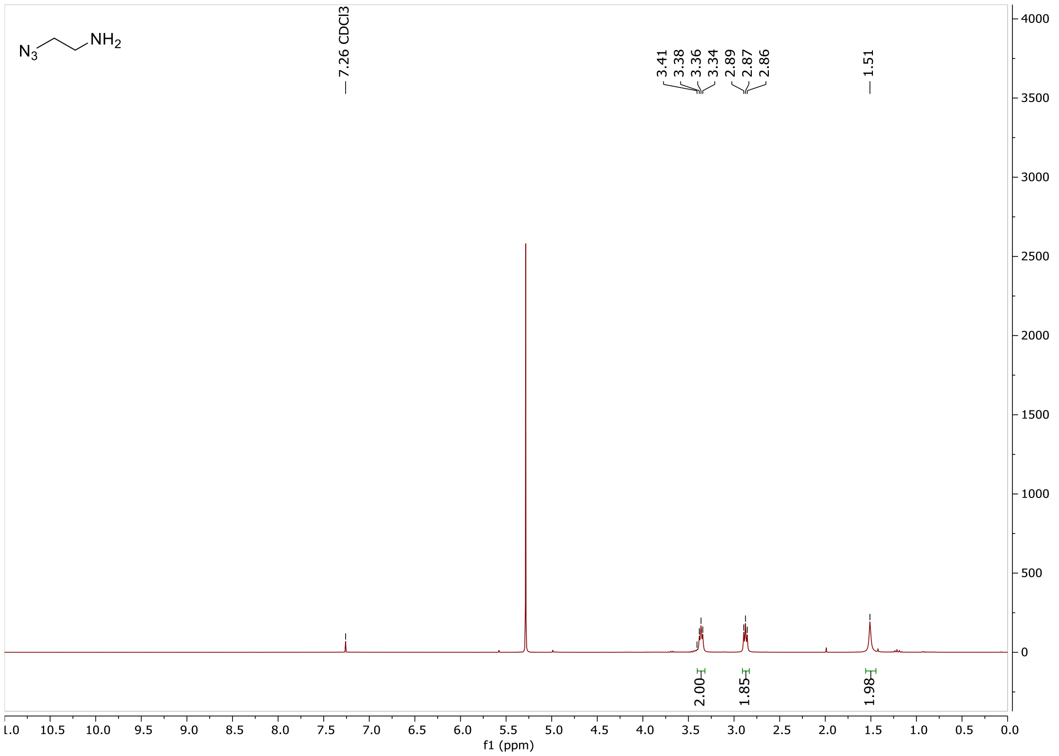

N3 NH2
7.26 CDCl3
3.41
3.38
3.36
3.34
2.89
2.87
2.86
1.51
2.00
1.85
1.98
f1 (ppm)

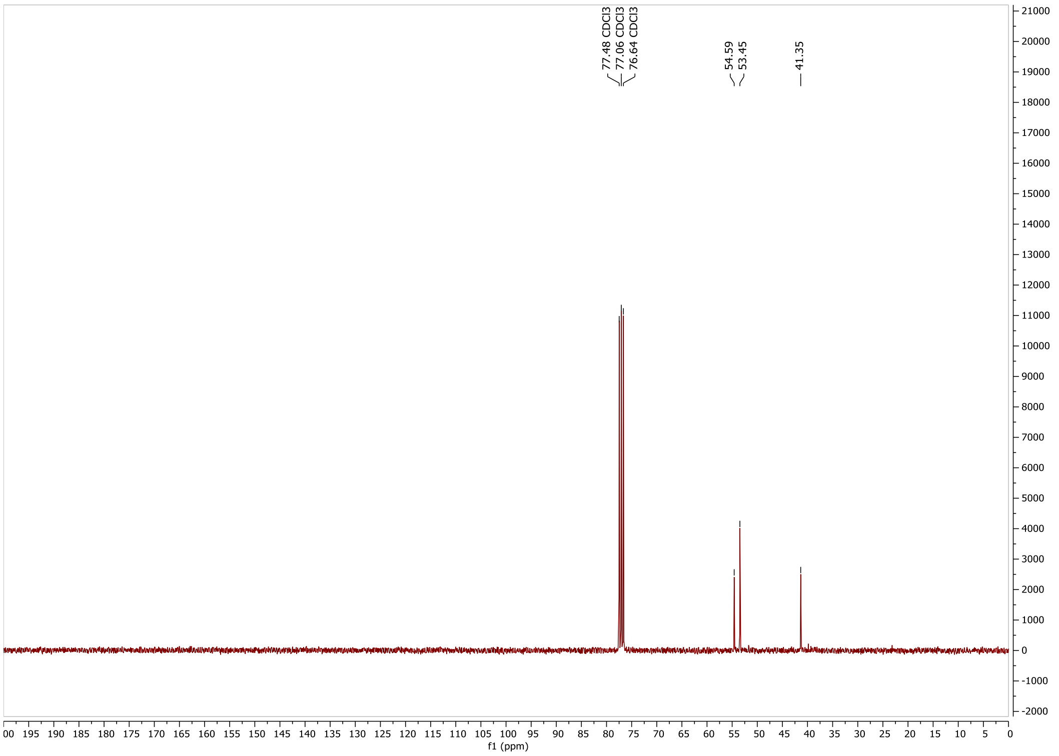

77.48 CDCl3
77.06 CDCl3
76.64 CDCl3
54.59
53.45
41.35
f1 (ppm)

Compound 15

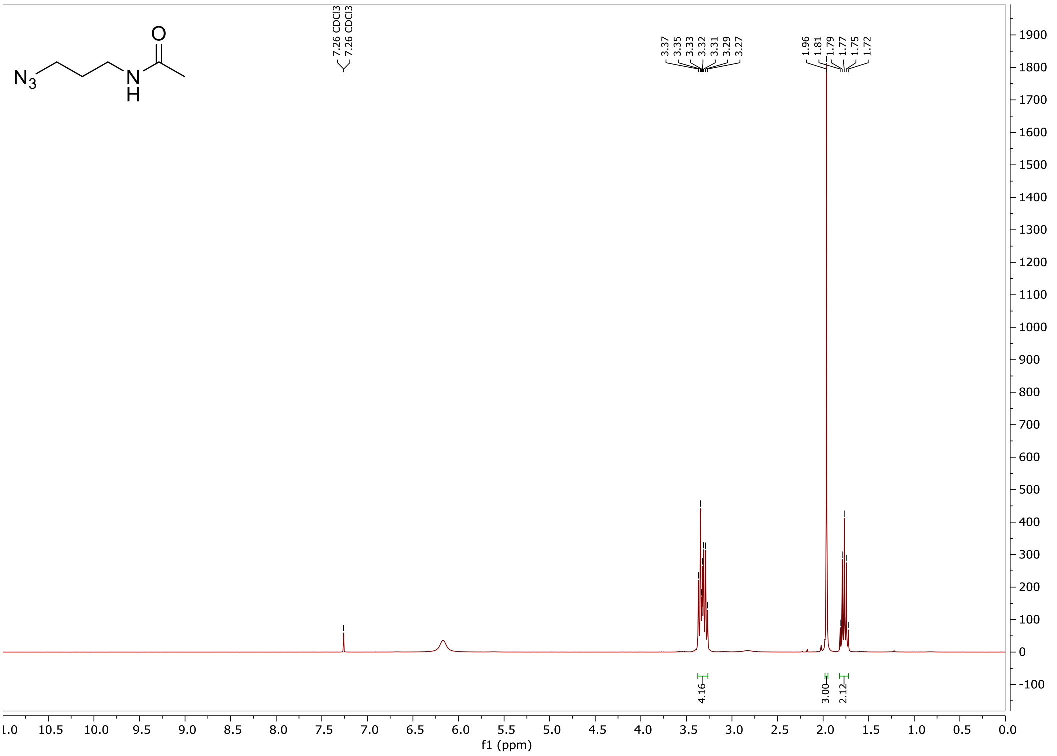
7.26 CDCl3
7.26 CDCl3
3.37
3.35
3.33
3.32
3.31
3.29
3.27
1.96
1.81
1.79
1.77
1.75
1.72
4.16
3.00
2.12
f1 (ppm)

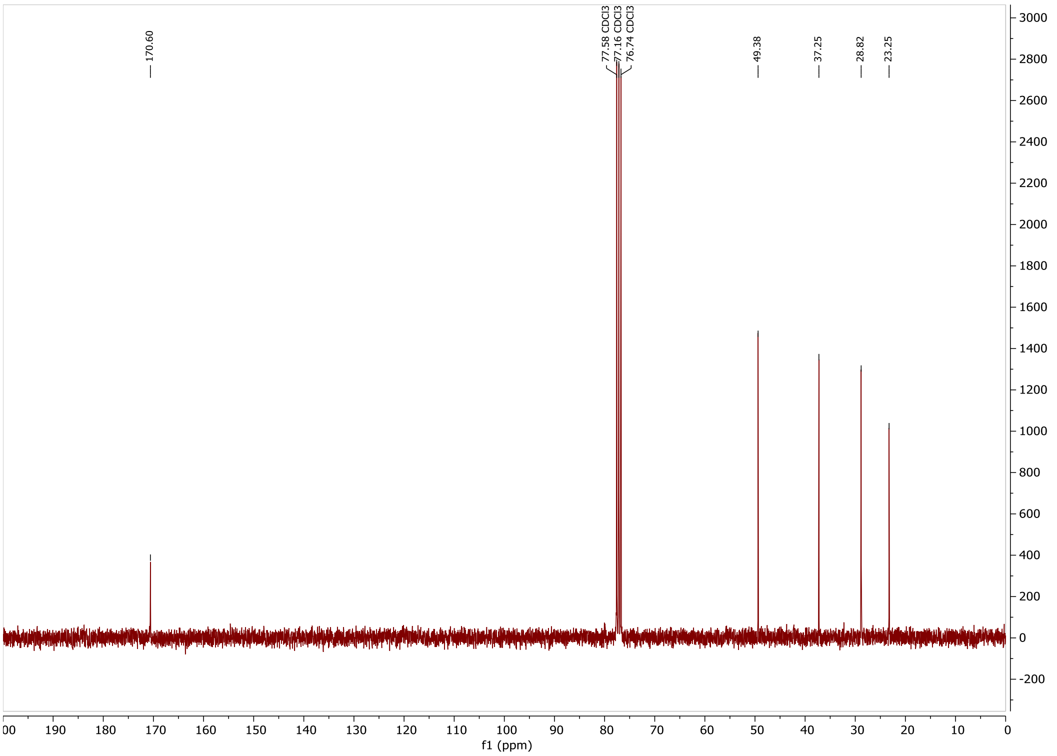
170.60
77.58 CDCl3
77.16 CDCl3
76.74 CDCl3
49.38
37.25
28.82
23.25
f1 (ppm)

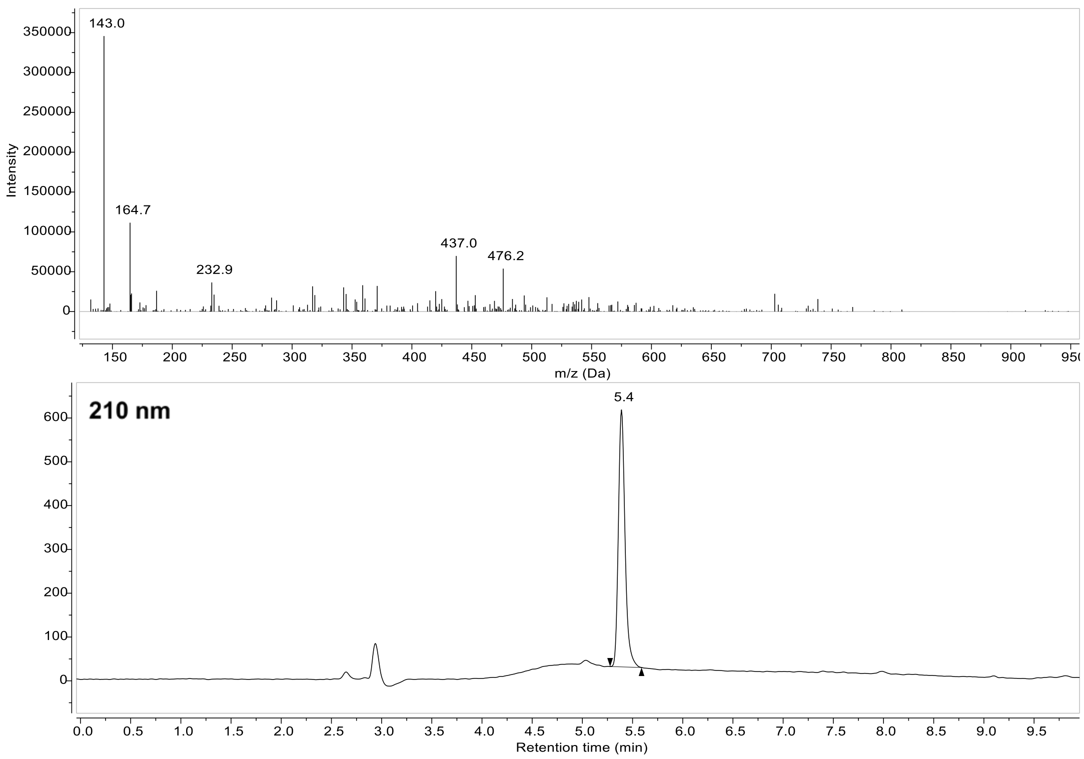
143.0
164.7
232.9
437.0
476.2
Intensity
m/z (Da)
210 nm
5.4
Retention time (min)

Compound 8a (*in situ*)

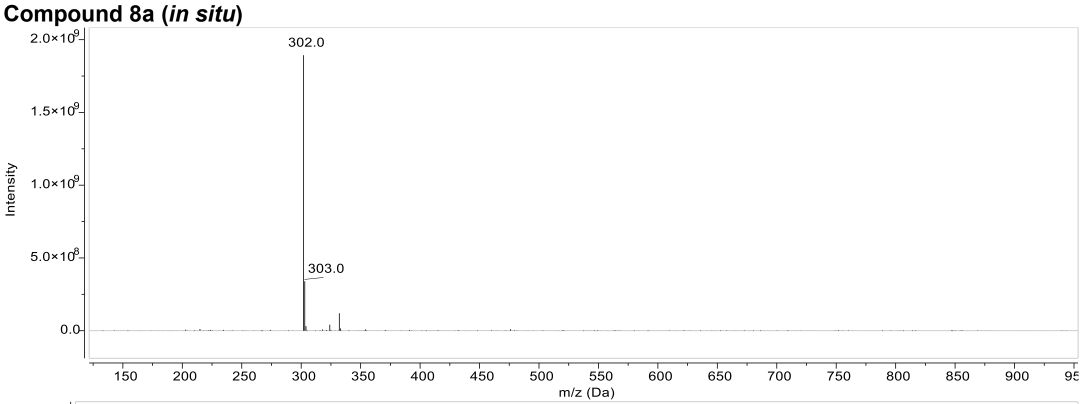

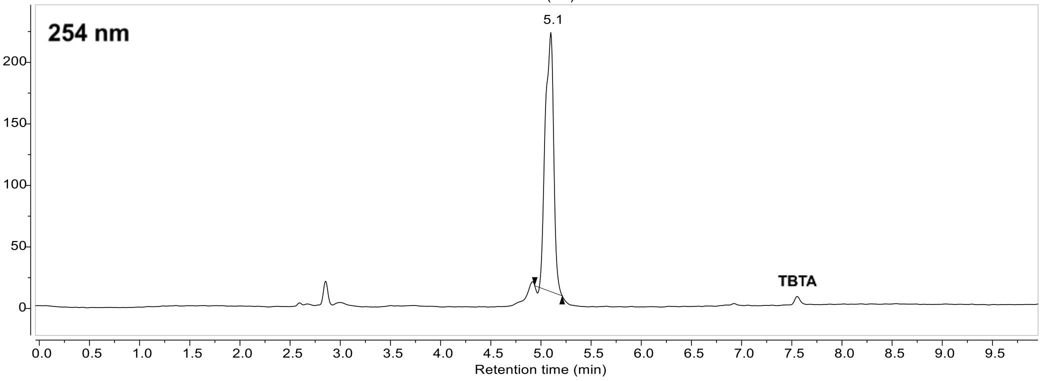

Compound 8a (preparative, meaning chromatographically purified)

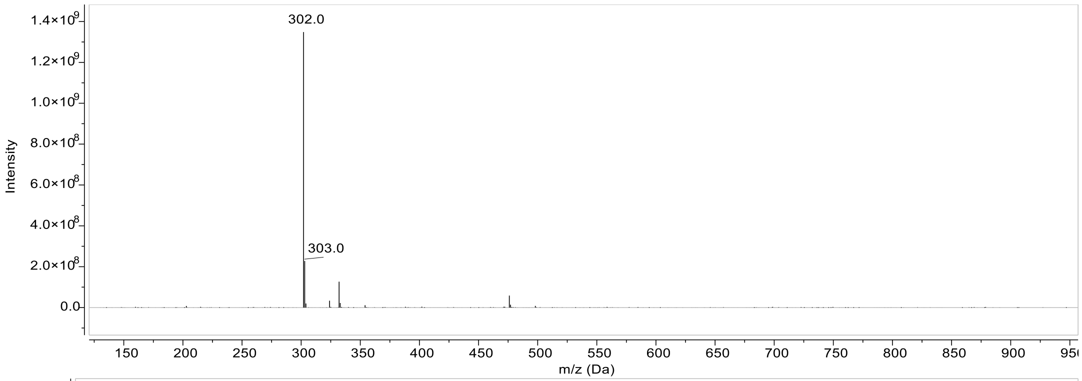

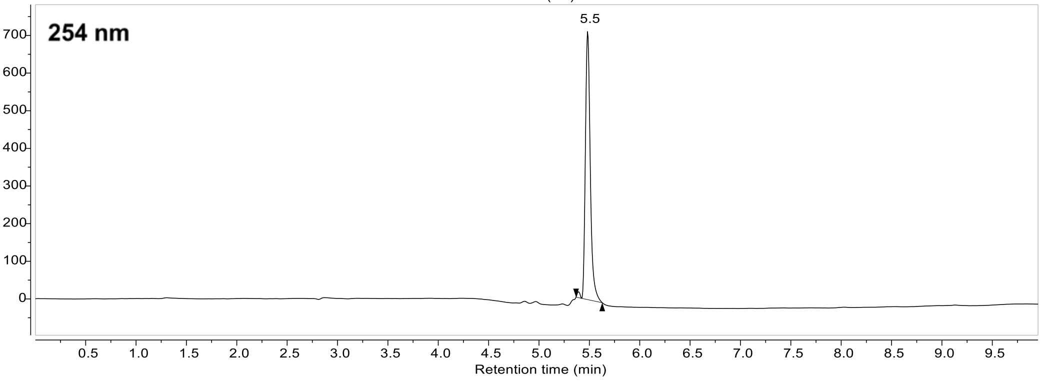

Compound 14

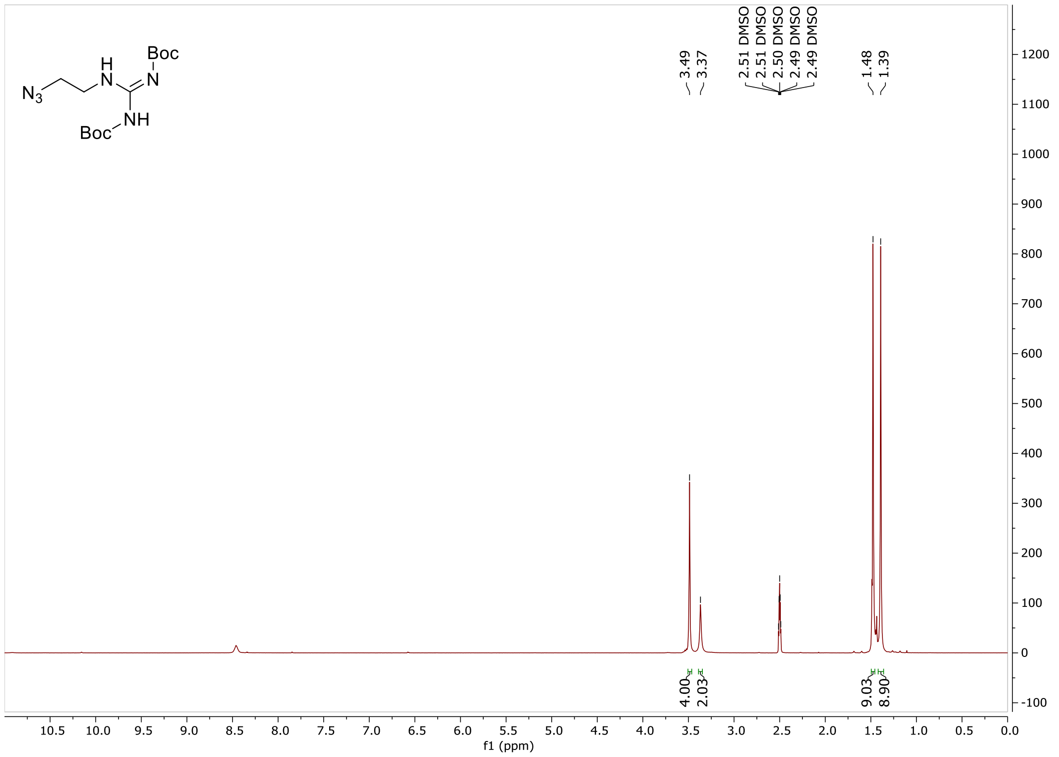

Boc
H
N
N
N3
NH
Boc
3.49
3.37
2.51 DMSO
2.51 DMSO
2.50 DMSO
2.49 DMSO
2.49 DMSO
1.48
1.39
4.00
2.03
9.03
8.90
f1 (ppm)

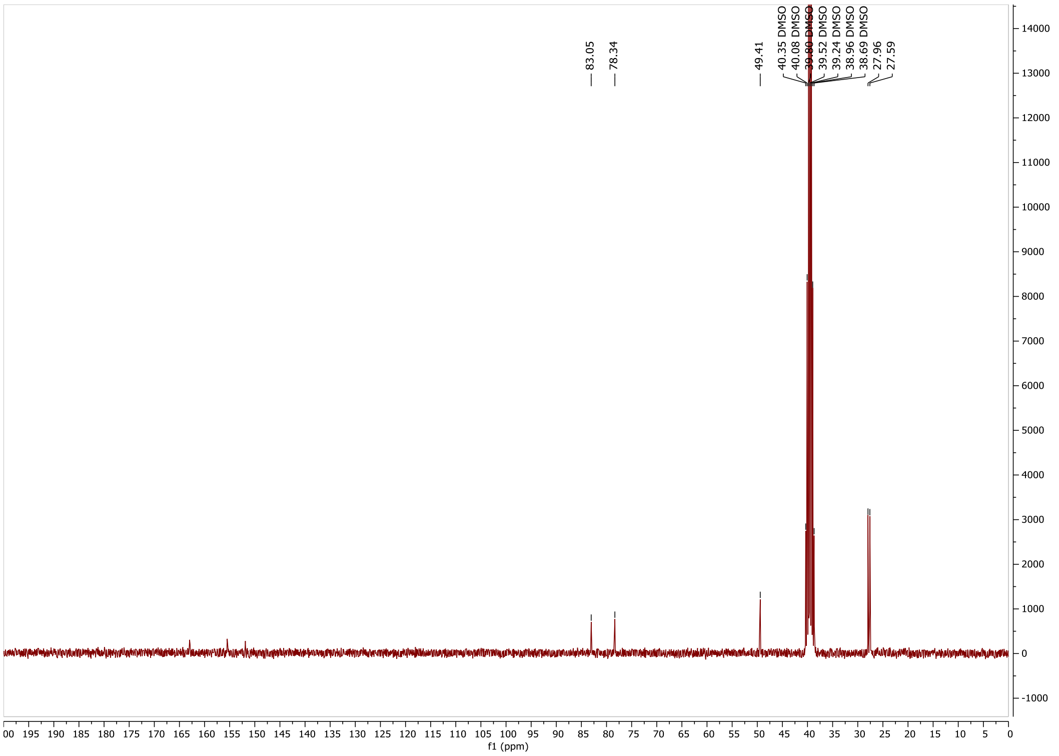

83.05
78.34
49.41
40.35 DMSO
40.08 DMSO
39.80 DMSO
39.52 DMSO
39.24 DMSO
38.96 DMSO
38.69 DMSO
27.96
27.59
f1 (ppm)

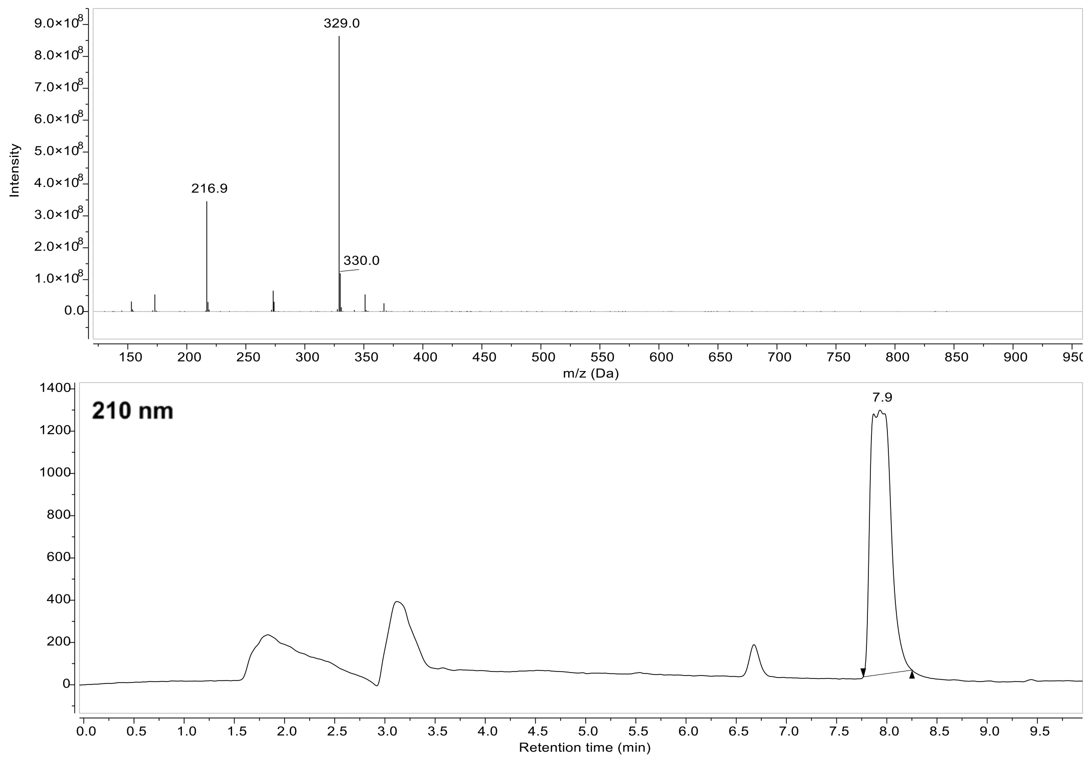
329.0
216.9
330.0
Intensity
m/z (Da)
210 nm
7.9
Retention time (min)

Compound 13

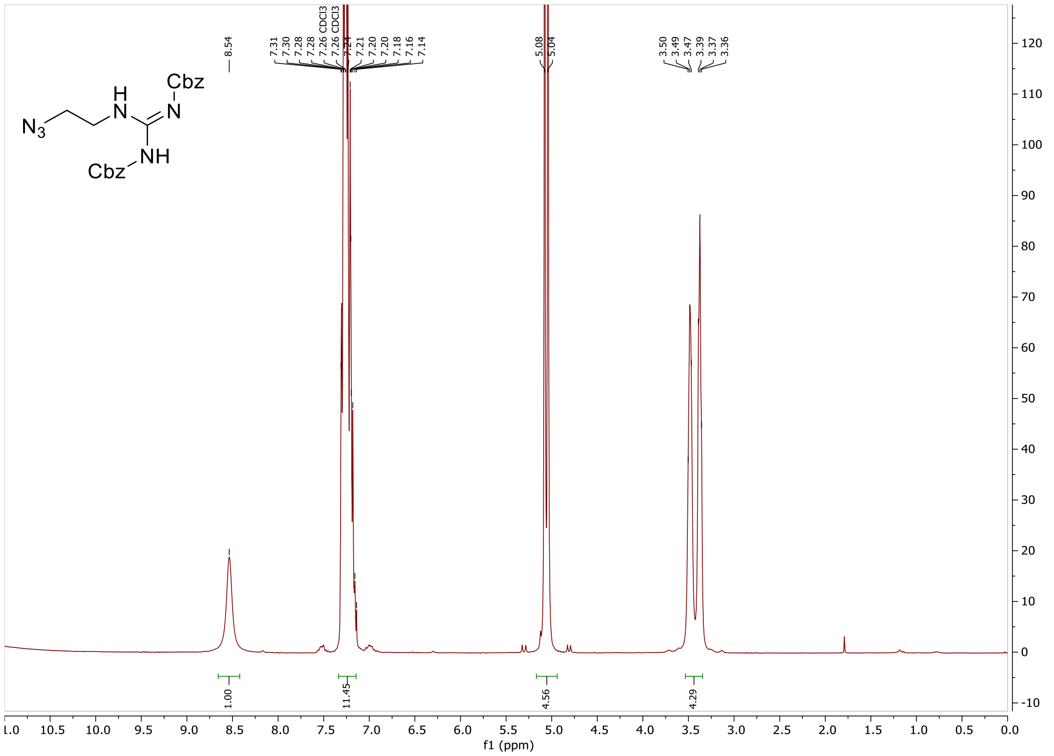

8.54
7.31
7.30
7.28
7.28
7.26 CDCl3
7.26 CDCl3
7.24
7.21
7.20
7.20
7.18
7.16
7.14
5.08
5.04
3.50
3.49
3.47
3.39
3.37
3.36
H
N
Cbz
N
N_3
NH
Cbz
1.00
11.45
4.56
4.29
f1 (ppm)

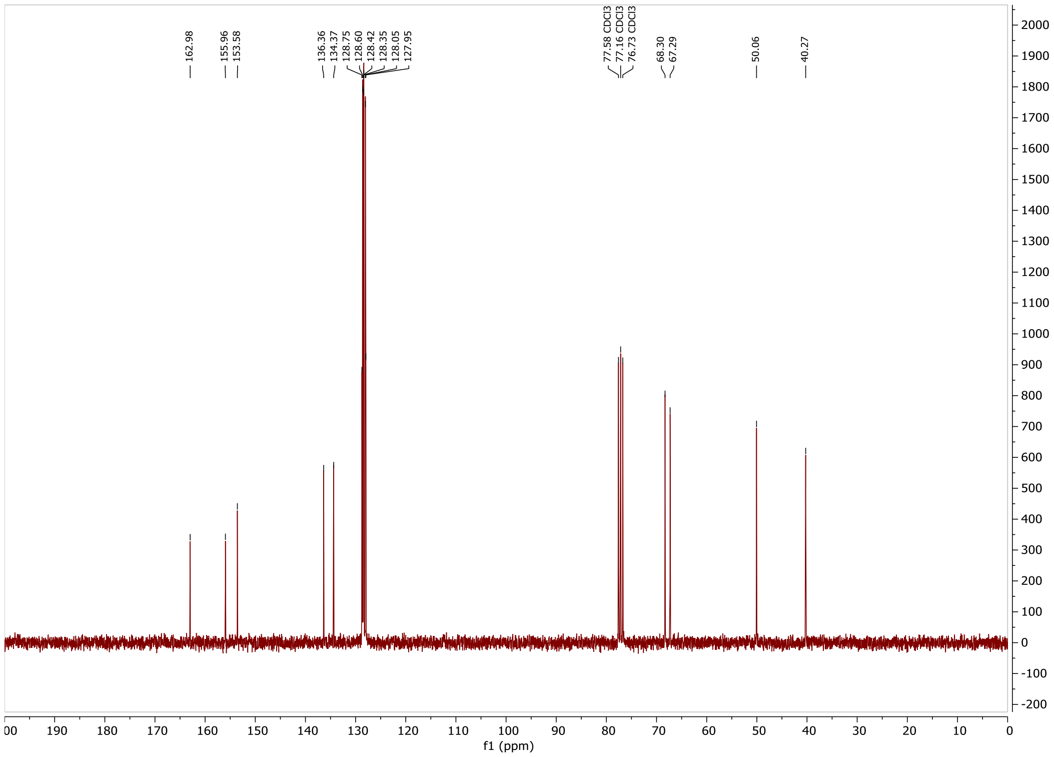

162.98
155.96
153.58
136.36
134.37
128.75
128.60
128.42
128.35
128.05
127.95
77.58 CDCl3
77.16 CDCl3
76.73 CDCl3
68.30
67.29
50.06
40.27
f1 (ppm)

Compound 8h:

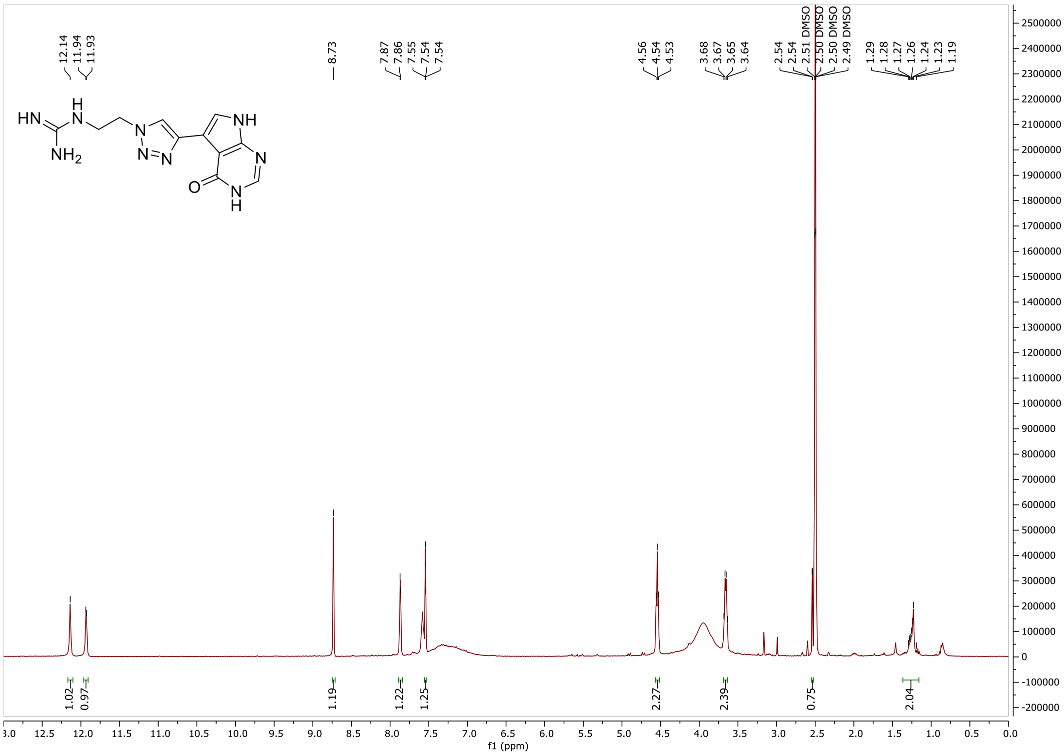

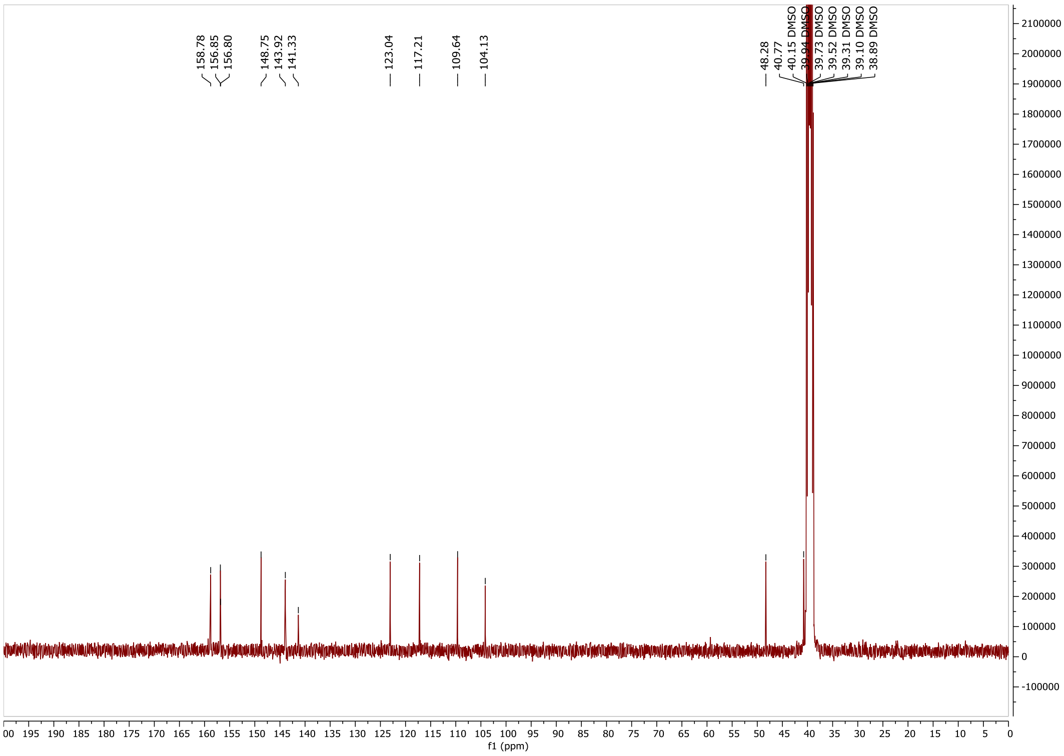

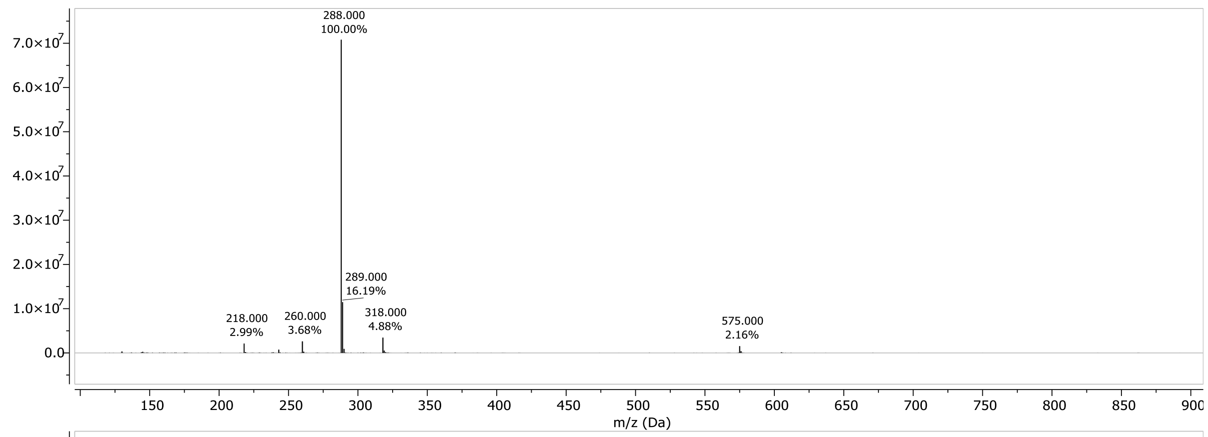
288.000
100.00%
289.000
16.19%
218.000
2.99%
260.000
3.68%
318.000
4.88%
575.000
2.16%
m/z (Da)

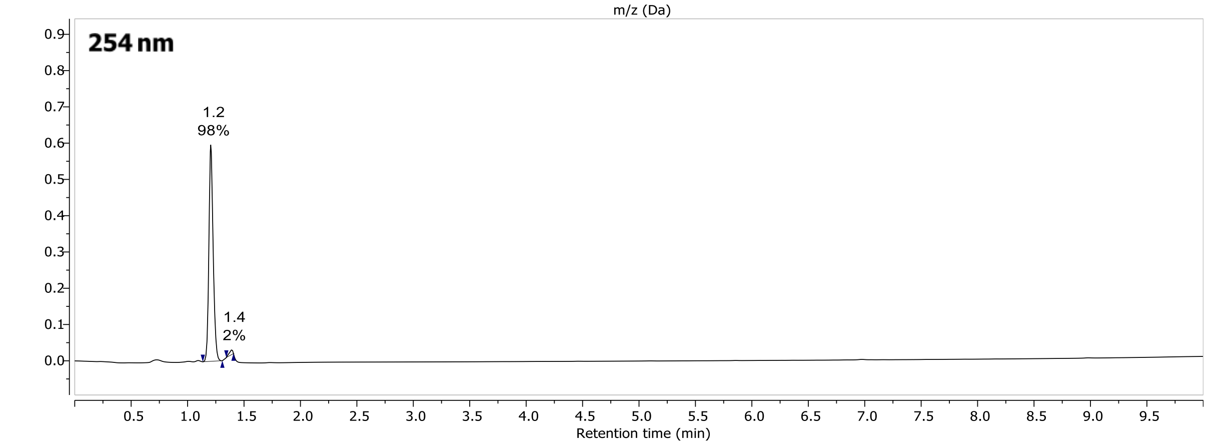
254 nm
1.2
98%
1.4
2%
Retention time (min)

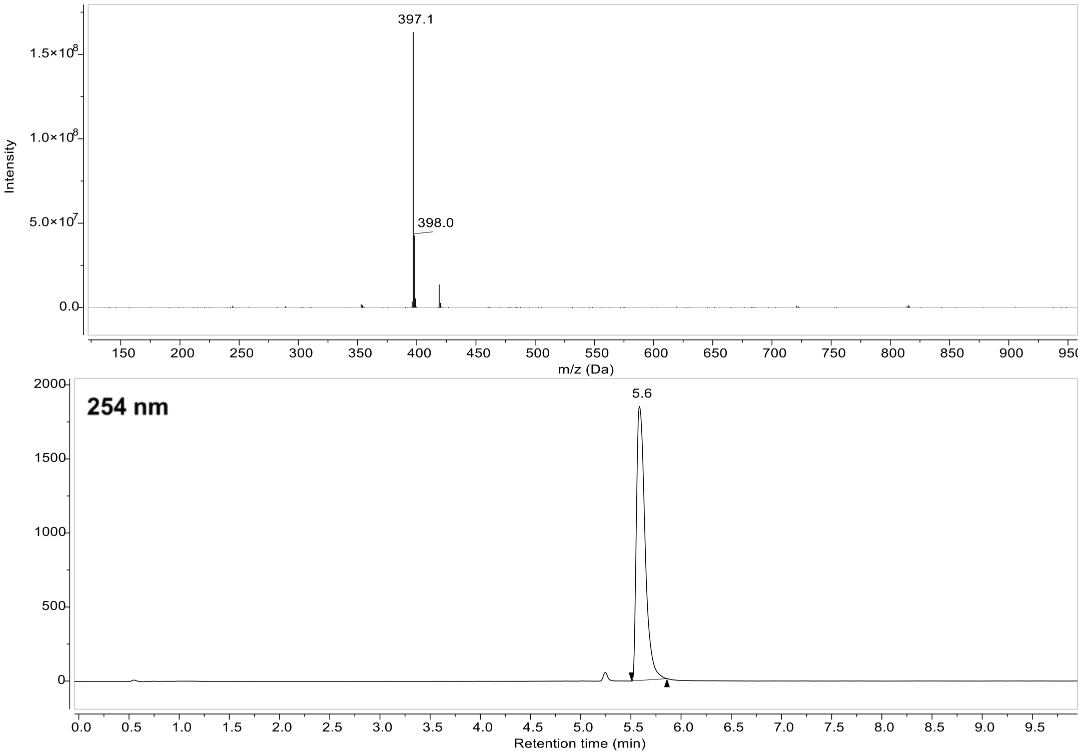
397.1
398.0
Intensity
1.5×10^8
1.0×10^8
5.0×10^7
0.0
150 200 250 300 350 400 450 500 550 600 650 700 750 800 850 900 950
m/z (Da)
254 nm
5.6
2000
1500
1000
500
0
0.0 0.5 1.0 1.5 2.0 2.5 3.0 3.5 4.0 4.5 5.0 5.5 6.0 6.5 7.0 7.5 8.0 8.5 9.0 9.5
Retention time (min)

Compound 8g

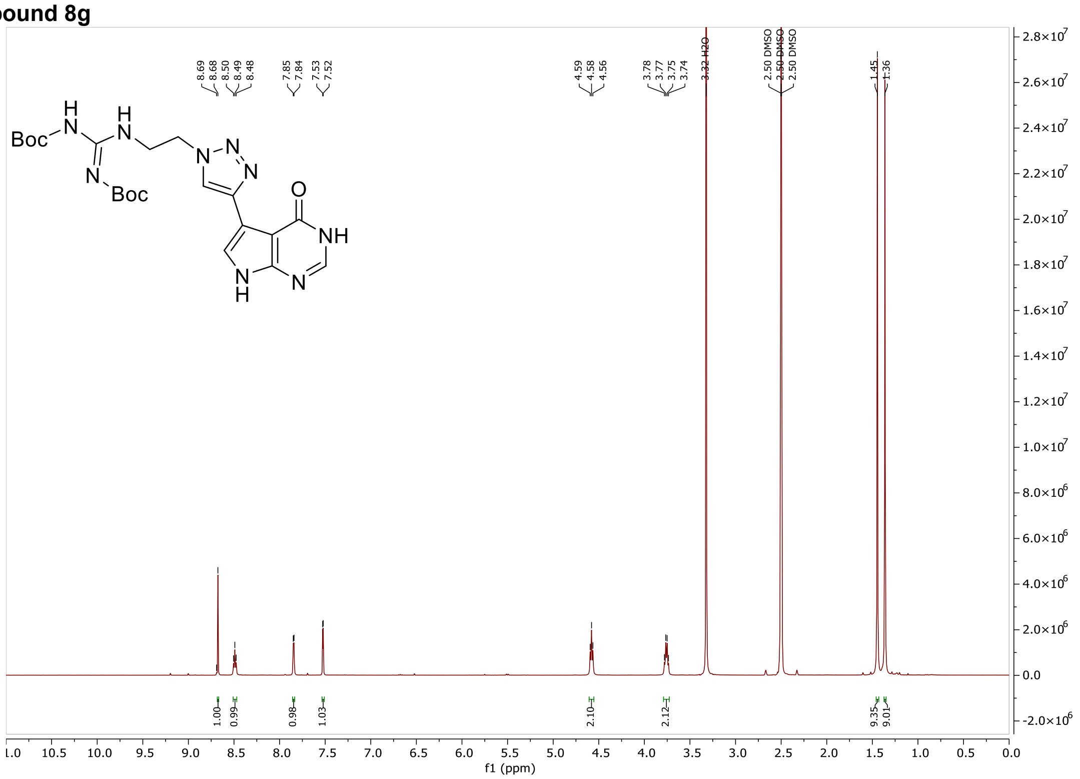
8.69
8.68
8.50
8.49
8.48
7.85
7.84
7.53
7.52
4.59
4.58
4.56
3.78
3.77
3.75
3.74
3.32 H2O
2.50 DMSO
2.50 DMSO
2.50 DMSO
1.45
1.36
1.00
0.99
0.98
1.03
2.10
2.12
9.35
9.01
f1 (ppm)

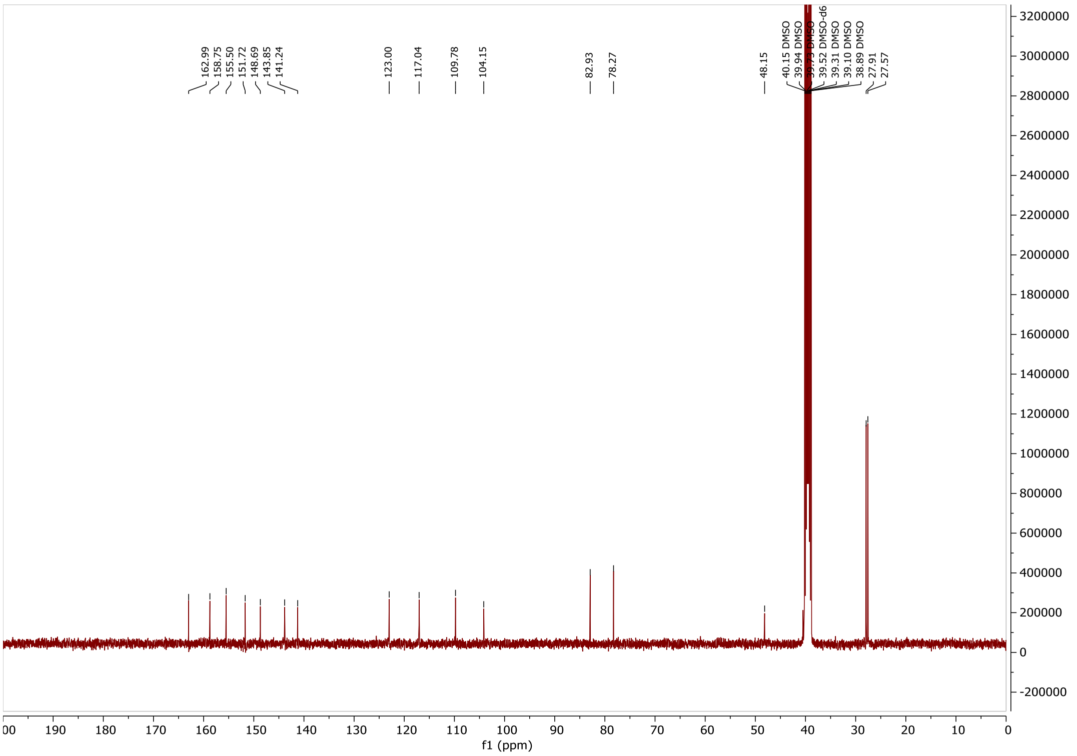
162.99
158.75
155.50
151.72
148.69
143.85
141.24
123.00
117.04
109.78
104.15
82.93
78.27
48.15
40.15 DMSO
39.94 DMSO
39.52 DMSO-d6
39.31 DMSO
39.10 DMSO
38.89 DMSO
27.91
27.57
f1 (ppm)

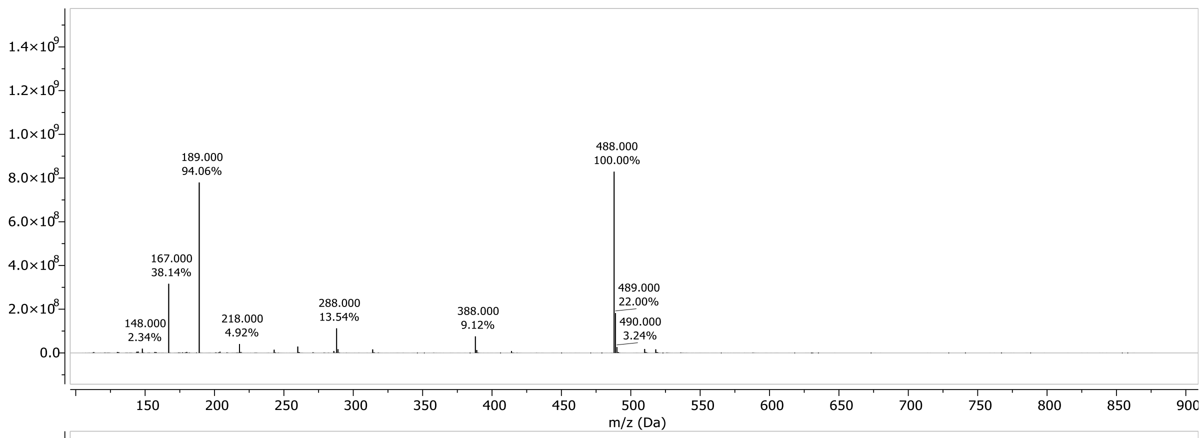

148.000
2.34%
167.000
38.14%
189.000
94.06%
218.000
4.92%
288.000
13.54%
388.000
9.12%
488.000
100.00%
489.000
22.00%
490.000
3.24%
m/z (Da)

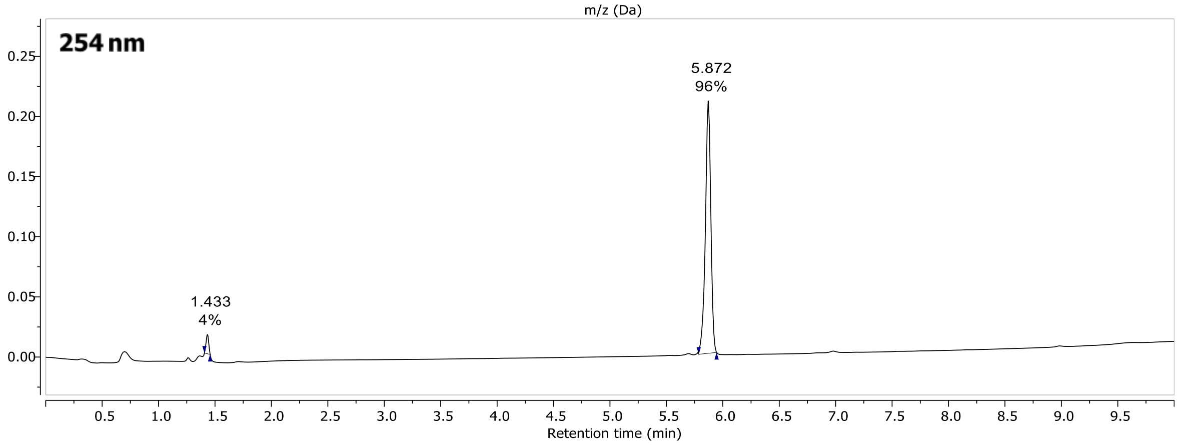

254 nm
1.433
4%
5.872
96%
Retention time (min)

Compound 8f

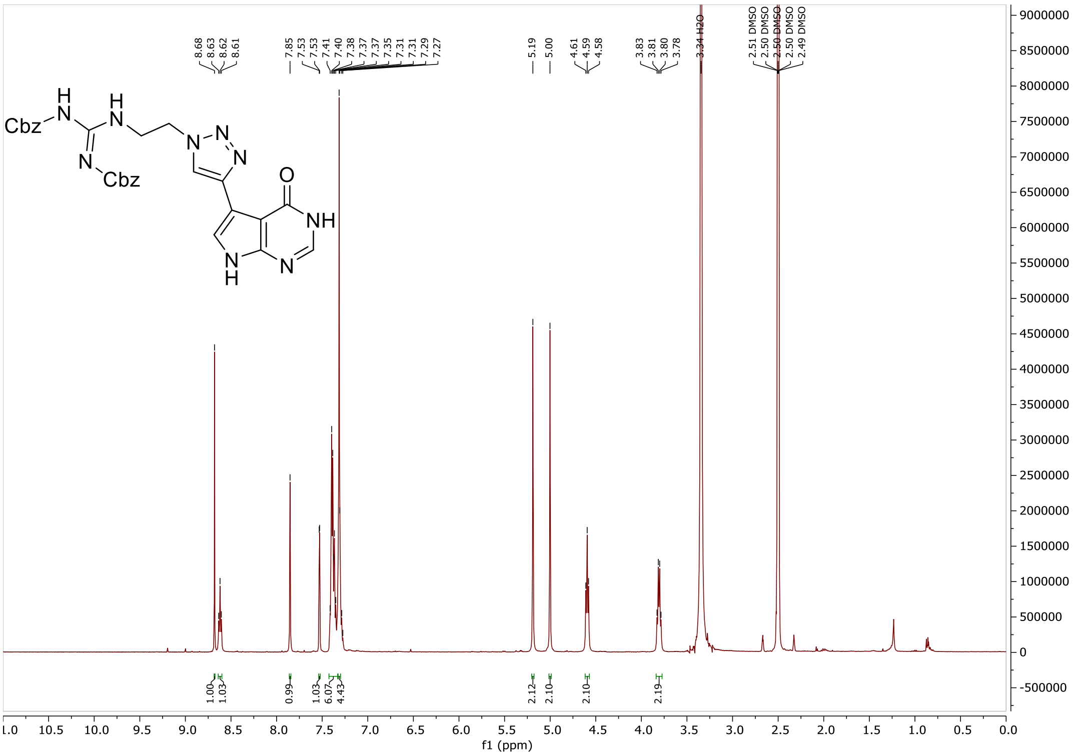

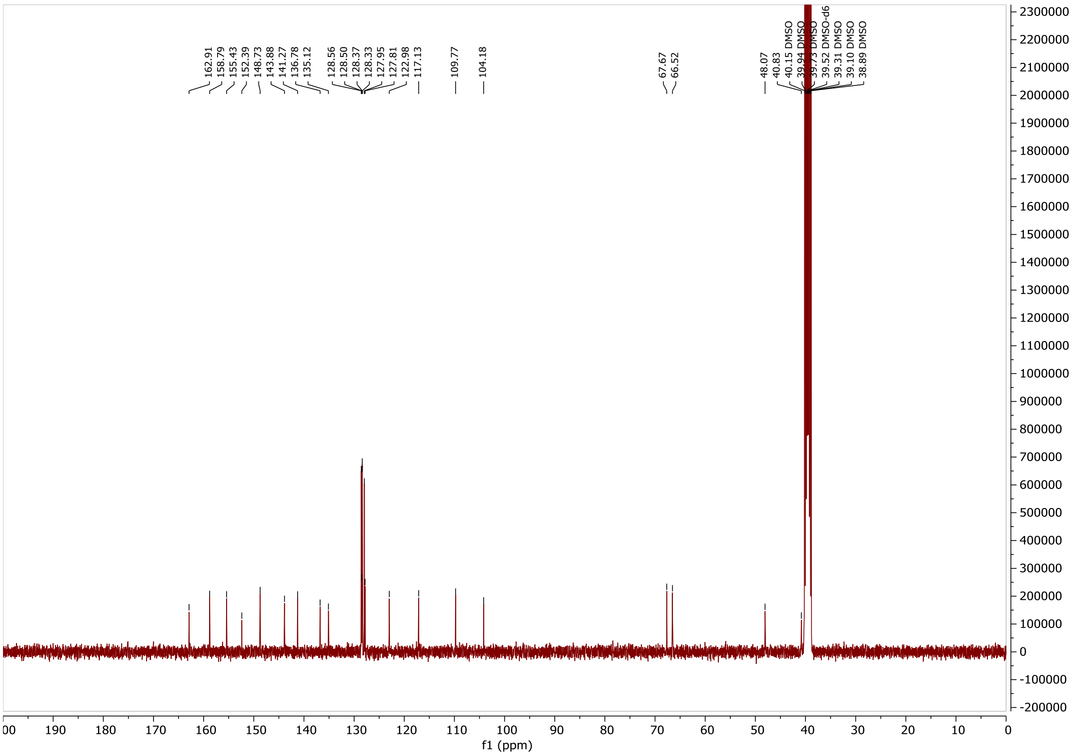

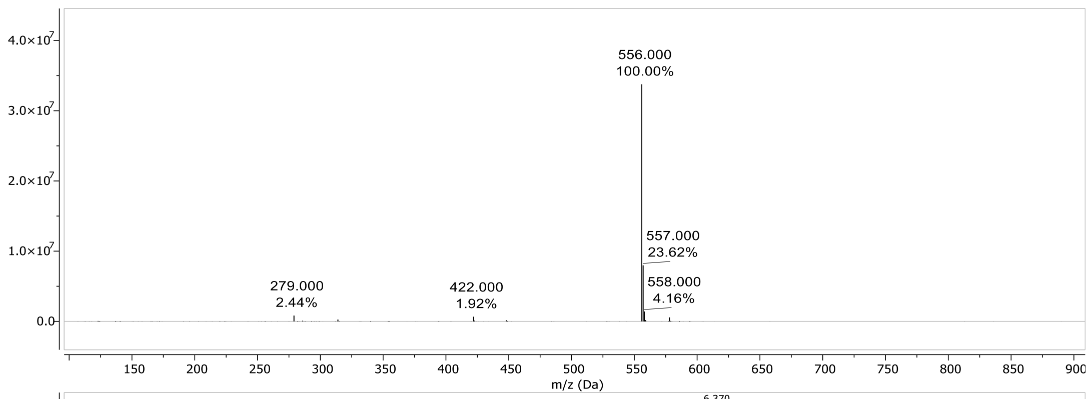

4.0×10^7
3.0×10^7
2.0×10^7
1.0×10^7
0.0
556.000
100.00%
557.000
23.62%
558.000
4.16%
279.000
2.44%
422.000
1.92%
150 200 250 300 350 400 450 500 550 600 650 700 750 800 850 900
m/z (Da)

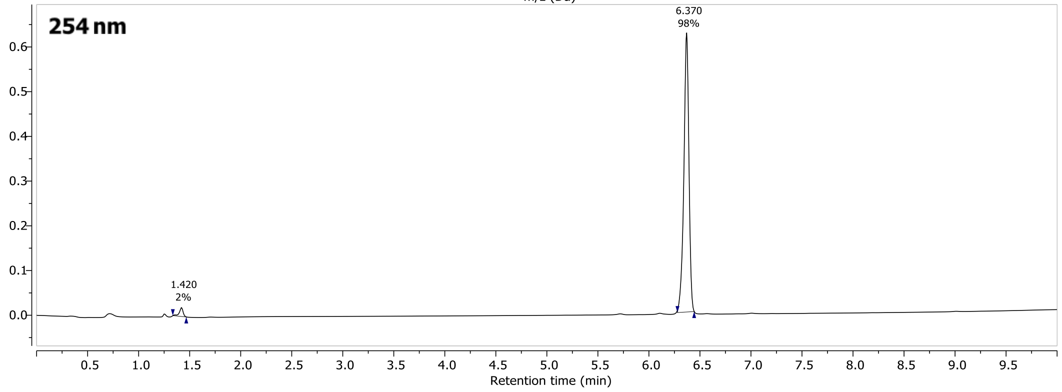

254 nm
0.6
0.5
0.4
0.3
0.2
0.1
0.0
6.370
98%
1.420
2%
0.5 1.0 1.5 2.0 2.5 3.0 3.5 4.0 4.5 5.0 5.5 6.0 6.5 7.0 7.5 8.0 8.5 9.0 9.5
Retention time (min)

Compound 8b

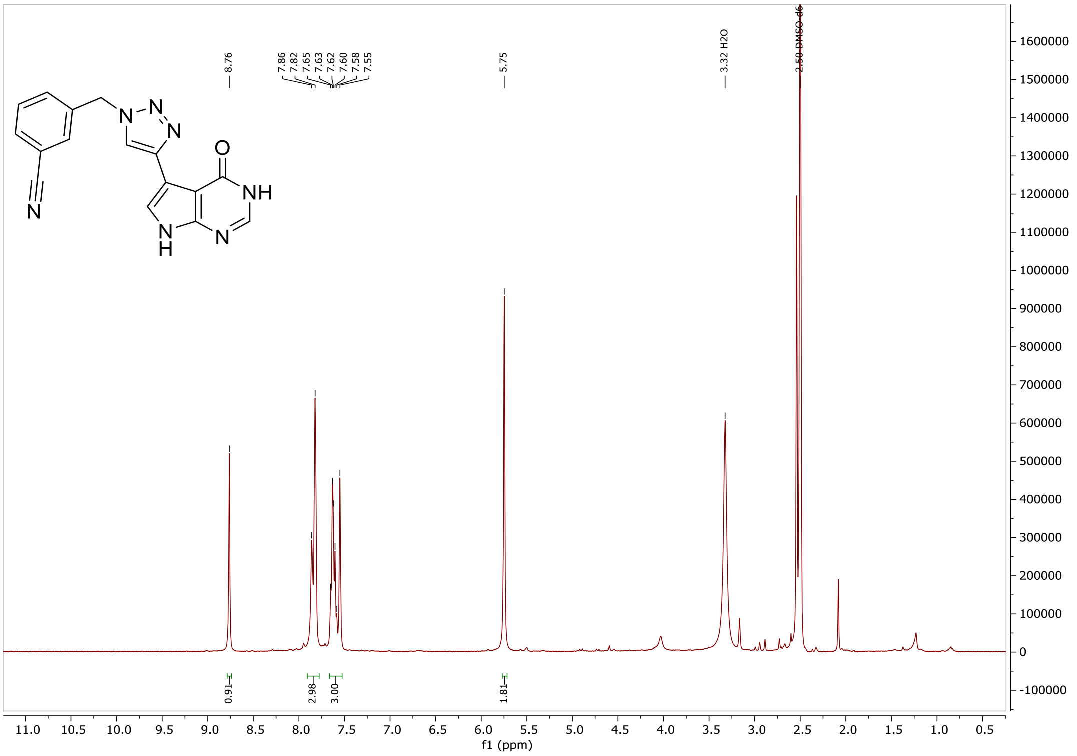
8.76
7.86
7.82
7.65
7.63
7.62
7.60
7.58
7.55
5.75
3.32 H2O
2.50 DMSO-d6
0.91
2.98
3.00
1.81
f1 (ppm)

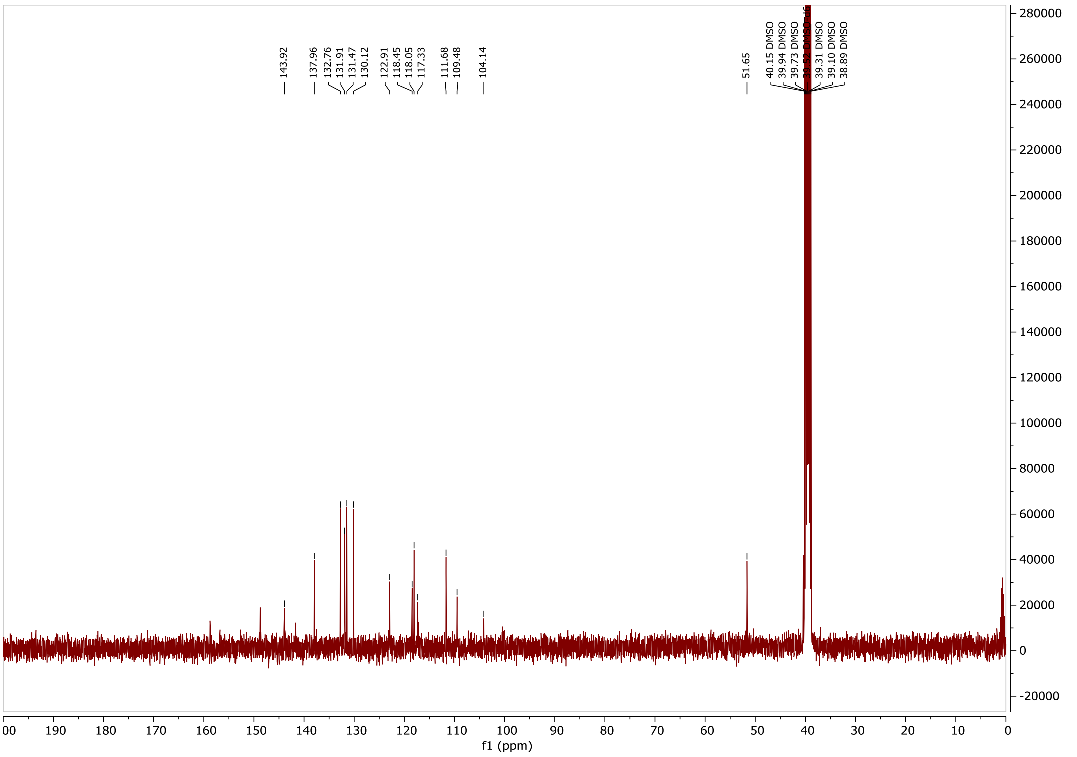
143.92
137.96
132.76
131.91
131.47
130.12
122.91
118.45
118.05
117.33
111.68
109.48
104.14
51.65
40.15 DMSO
39.94 DMSO
39.73 DMSO
39.31 DMSO
39.10 DMSO
38.89 DMSO
f1 (ppm)

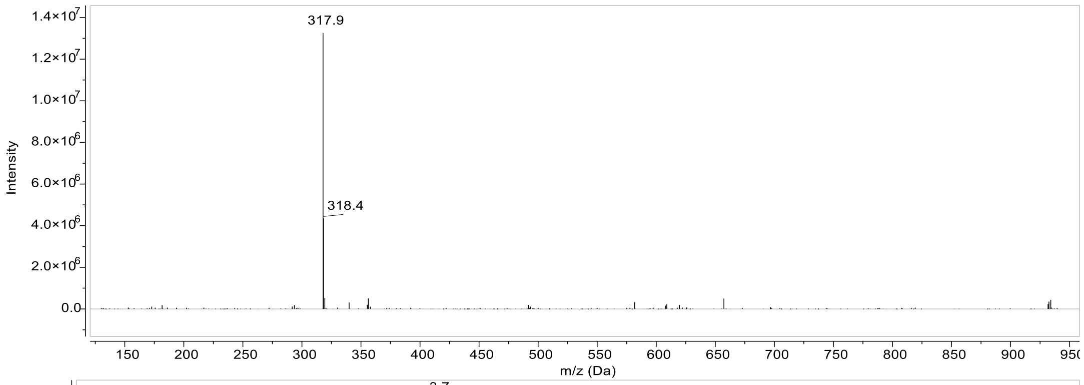
317.9
318.4
Intensity
m/z (Da)

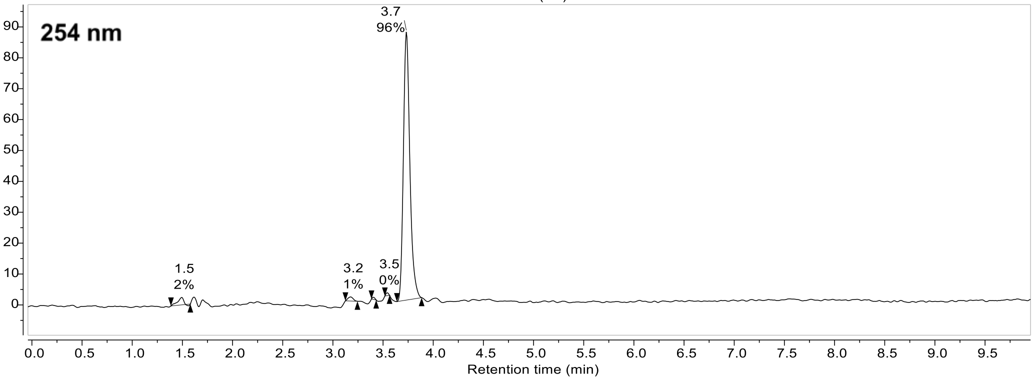
254 nm
1.5
2%
3.2
1%
3.5
0%
3.7
96%
Retention time (min)

Compound 8c

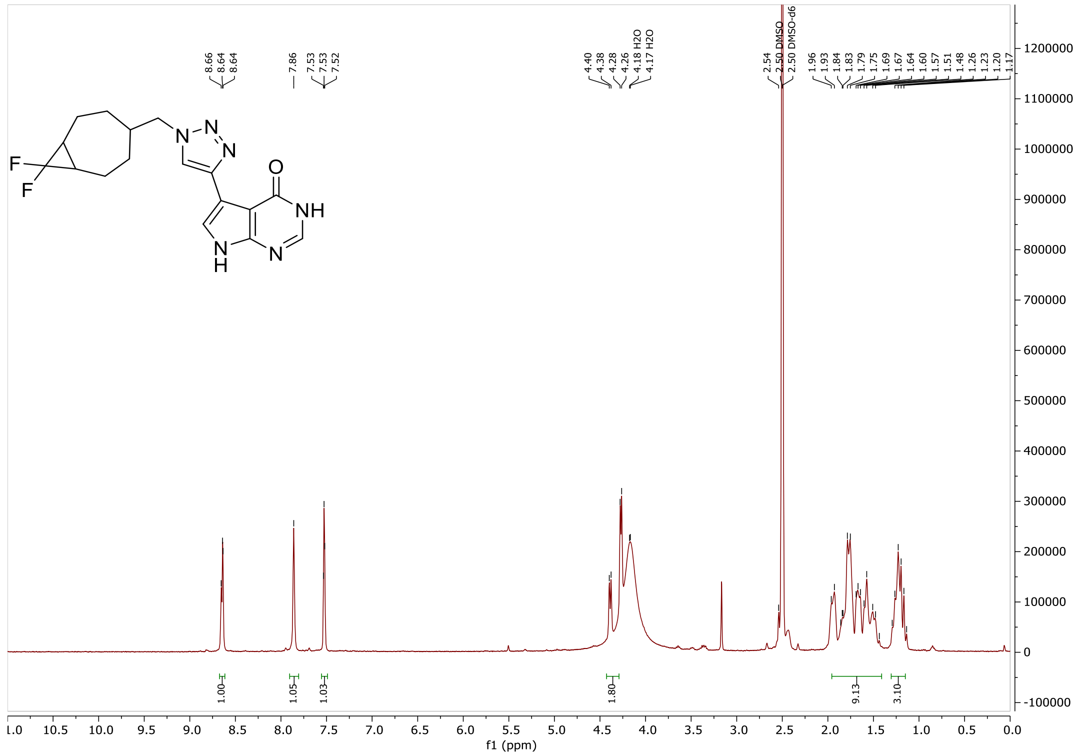
F
F
N
N
N
O
NH
N
H
8.66
8.64
8.64
7.86
7.53
7.53
7.52
4.40
4.38
4.28
4.26
4.18 H2O
4.17 H2O
2.54
2.50 DMSO
2.50 DMSO-d6
1.96
1.93
1.84
1.83
1.79
1.75
1.69
1.67
1.64
1.60
1.57
1.51
1.48
1.26
1.23
1.20
1.17
1.00
1.05
1.03
1.80
9.13
3.10
f1 (ppm)

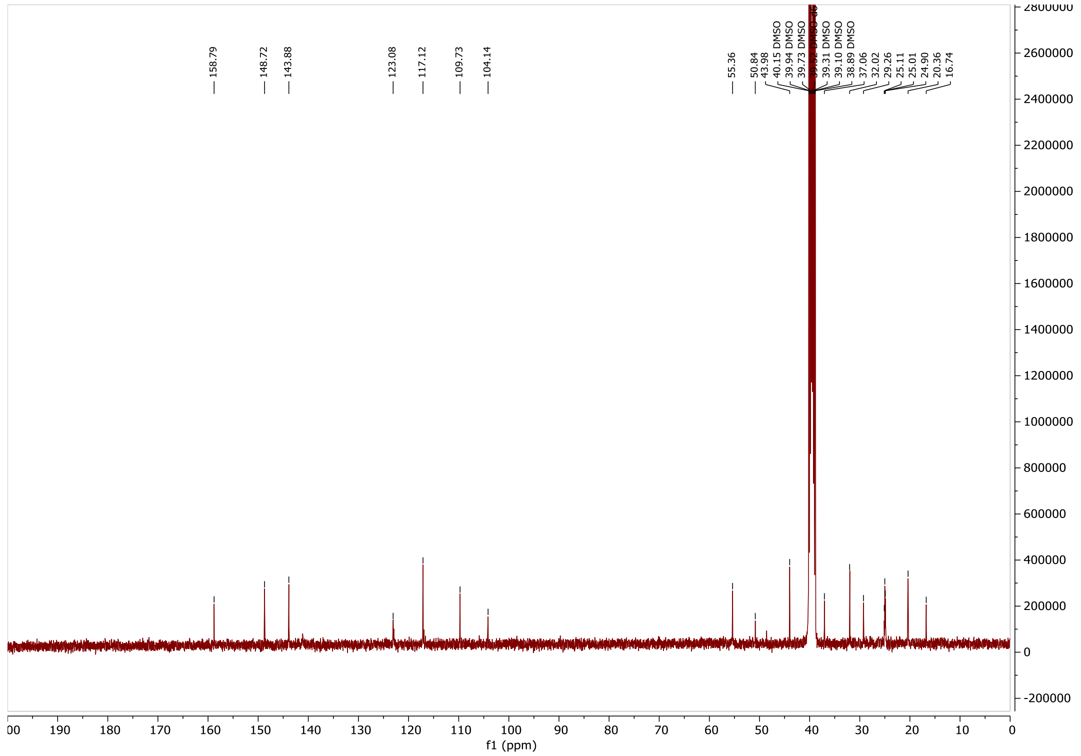
158.79
148.72
143.88
123.08
117.12
109.73
104.14
55.36
50.84
43.98
40.15 DMSO
39.94 DMSO
39.73 DMSO
39.31 DMSO
39.10 DMSO
38.89 DMSO
37.06
32.02
29.26
25.11
25.01
24.90
20.36
16.74
f1 (ppm)

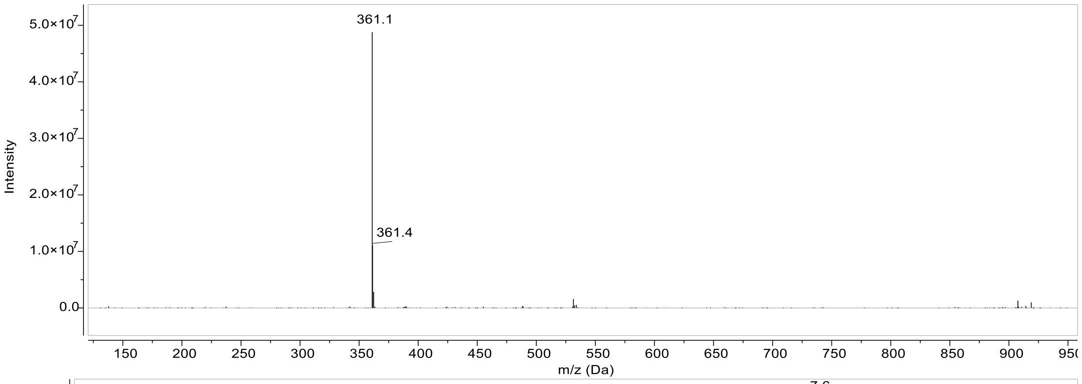
361.1
361.4
Intensity
m/z (Da)

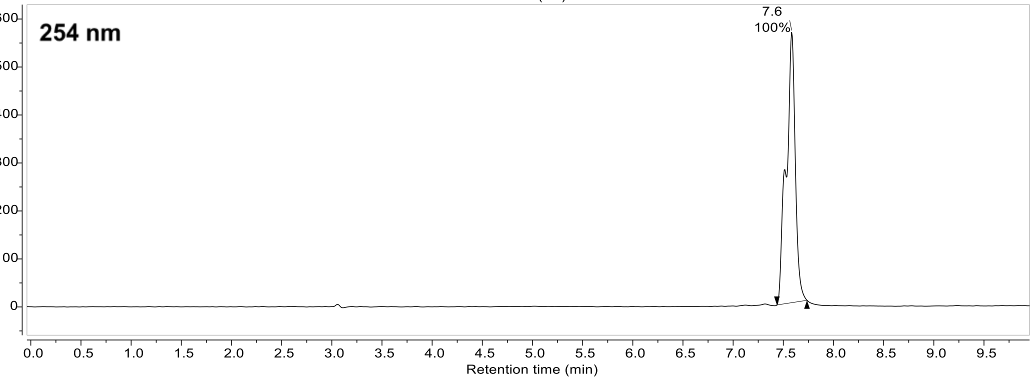
254 nm
7.6
100%
Retention time (min)

Compound 8d

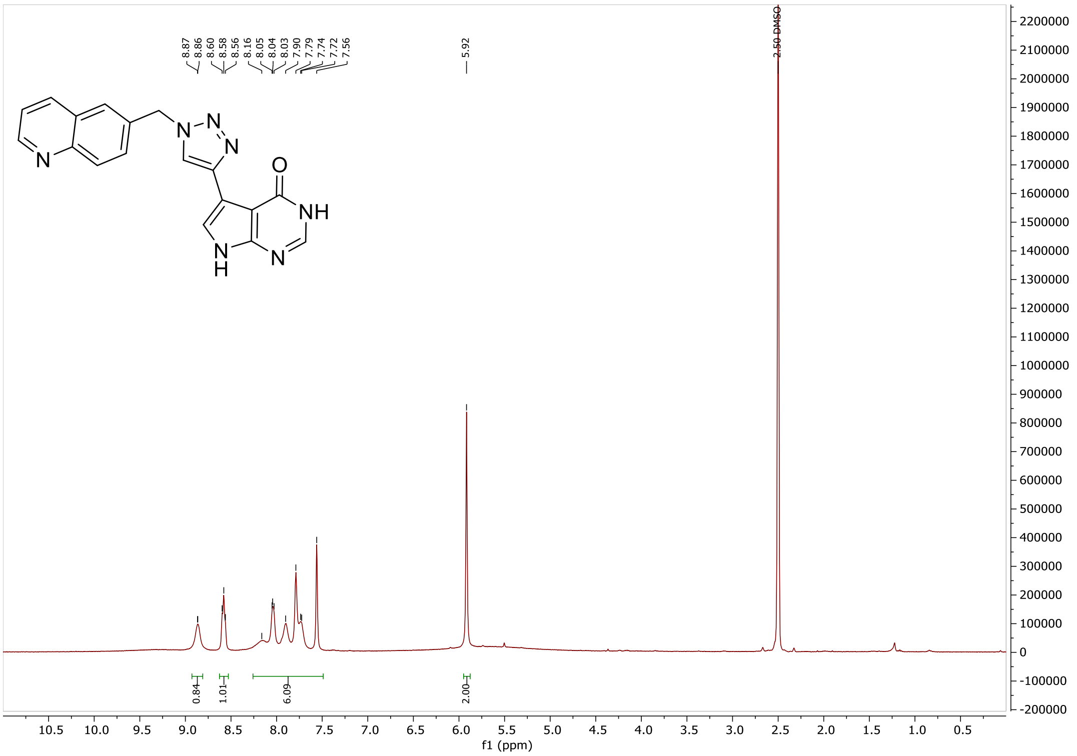
8.87
8.86
8.60
8.58
8.56
8.16
8.05
8.04
8.03
7.90
7.79
7.74
7.72
7.56
5.92
2.50 DMSO
0.84
1.01
6.09
2.00
f1 (ppm)

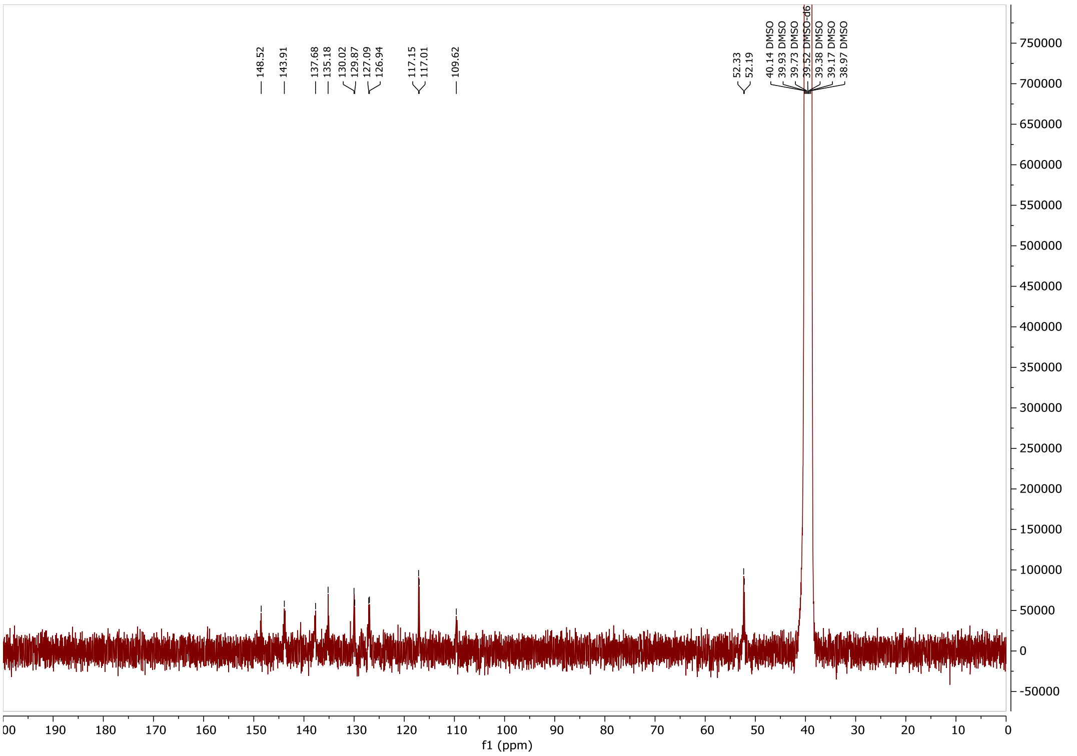
148.52
143.91
137.68
135.18
130.02
129.87
127.09
126.94
117.15
117.01
109.62
52.33
52.19
40.14 DMSO
39.93 DMSO
39.73 DMSO
39.52 DMSO-d6
39.38 DMSO
39.17 DMSO
38.97 DMSO
f1 (ppm)

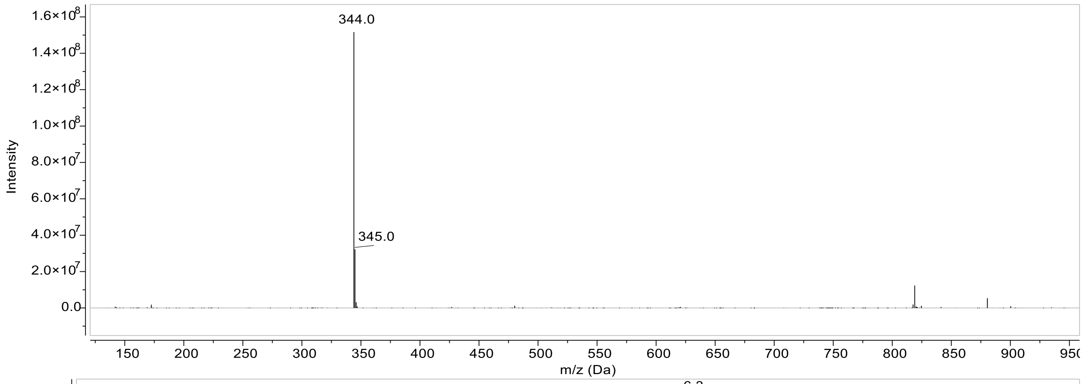

344.0
345.0
Intensity
m/z (Da)

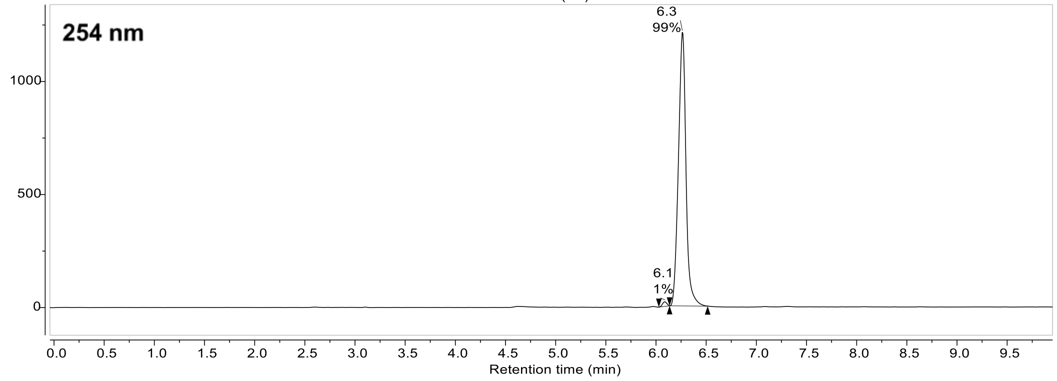

254 nm
6.3
99%
6.1
1%
Retention time (min)

Compound 8e

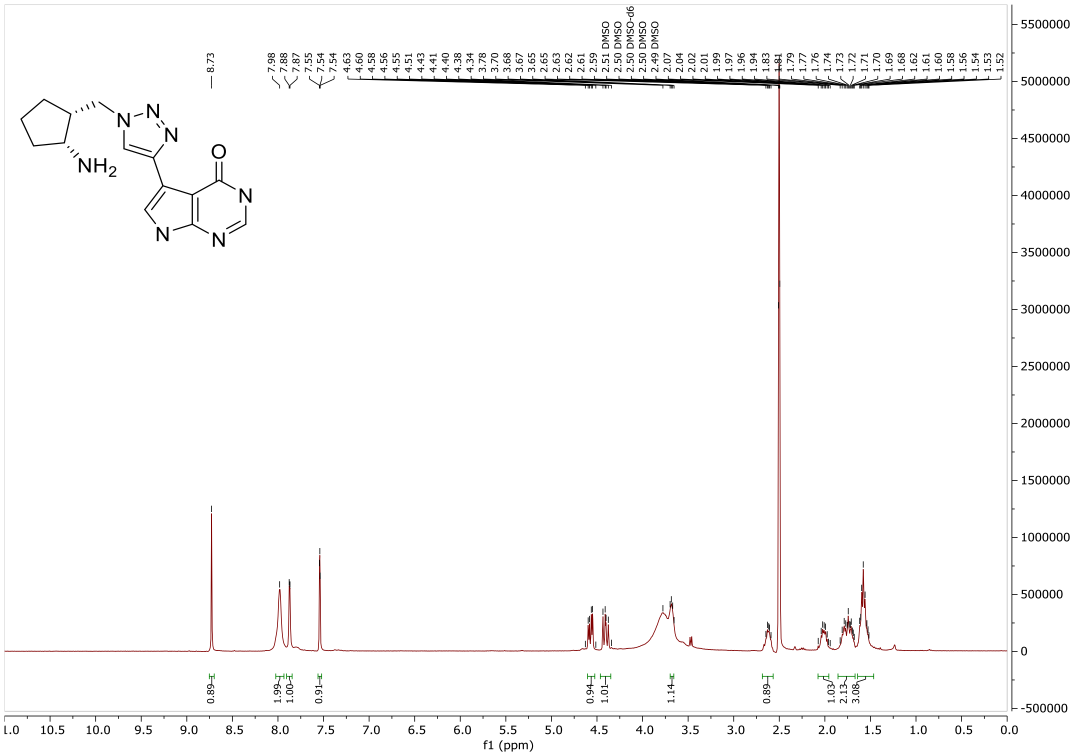
8.73
7.98
7.88
7.87
7.55
7.54
7.54
4.63
4.60
4.58
4.56
4.55
4.51
4.43
4.41
4.40
4.38
4.34
3.78
3.70
3.68
3.67
3.65
2.65
2.63
2.62
2.61
2.59
2.51 DMSO
2.50 DMSO
2.50 DMSO-d6
2.50 DMSO
2.49 DMSO
2.07
2.04
2.02
2.01
1.99
1.97
1.96
1.94
1.83
1.81
1.79
1.77
1.76
1.74
1.73
1.72
1.71
1.70
1.69
1.68
1.62
1.61
1.60
1.58
1.56
1.54
1.53
1.52
NH2
O
N
0.89
1.99
1.00
0.91
0.94
1.01
1.14
0.89
1.03
2.13
3.08
f1 (ppm)

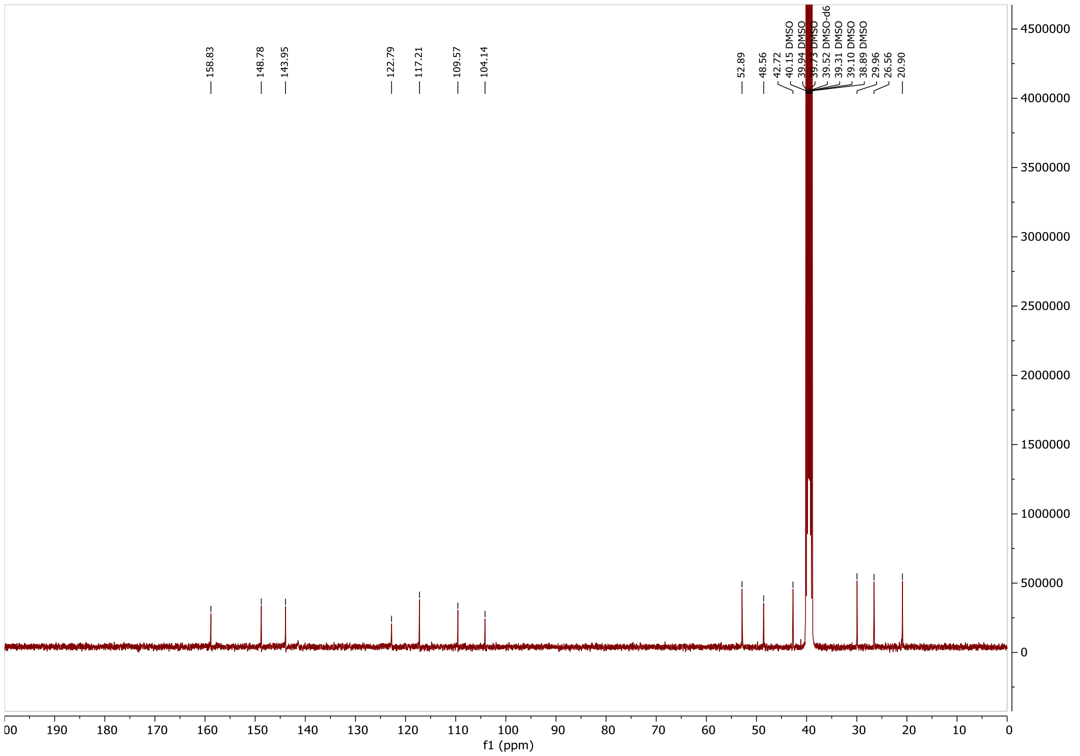
158.83
148.78
143.95
122.79
117.21
109.57
104.14
52.89
48.56
42.72
40.15 DMSO
39.94 DMSO
39.73 DMSO-d6
39.52 DMSO
39.31 DMSO
39.10 DMSO
38.89 DMSO
29.96
26.56
20.90
f1 (ppm)

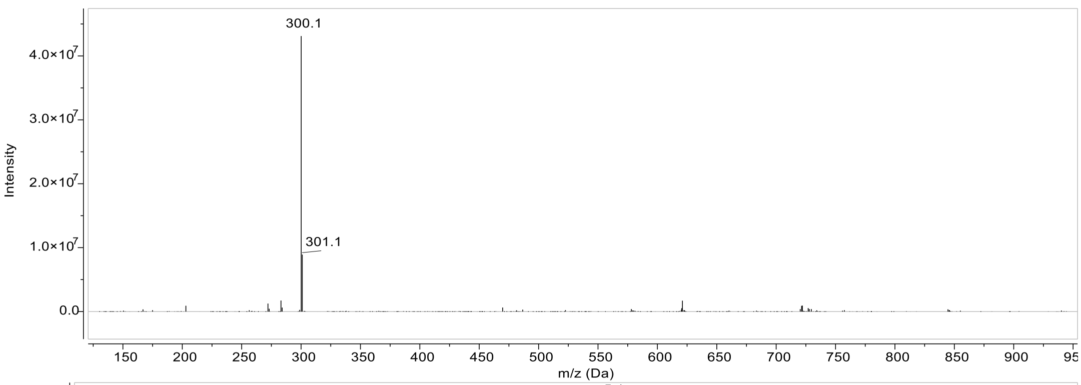
300.1
301.1
Intensity
4.0×10^7
3.0×10^7
2.0×10^7
1.0×10^7
0.0
150 200 250 300 350 400 450 500 550 600 650 700 750 800 850 900 95
m/z (Da)

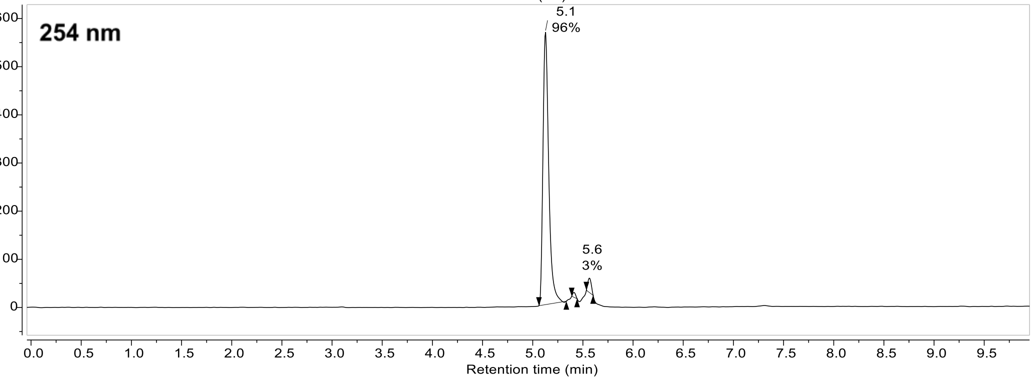
254 nm
5.1
96%
5.6
3%
600
500
400
300
200
100
0
0.0 0.5 1.0 1.5 2.0 2.5 3.0 3.5 4.0 4.5 5.0 5.5 6.0 6.5 7.0 7.5 8.0 8.5 9.0 9.5
Retention time (min)

Compound 4

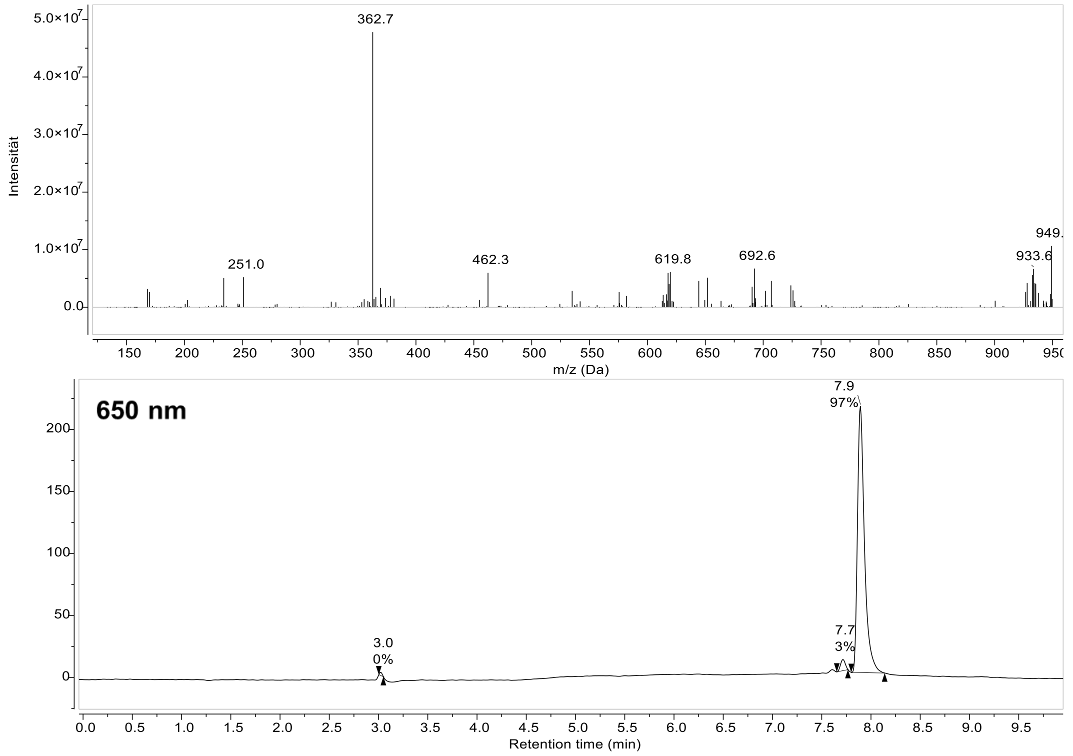

Compound 3

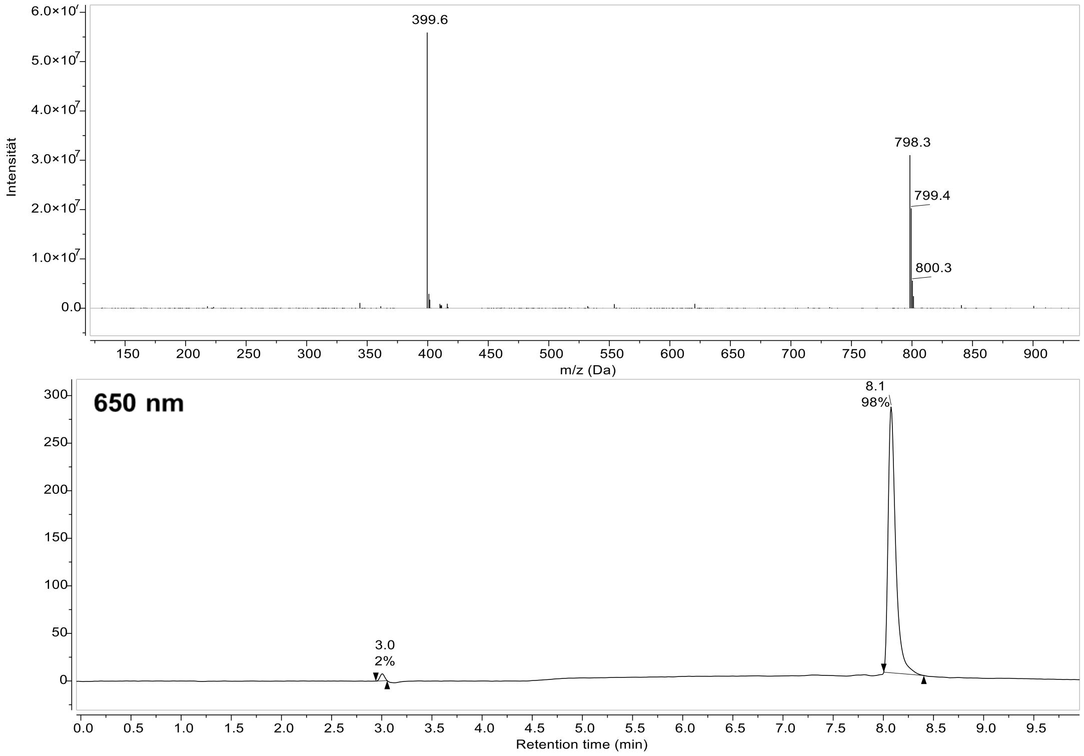

Supplement: Supplementary file 1 [file jm5c02323_si_001.pdf]
